# Supplementary material for: Performance comparison of systemic activity correction in functional near-infrared spectroscopy for methods with and without short distance channels
Source: Neurophotonics. 2022 Oct 12;10(1):013503. doi: 10.1117/1.NPh.10.1.013503 (PMC9555616; doi:10.1117/1.NPh.10.1.013503)
Supplement: Supplementary file 1 [file NPh_010_013503_SD001.pdf]

Supplementary Material

Statistical Results

Table 1 Descriptive Statistics of sRMSE resulting from the SIM data

	**SIM** $\Delta[HbO]$					
	NO SAC	CAR	GCR	SSR	GLM ALL	GLM BH
Mean	0.198	0.155	0.157	0.159	0.138	0.157
Std. Error of Mean	0.010	0.010	0.009	0.010	0.009	0.010
	**SIM** $\Delta[HbR]$					
	NO SAC	CAR	GCR	SSR	GLM ALL	GLM BH
Mean	0.169	0.153	0.160	0.163	0.147	0.155
Std. Error of Mean	0.010	0.009	0.009	0.009	0.007	0.009

Table 2 Results of the rmBANOVAs and corresponding post hoc tests with respect to the sRMSE analysis of SIM data of both $\Delta[HbO]$ and $\Delta[HbR]$. For all post hoc tests, the prior odds $P(M)$ were set to the default of 0.26, $P(M|data)$ represents the posterior odds, BF_{10} the Bayes factor and $error$ the error of the given test. $BF_{10} = 1$ indicates no evidence for neither H_0 nor H_1, $BF_{10} < 1$ indicates evidence in favor of the H_0, that is, that there are no differences between two methods and $BF_{10} > 1$ indicates evidence in favor of the H_1, that is, that there are differences between two methods. According to the guidelines of Lee and Wagenmakers (2014), Bayes factors BF_{10} can be categorized in the following way: $BF_{10} < 1/100$ extreme evidence for H_0, $1/100 < BF_{10} < 1/30$ very strong evidence for H_0, $1/30 < BF_{10} < 1/10$ strong evidence for H_0, $1/10 < BF_{10} < 1/3$ moderate evidence for H_0, $1/3 < BF_{10} < 1$ anecdotal evidence for H_0, $1 < BBF_{10} < 3$ anecdotal evidence for H_1, $3 < BF_{10} < 10$ moderate evidence for H_1, $10 < BF_{10} < 30$ strong evidence for H_1, $30 < BF_{10} < 100$ very strong evidence for H_1, $BF_{10} > 100$ extreme evidence for H_1.

		**SIM** $\Delta[HbO]$			
Models	$P(M)$	$P(M\|data)$	BF_M	BF_{10}	error %
Null model (incl. subject)	0.500	$1.590e-9$	$1.590e-9$	1.000	
METHOD	0.500	1.000	$6.290e+8$	$6.290e+8$	0.459
		Prior Odds	Posterior Odds	$BF_{10,U}$	error %
NO SAC	CAR	0.260	84.329	324.441	$2.719e-8$
	GCR	0.260	333.543	1283.249	$1.773e-8$
	SSR	0.260	21.207	81.589	$1.188e-4$
	GLM ALL	0.260	5792.003	22283.701	$2.704e-9$
	GLM BH	0.260	1282.865	4935.597	$3.068e-9$
CAR	GCR	0.260	0.063	0.242	0.023
	SSR	0.260	0.062	0.239	0.023
	GLM ALL	0.260	0.526	2.022	$1.498e-6$
	GLM BH	0.260	0.059	0.228	0.023
GCR	SSR	0.260	0.058	0.223	0.022
	GLM ALL	0.260	1.774	6.823	$1.309e-7$
	GLM BH	0.260	0.057	0.219	0.022
SSR	GLM ALL	0.260	5.514	21.214	$1.287e-7$
	GLM BH	0.260	0.058	0.225	0.022
GLM ALL	GLM BH	0.260	3.229	12.424	$9.193e-8$
		**SIM** $\Delta[HbR]$			
Models	$P(M)$	$P(M\|data)$	BF_M	BF_{10}	error %
Null model (incl. subject)	0.500	0.663	1.966	1.000	
METHOD	0.500	0.337	0.509	0.509	0.554

Table 3 Descriptive Statistics of sRMSE resulting from the ME data

			**ME** $\Delta[HbO]$			
	NO SAC	CAR	GCR	SSR	GLM ALL	GLM BH
Mean	0.212	0.227	0.229	0.215	0.211	0.214
Std. Error of Mean	0.006	0.007	0.006	0.008	0.007	0.007
			**ME** $\Delta[HbR]$			
	NO SAC	CAR	GCR	SSR	GLM ALL	GLM BH
Mean	0.213	0.219	0.219	0.216	0.222	0.216
Std. Error of Mean	0.009	0.007	0.008	0.008	0.008	0.008

Table 4 Results of the rmBANOVAs and corresponding post hoc tests with respect to the sRMSE analysis of ME data of both $\Delta[HbO]$ and $\Delta[HbR]$. For all post hoc tests, the prior odds $P(M)$ were set to the default of 0.26, $P(M|data)$ represents the posterior odds, BF_{10} the Bayes factor and $error$ the error of the given test. $BF_{10} = 1$ indicates no evidence for neither H_0 nor H_1, $BF_{10} < 1$ indicates evidence in favor of the H_0, that is, that there are no differences between two methods and $BF_{10} > 1$ indicates evidence in favor of the H_1, that is, that there are differences between two methods. According to the guidelines of Lee and Wagenmakers (2014), Bayes factors BF_{10} can be categorized in the following way: $BF_{10} < 1/100$ extreme evidence for H_0, $1/100 < BF_{10} < 1/30$ very strong evidence for H_0, $1/30 < BF_{10} < 1/10$ strong evidence for H_0, $1/10 < BF_{10} < 1/3$ moderate evidence for H_0, $1/3 < BF_{10} < 1$ anecdotal evidence for H_0, $1 < BBF_{10} < 3$ anecdotal evidence for H_1, $3 < BF_{10} < 10$ moderate evidence for H_1, $10 < BF_{10} < 30$ strong evidence for H_1, $30 < BF_{10} < 100$ very strong evidence for H_1, $BF_{10} > 100$ extreme evidence for H_1.

		**ME** $\Delta[HbO]$			
Models	$P(M)$	$P(M\|data)$	BF$_M$	BF$_{10}$	error %
Null model (incl. subject)	0.500	0.004	0.005	1.000	
METHOD	0.500	0.996	221.233	221.233	0.356
		Prior Odds	Posterior Odds	BF$_{10,U}$	error %
NO SAC	CAR	0.260	4.968	19.115	$8.880e-8$
	GCR	0.260	4.472	17.205	$8.082e-8$
	SSR	0.260	0.061	0.233	0.024
	GLM ALL	0.260	0.057	0.218	0.024
	GLM BH	0.260	0.062	0.239	0.024
CAR	GCR	0.260	0.066	0.253	0.025
	SSR	0.260	0.432	1.661	$1.960e-6$
	GLM ALL	0.260	30.016	115.482	$1.935e-4$
	GLM BH	0.260	3.148	12.111	$7.139e-8$
GCR	SSR	0.260	0.455	1.750	$1.824e-6$
	GLM ALL	0.260	11.200	43.090	$6.961e-4$
	GLM BH	0.260	4.192	16.127	$7.596e-8$
SSR	GLM ALL	0.260	0.074	0.286	0.025
	GLM BH	0.260	0.056	0.216	0.024
GLM ALL	GLM BH	0.260	0.078	0.298	0.025
		**ME** $\Delta[HbR]$			
Models	$P(M)$	$P(M\|data)$	BF$_M$	BF$_{10}$	error %
Null model (incl. subject)	0.500	0.890	8.120	1.000	
METHOD	0.500	0.110	0.123	0.123	0.481

Table 5 Descriptive Statistics of sRMSE resulting from the MI data

			**MI** $\Delta[HbO]$			
	NO SAC	CAR	GCR	SSR	GLM ALL	GLM BH
Mean	0.236	0.244	0.243	0.242	0.240	0.242
Std. Error of Mean	0.003	0.003	0.003	0.003	0.003	0.003
			**MI** $\Delta[HbR]$			
	NO SAC	CAR	GCR	SSR	GLM ALL	GLM BH
Mean	0.245	0.248	0.249	0.246	0.247	0.246
Std. Error of Mean	0.002	0.002	0.002	0.003	0.002	0.002

Table 6 Results of the rmBANOVAs and corresponding post hoc tests with respect to the sRMSE analysis of MI data of both $\Delta[HbO]$ and $\Delta[HbR]$. $P(M|data)$ represents the posterior odds, BF_{10} the Bayes factor and $error$ the error of the given test. $BF_{10} = 1$ indicates no evidence for neither H_0 nor H_1, $BF_{10} < 1$ indicates evidence in favor of the H_0, that is, that there are no differences between two methods and $BF_{10} > 1$ indicates evidence in favor of the H_1, that is, that there are differences between two methods. According to the guidelines of Lee and Wagenmakers (2014), Bayes factors BF_{10} can be categorized in the following way: $BF_{10} < 1/100$ extreme evidence for H_0, $1/100 < BF_{10} < 1/30$ very strong evidence for H_0, $1/30 < BF_{10} < 1/10$ strong evidence for H_0, $1/10 < BF_{10} < 1/3$ moderate evidence for H_0, $1/3 < BF_{10} < 1$ anecdotal evidence for H_0, $1 < BBF_{10} < 3$ anecdotal evidence for H_1, $3 < BF_{10} < 10$ moderate evidence for H_1, $10 < BF_{10} < 30$ strong evidence for H_1, $30 < BF_{10} < 100$ very strong evidence for H_1, $BF_{10} > 100$ extreme evidence for H_1.

		**MI** $\Delta[HbO]$			
Models	$P(M)$	$P(M\|data)$	BF$_M$	BF$_{10}$	error %
Null model (incl. subject)	0.500	0.610	1.564	1.000	
METHOD	0.500	0.390	0.639	0.639	0.294
		**MI** $\Delta[HbR]$			
Models	$P(M)$	$P(M\|data)$	BF$_M$	BF$_{10}$	error %
Null model (incl. subject)	0.500	0.909	9.959	1.000	
METHOD	0.500	0.091	0.100	0.100	0.654

Table 7 Descriptive Statistics of COR resulting from the SIM data.

			**SIM** $\Delta[HbO]$			
	NO SAC	CAR	GCR	SSR	GLM ALL	GLM BH
Mean	0.852	1.307	1.291	1.259	1.578	1.254
Std. Error of Mean	0.103	0.099	0.096	0.093	0.077	0.103
			**SIM** $\Delta[HbR]$			
	NO SAC	CAR	GCR	SSR	GLM ALL	GLM BH
Mean	1.243	1.400	1.337	1.305	1.460	1.380
Std. Error of Mean	0.083	0.077	0.070	0.089	0.070	0.080

Table 8 Results of the rmBANOVAs and corresponding post hoc tests with respect to the COR analysis of SIM data of both $\Delta[HbO]$ and $\Delta[HbR]$. For all post hoc tests, the prior odds $P(M)$ were set to the default of 0.26, $P(M|data)$ represents the posterior odds, BF_{10} the Bayes factor and $error$ the error of the given test. $BF_{10} = 1$ indicates no evidence for neither H_0 nor H_1, $BF_{10} < 1$ indicates evidence in favor of the H_0, that is, that there are no differences between two methods and $BF_{10} > 1$ indicates evidence in favor of the H_1, that is, that there are differences between two methods. According to the guidelines of Lee and Wagenmakers (2014), Bayes factors BF_{10} can be categorized in the following way: $BF_{10} < 1/100$ extreme evidence for H_0, $1/100 < BF_{10} < 1/30$ very strong evidence for H_0, $1/30 < BF_{10} < 1/10$ strong evidence for H_0, $1/10 < BF_{10} < 1/3$ moderate evidence for H_0, $1/3 < BF_{10} < 1$ anecdotal evidence for H_0, $1 < BBF_{10} < 3$ anecdotal evidence for H_1, $3 < BF_{10} < 10$ moderate evidence for H_1, $10 < BF_{10} < 30$ strong evidence for H_1, $30 < BF_{10} < 100$ very strong evidence for H_1, $BF_{10} > 100$ extreme evidence for H_1.

		**SIM** $\Delta[HbO]$			
Models	$P(M)$	$P(M\|data)$	BF$_M$	BF$_{10}$	error %
Null model (incl. subject)	0.500	$6.805e-15$	$6.805e-15$	1.000	
METHOD	0.500	1.000	$1.470e+14$	$1.470e+14$	0.502
		Prior Odds	Posterior Odds	BF$_{10,U}$	error %
NO SAC	CAR	0.260	571.506	2198.769	$1.097e-8$
	GCR	0.260	1132.643	4357.641	$4.003e-9$
	SSR	0.260	107.664	414.217	$2.142e-8$
	GLM ALL	0.260	$3.257e+6$	$1.253e+7$	$4.115e-10$
	GLM BH	0.260	1859.217	7153.007	$1.046e-9$
CAR	GCR	0.260	0.063	0.243	0.023
	SSR	0.260	0.068	0.263	0.023
	GLM ALL	0.260	16.380	63.020	$1.796e-5$
	GLM BH	0.260	0.083	0.319	0.024
GCR	SSR	0.260	0.062	0.239	0.023
	GLM ALL	0.260	42.216	162.417	$6.414e-8$
	GLM BH	0.260	0.070	0.271	0.024
SSR	GLM ALL	0.260	1108.489	4264.716	$4.172e-9$
	GLM BH	0.260	0.057	0.220	0.022
GLM ALL	GLM BH	0.260	1255.218	4829.227	$3.222e-9$
		**SIM** $\Delta[HbR]$			
Models	$P(M)$	$P(M\|data)$	BF$_M$	BF$_{10}$	error %
Null model (incl. subject)	0.500	0.170	0.205	1.000	
METHOD	0.500	0.830	4.875	4.875	0.525
		Prior Odds	Posterior Odds	BF$_{10,U}$	error %
NO SAC	CAR	0.260	0.480	1.847	$1.722e-6$
	GCR	0.260	0.120	0.460	0.025
	SSR	0.260	0.122	0.470	0.025
	GLM ALL	0.260	30.999	119.264	$8.891e-8$
	GLM BH	0.260	3.304	12.711	$9.146e-8$
CAR	GCR	0.260	0.519	1.995	$1.530e-6$
	SSR	0.260	0.122	0.471	0.025
	GLM ALL	0.260	0.086	0.329	0.024
	GLM BH	0.260	0.060	0.232	0.023
GCR	SSR	0.260	0.062	0.238	0.023
	GLM ALL	0.260	0.277	1.066	0.023
	GLM BH	0.260	0.072	0.276	0.024
SSR	GLM ALL	0.260	0.617	2.373	$1.149e-6$
	GLM BH	0.260	0.123	0.473	0.025
GLM ALL	GLM BH	0.260	0.241	0.927	0.024

Table 9 Descriptive Statistics of COR resulting from the ME data.

	**ME** $\Delta[HbO]$					
	NO SAC	CAR	GCR	SSR	GLM ALL	GLM BH
Mean	0.722	0.626	0.582	0.771	0.848	0.754
Std. Error of Mean	0.081	0.077	0.077	0.079	0.074	0.080
	**ME** $\Delta[HbR]$					
	NO SAC	CAR	GCR	SSR	GLM ALL	GLM BH
Mean	0.864	0.774	0.740	0.823	0.837	0.864
Std. Error of Mean	0.084	0.070	0.075	0.077	0.071	0.080

Table 10 Results of the rmBANOVAs and corresponding post hoc tests with respect to the COR analysis of ME data of both $\Delta[HbO]$ and $\Delta[HbR]$. For all post hoc tests, the prior odds $P(M)$ were set to the default of 0.26, $P(M|data)$ represents the posterior odds, BF_{10} the Bayes factor and $error$ the error of the given test. $BF_{10} = 1$ indicates no evidence for neither H_0 nor H_1, $BF_{10} < 1$ indicates evidence in favor of the H_0, that is, that there are no differences between two methods and $BF_{10} > 1$ indicates evidence in favor of the H_1, that is, that there are differences between two methods. According to the guidelines of Lee and Wagenmakers (2014), Bayes factors BF_{10} can be categorized in the following way: $BF_{10} < 1/100$ extreme evidence for H_0, $1/100 < BF_{10} < 1/30$ very strong evidence for H_0, $1/30 < BF_{10} < 1/10$ strong evidence for H_0, $1/10 < BF_{10} < 1/3$ moderate evidence for H_0, $1/3 < BF_{10} < 1$ anecdotal evidence for H_0, $1 < BBF_{10} < 3$ anecdotal evidence for H_1, $3 < BF_{10} < 10$ moderate evidence for H_1, $10 < BF_{10} < 30$ strong evidence for H_1, $30 < BF_{10} < 100$ very strong evidence for H_1, $BF_{10} > 100$ extreme evidence for H_1.

		**ME** $\Delta[HbO]$			
Models	$P(M)$	$P(M\|data)$	BF_M	BF_{10}	error %
Null model (incl. subject)	0.500	0.003	0.003	1.000	
METHOD	0.500	0.997	298.511	298.511	0.323
		Prior Odds	Posterior Odds	$\text{BF}_{10,U}$	error %
NO SAC	CAR	0.260	0.137	0.528	0.026
	GCR	0.260	0.212	0.814	0.025
	SSR	0.260	0.069	0.266	0.025
	GLM ALL	0.260	0.405	1.559	$2.133e-6$
	GLM BH	0.260	0.065	0.248	0.025
CAR	GCR	0.260	0.141	0.541	0.026
	SSR	0.260	0.783	3.012	$7.548e-7$
	GLM ALL	0.260	120.828	464.864	$1.788e-8$
	GLM BH	0.260	0.847	3.260	$6.498e-7$
GCR	SSR	0.260	1.680	6.465	$1.364e-7$
	GLM ALL	0.260	32.725	125.903	$2.494e-4$
	GLM BH	0.260	1.599	6.153	$1.541e-7$
SSR	GLM ALL	0.260	0.138	0.531	0.026
	GLM BH	0.260	0.059	0.227	0.024
GLM ALL	GLM BH	0.260	0.699	2.688	$9.273e-7$
		**ME** $\Delta[HbR]$			
Models	$P(M)$	$P(M\|data)$	BF_M	BF_{10}	error %
Null model (incl. subject)	0.500	0.405	0.682	1.000	
METHOD	0.500	0.595	1.467	1.467	0.526

Table 11 Descriptive Statistics of COR resulting from the MI data.

	**MI** $\Delta[HbO]$					
	NO SAC	CAR	GCR	SSR	GLM ALL	GLM BH
Mean	0.332	0.216	0.200	0.271	0.289	0.253
Std. Error of Mean	0.049	0.045	0.045	0.035	0.046	0.042
	**MI** $\Delta[HbR]$					
	NO SAC	CAR	GCR	SSR	GLM ALL	GLM BH
Mean	0.238	0.162	0.134	0.191	0.222	0.229
Std. Error of Mean	0.045	0.043	0.039	0.043	0.046	0.043

Table 12 Results of the rmBANOVAs and corresponding post hoc tests with respect to the COR analysis of MI data of both $\Delta[HbO]$ and $\Delta[HbR]$. $P(M|data)$ represents the posterior odds, BF_{10} the Bayes factor and $error$ the error of the given test. $BF_{10} = 1$ indicates no evidence for neither H_0 nor H_1, $BF_{10} < 1$ indicates evidence in favor of the H_0, that is, that there are no differences between two methods and $BF_{10} > 1$ indicates evidence in favor of the H_1, that is, that there are differences between two methods. According to the guidelines of Lee and Wagenmakers (2014), Bayes factors BF_{10} can be categorized in the following way: $BF_{10} < 1/100$ extreme evidence for H_0, $1/100 < BF_{10} < 1/30$ very strong evidence for H_0, $1/30 < BF_{10} < 1/10$ strong evidence for H_0, $1/10 < BF_{10} < 1/3$ moderate evidence for H_0, $1/3 < BF_{10} < 1$ anecdotal evidence for H_0, $1 < BBF_{10} < 3$ anecdotal evidence for H_1, $3 < BF_{10} < 10$ moderate evidence for H_1, $10 < BF_{10} < 30$ strong evidence for H_1, $30 < BF_{10} < 100$ very strong evidence for H_1, $BF_{10} > 100$ extreme evidence for H_1.

		**MI** $\Delta[HbO]$			
Models	$P(M)$	$P(M\|data)$	BF_M	BF_{10}	error %
Null model (incl. subject)	0.500	0.256	0.345	1.000	
METHOD	0.500	0.744	2.900	2.900	0.325
		**MI** $\Delta[HbR]$			
Models	$P(M)$	$P(M\|data)$	BF_M	BF_{10}	error %
Null model (incl. subject)	0.500	0.441	0.788	1.000	
METHOD	0.500	0.559	1.269	1.269	0.276

Table 13 Descriptive Statistics of CNR resulting from the SIM data.

	**SIM** $\Delta[HbO]$					
	NO SAC	CAR	GCR	SSR	GLM ALL	GLM BH
Mean	10.178	16.073	14.768	16.747	21.791	15.717
Std. Error of Mean	1.359	1.910	1.727	2.016	1.169	1.737
	**SIM** $\Delta[HbR]$					
	NO SAC	CAR	GCR	SSR	GLM ALL	GLM BH
Mean	15.254	18.220	18.330	16.580	19.073	17.647
Std. Error of Mean	1.613	1.685	1.831	1.449	1.690	1.694

Table 14 Results of the rmBANOVAs and corresponding post hoc tests with respect to the CNR analysis of SIM data of both $\Delta[HbO]$ and $\Delta[HbR]$. For all post hoc tests, the prior odds $P(M)$ were set to the default of 0.26, $P(M|data)$ represents the posterior odds, BF_{10} the Bayes factor and $error$ the error of the given test. $BF_{10} = 1$ indicates no evidence for neither H_0 nor H_1, $BF_{10} < 1$ indicates evidence in favor of the H_0, that is, that there are no differences between two methods and $BF_{10} > 1$ indicates evidence in favor of the H_1, that is, that there are differences between two methods. According to the guidelines of Lee and Wagenmakers (2014), Bayes factors BF_{10} can be categorized in the following way: $BF_{10} < 1/100$ extreme evidence for H_0, $1/100 < BF_{10} < 1/30$ very strong evidence for H_0, $1/30 < BF_{10} < 1/10$ strong evidence for H_0, $1/10 < BF_{10} < 1/3$ moderate evidence for H_0, $1/3 < BF_{10} < 1$ anecdotal evidence for H_0, $1 < BBF_{10} < 3$ anecdotal evidence for H_1, $3 < BF_{10} < 10$ moderate evidence for H_1, $10 < BF_{10} < 30$ strong evidence for H_1, $30 < BF_{10} < 100$ very strong evidence for H_1, $BF_{10} > 100$ extreme evidence for H_1.

		**SIM** $\Delta[HbO]$			
Null model (incl. subject)	0.500	$4.632e-6$	$4.632e-6$	1.000	
METHOD	0.500	1.000	215906.220	215906.220	0.297
		Prior Odds	Posterior Odds	BF$_{10,U}$	error %
NO SAC	CAR	0.260	3.042	11.704	$9.352e-8$
	GCR	0.260	1.484	5.710	$1.719e-7$
	SSR	0.260	3.178	12.227	$9.221e-8$
	GLM ALL	0.260	248839.853	957367.066	$2.843e-10$
	GLM BH	0.260	11.586	44.575	$4.422e-4$
CAR	GCR	0.260	0.118	0.452	0.025
	SSR	0.260	0.060	0.232	0.023
	GLM ALL	0.260	1.689	6.498	$1.376e-7$
	GLM BH	0.260	0.058	0.223	0.022
GCR	SSR	0.260	0.096	0.368	0.025
	GLM ALL	0.260	10.513	40.449	$6.404e-4$
	GLM BH	0.260	0.065	0.251	0.023
SSR	GLM ALL	0.260	1.418	5.457	$1.916e-7$
	GLM BH	0.260	0.078	0.300	0.024
GLM ALL	GLM BH	0.260	9.730	37.434	$8.231e-4$
		**SIM** $\Delta[HbR]$			
Models	$P(M)$	$P(M|data)$	BF$_M$	BF$_{10}$	error %
Null model (incl. subject)	0.500	0.595	1.467	1.000	
METHOD	0.500	0.405	0.682	0.682	0.393

Table 15 Descriptive Statistics of CNR resulting from the ME data.

			**ME** $\Delta[HbO]$			
	NO SAC	CAR	GCR	SSR	GLM ALL	GLM BH
Mean	9.248	9.294	8.467	11.260	13.025	11.508
Std. Error of Mean	1.031	0.844	0.810	1.154	1.211	1.105
			**ME** $\Delta[HbR]$			
	NO SAC	CAR	GCR	SSR	GLM ALL	GLM BH
Mean	13.343	12.192	11.731	13.166	14.383	13.822
Std. Error of Mean	1.060	0.819	0.762	0.994	0.934	1.066

Table 16 Results of the rmBANOVAs and corresponding post hoc tests with respect to the CNR analysis of ME data of both $\Delta[HbO]$ and $\Delta[HbR]$. For all post hoc tests, the prior odds $P(M)$ were set to the default of 0.26, $P(M|data)$ represents the posterior odds, BF_{10} the Bayes factor and $error$ the error of the given test. $BF_{10} = 1$ indicates no evidence for neither H_0 nor H_1, $BF_{10} < 1$ indicates evidence in favor of the H_0, that is, that there are no differences between two methods and $BF_{10} > 1$ indicates evidence in favor of the H_1, that is, that there are differences between two methods. According to the guidelines of Lee and Wagenmakers (2014), Bayes factors BF_{10} can be categorized in the following way: $BF_{10} < 1/100$ extreme evidence for H_0, $1/100 < BF_{10} < 1/30$ very strong evidence for H_0, $1/30 < BF_{10} < 1/10$ strong evidence for H_0, $1/10 < BF_{10} < 1/3$ moderate evidence for H_0, $1/3 < BF_{10} < 1$ anecdotal evidence for H_0, $1 < BBF_{10} < 3$ anecdotal evidence for H_1, $3 < BF_{10} < 10$ moderate evidence for H_1, $10 < BF_{10} < 30$ strong evidence for H_1, $30 < BF_{10} < 100$ very strong evidence for H_1, $BF_{10} > 100$ extreme evidence for H_1.

		**ME** $\Delta[HbO]$			
Null model (incl. subject)	0.500	$2.131e-7$	$2.131e-7$	1.000	
METHOD	0.500	1.000	$4.692e+6$	$4.692e+6$	0.330
		Prior Odds	Posterior Odds	$\text{BF}_{10,U}$	error %
NO SAC	CAR	0.260	0.056	0.215	0.024
	GCR	0.260	0.075	0.289	0.025
	SSR	0.260	0.782	3.008	$7.569e-7$
	GLM ALL	0.260	387.678	1491.523	$1.388e-8$
	GLM BH	0.260	5.136	19.759	$9.136e-8$
CAR	GCR	0.260	2.080	8.001	$9.751e-8$
	SSR	0.260	1.102	4.240	$3.771e-7$
	GLM ALL	0.260	128.127	492.947	$1.694e-8$
	GLM BH	0.260	7.763	29.867	$1.155e-7$
GCR	SSR	0.260	4.873	18.750	$8.731e-8$
	GLM ALL	0.260	267.328	1028.497	$1.436e-8$
	GLM BH	0.260	29.698	114.259	$1.866e-4$
SSR	GLM ALL	0.260	1.700	6.541	$1.326e-7$
	GLM BH	0.260	0.061	0.234	0.024
GLM ALL	GLM BH	0.260	5.758	22.153	$9.938e-8$
		**ME** $\Delta[HbR]$			
Models	$P(M)$	$P(M|data)$	BF_M	BF_{10}	error %
Null model (incl. subject)	0.500	0.010	0.010	1.000	
METHOD	0.500	0.990	103.042	103.042	0.298
		Prior Odds	Posterior Odds	$\text{BF}_{10,U}$	error %
NO SAC	CAR	0.260	0.180	0.693	0.025
	GCR	0.260	0.444	1.709	$1.886e-6$
	SSR	0.260	0.059	0.229	0.024
	GLM ALL	0.260	0.459	1.764	$1.803e-6$
	GLM BH	0.260	0.104	0.398	0.026
CAR	GCR	0.260	0.088	0.340	0.026
	SSR	0.260	0.138	0.529	0.026
	GLM ALL	0.260	8.889	34.200	$1.198e-7$
	GLM BH	0.260	0.320	1.232	0.023
GCR	SSR	0.260	0.404	1.556	$2.138e-6$
	GLM ALL	0.260	17.751	68.295	$6.716e-5$
	GLM BH	0.260	1.278	4.916	$2.684e-7$
SSR	GLM ALL	0.260	0.628	2.415	$1.114e-6$
	GLM BH	0.260	0.095	0.366	0.026
GLM ALL	GLM BH	0.260	0.081	0.313	0.025

Table 17 Descriptive Statistics of CNR resulting from the MI data.

	NO SAC	CAR	GCR	SSR	GLM ALL	GLM BH
			**MI** $\Delta[HbO]$			
Mean	6.012	5.434	5.307	5.962	6.511	6.061
Std. Error of Mean	0.507	0.529	0.512	0.377	0.573	0.544
			**MI** $\Delta[HbR]$			
	NO SAC	CAR	GCR	SSR	GLM ALL	GLM BH
Mean	6.490	5.982	5.819	6.336	6.795	6.569
Std. Error of Mean	0.574	0.478	0.440	0.585	0.524	0.453

Table 18 Results of the rmBANOVAs and corresponding post hoc tests with respect to the CNR analysis of MI data of both $\Delta[HbO]$ and $\Delta[HbR]$. $P(M|data)$ represents the posterior odds, BF_{10} the Bayes factor and $error$ the error of the given test. $BF_{10} = 1$ indicates no evidence for neither H_0 nor H_1, $BF_{10} < 1$ indicates evidence in favor of the H_0, that is, that there are no differences between two methods and $BF_{10} > 1$ indicates evidence in favor of the H_1, that is, that there are differences between two methods. According to the guidelines of Lee and Wagenmakers (2014), Bayes factors BF_{10} can be categorized in the following way: $BF_{10} < 1/100$ extreme evidence for H_0, $1/100 < BF_{10} < 1/30$ very strong evidence for H_0, $1/30 < BF_{10} < 1/10$ strong evidence for H_0, $1/10 < BF_{10} < 1/3$ moderate evidence for H_0, $1/3 < BF_{10} < 1$ anecdotal evidence for H_0, $1 < BBF_{10} < 3$ anecdotal evidence for H_1, $3 < BF_{10} < 10$ moderate evidence for H_1, $10 < BF_{10} < 30$ strong evidence for H_1, $30 < BF_{10} < 100$ very strong evidence for H_1, $BF_{10} > 100$ extreme evidence for H_1.

		**MI** $\Delta[HbO]$			
Null model (incl. subject)	0.500	0.611	1.572	1.000	
METHOD	0.500	0.389	0.636	0.636	0.352
		**MI** $\Delta[HbR]$			
Models	$P(M)$	$P(M|data)$	BF$_M$	BF$_{10}$	error %
Null model (incl. subject)	0.500	0.711	2.459	1.000	
METHOD	0.500	0.289	0.407	0.407	0.360

Table 19 Results of the Bayesian t-tests of the SDC CORMAT analysis of the $\Delta[HbR]$ data of SIM, ME LEFT, ME RIGHT and MI data. BF_{10} represent Bayes factors $\pm$ an error term in %. Bayesian t-tests were performed with the *BayesFactor* package[?] in R[?] for which the default prior odds is set at $P(M) = \frac{\sqrt{2}}{2}$. Corresponding SDC CORMATs are visualized in Figures 6 A, 8 A as well as 10 A.

SIM

	$\Delta[HbO]$				
	NO SAC	CAR	GCR	SSR	GLM ALL
NO SAC	-	-	-	-	-
CAR	$BF_{10} = 3.60 \cdot 10^{117} \pm 0\%$	-	-	-	-
GCR	$BF_{10} = 2.47 \cdot 10^{113} \pm 0\%$	$BF_{10} = 0.07 \pm 0.06\%$	-	-	-
SSR	$BF_{10} = 3.06 \cdot 10^{116} \pm 0\%$	$BF_{10} = 2.06 \cdot 10^{60} \pm 0\%$	$BF_{10} = 1.02 \cdot 10^{52} \pm 0\%$	-	-
GLM ALL	$BF_{10} = 2.29 \cdot 10^{115} \pm 0\%$	$BF_{10} = 7.73 \cdot 10^{35} \pm 0\%$	$BF_{10} = 4.81 \cdot 10^{28} \pm 0\%$	$BF_{10} = 5.92 \cdot 10^{25} \pm 0\%$	-
GLM BH	$BF_{10} = 4.17 \cdot 10^{110} \pm 0\%$	$BF_{10} = 9.07 \cdot 10^{70} \pm 0\%$	$BF_{10} = 6.72 \cdot 10^{56} \pm 0\%$	$BF_{10} = 100608 \pm 0\%$	$BF_{10} = 4.73 \cdot 10^{60} \pm 0\%$

ME LEFT

	$\Delta[HbO]$				
	NO SAC	CAR	GCR	SSR	GLM ALL
NO SAC	-	-	-	-	-
CAR	$BF_{10} = 1.80 \cdot 10^{113} \pm 0\%$	-	-	-	-
GCR	$BF_{10} = 1.96 \cdot 10^{112} \pm 0\%$	$BF_{10} = 0.08 \pm 0.05\%$	-	-	-
SSR	$BF_{10} = 5.70 \cdot 10^{114} \pm 0\%$	$BF_{10} = 4.07 \cdot 10^{64} \pm 0\%$	$BF_{10} = 5.03 \cdot 10^{60} \pm 0\%$	-	-
GLM ALL	$BF_{10} = 1.52 \cdot 10^{120} \pm 0\%$	$BF_{10} = 3.91 \cdot 10^{50} \pm 0\%$	$BF_{10} = 4.25 \cdot 10^{44} \pm 0\%$	$BF_{10} = 4.69 \cdot 10^{24} \pm 0\%$	-
GLM BH	$BF_{10} = 8.98 \cdot 10^{115} \pm 0\%$	$BF_{10} = 1.82 \cdot 10^{72} \pm 0\%$	$BF_{10} = 1.41 \cdot 10^{63} \pm 0\%$	$BF_{10} = 0.41 \pm 0.01\%$	$BF_{10} = 2.71 \cdot 10^{38} \pm 0\%$

ME RIGHT

	$\Delta[HbO]$				
	NO SAC	CAR	GCR	SSR	GLM ALL
NO SAC	-	-	-	-	-
CAR	$BF_{10} = 3.96 \cdot 10^{113} \pm 0\%$	-	-	-	-
GCR	$BF_{10} = 7.64 \cdot 10^{112} \pm 0\%$	$BF_{10} = 0.07 \pm 0.06\%$	-	-	-
SSR	$BF_{10} = 2.33 \cdot 10^{114} \pm 0\%$	$BF_{10} = 2.45 \cdot 10^{60} \pm 0\%$	$BF_{10} = 1.36 \cdot 10^{59} \pm 0\%$	-	-
GLM ALL	$BF_{10} = 1.00 \cdot 10^{121} \pm 0\%$	$BF_{10} = 3.18 \cdot 10^{46} \pm 0\%$	$BF_{10} = 1.02 \cdot 10^{41} \pm 0\%$	$BF_{10} = 2.15 \cdot 10^{32} \pm 0\%$	-
GLM BH	$BF_{10} = 1.85 \cdot 10^{114} \pm 0\%$	$BF_{10} = 1.09 \cdot 10^{70} \pm 0\%$	$BF_{10} = 2.21 \cdot 10^{62} \pm 0\%$	$BF_{10} = 42.26 \pm 0\%$	$BF_{10} = 3.45 \cdot 10^{54} \pm 0\%$

MI

	$\Delta[HbO]$				
	NO SAC	CAR	GCR	SSR	GLM ALL
NO SAC	-	-	-	-	-
CAR	$BF_{10} = 7.90 \cdot 10^{113} \pm 0\%$	-	-	-	-
GCR	$BF_{10} = 9.77 \cdot 10^{114} \pm 0\%$	$BF_{10} = 0.09 \pm 0.05\%$	-	-	-
SSR	$BF_{10} = 8.52 \cdot 10^{118} \pm 0\%$	$BF_{10} = 8.04 \cdot 10^{62} \pm 0\%$	$BF_{10} = 3.60 \cdot 10^{61} \pm 0\%$	-	-
GLM ALL	$BF_{10} = 1.62 \cdot 10^{123} \pm 0\%$	$BF_{10} = 2.74 \cdot 10^{50} \pm 0\%$	$BF_{10} = 2.22 \cdot 10^{45} \pm 0\%$	$BF_{10} = 1.70 \cdot 10^{34} \pm 0\%$	-
GLM BH	$BF_{10} = 8.77 \cdot 10^{115} \pm 0\%$	$BF_{10} = 2.42 \cdot 10^{73} \pm 0\%$	$BF_{10} = 2.60 \cdot 10^{67} \pm 0\%$	$BF_{10} = 5.78 \cdot 10^{5} \pm 0\%$	$BF_{10} = 1.18 \cdot 10^{64} \pm 0\%$

Table 20 Results of the Bayesian t-tests of the SDC CORMAT analysis of the $\Delta[HbR]$ data of SIM, ME LEFT, ME RIGHT and MI data. BF_{10} represent Bayes factors $\pm$ an error term in %. Bayesian t-tests were performed with the *BayesFactor* package[?] in R[?] for which the default prior odds is set at $P(M) = \frac{\sqrt{2}}{2}$. Corresponding SDC CORMATs are visualized in Figures 6 A, 8 A as well as 10 A.

SIM — $\Delta[HbR]$

	NO SAC	CAR	GCR	SSR	GLM ALL
NO SAC	-	-	-	-	-
CAR	$BF_{10} = 2.63 \cdot 10^{91} \pm 0\%$	-	-	-	-
GCR	$BF_{10} = 1.20 \cdot 10^{80} \pm 0\%$	$BF_{10} = 0.08 \pm 0.05\%$	-	-	-
SSR	$BF_{10} = 5.19 \cdot 10^{74} \pm 0\%$	$BF_{10} = 3.25 \cdot 10^{57} \pm 0\%$	$BF_{10} = 5.08 \cdot 10^{49} \pm 0\%$	-	-
GLM ALL	$BF_{10} = 1.04 \cdot 10^{82} \pm 0\%$	$BF_{10} = 2.03 \cdot 10^{37} \pm 0\%$	$BF_{10} = 1.84 \cdot 10^{30} \pm 0\%$	$BF_{10} = 2.81 \cdot 10^{27} \pm 0\%$	-
GLM BH	$BF_{10} = 2.41 \cdot 10^{53} \pm 0\%$	$BF_{10} = 5.28 \cdot 10^{20} \pm 0\%$	$BF_{10} = 4.19 \cdot 10^{17} \pm 0\%$	$BF_{10} = 1.26 \cdot 10^{13} \pm 0\%$	$BF_{10} = 0.20 \pm 0.02\%$

ME LEFT — $\Delta[HbR]$

	NO SAC	CAR	GCR	SSR	GLM ALL
NO SAC	-	-	-	-	-
CAR	$BF_{10} = 1.92 \cdot 10^{63} \pm 0\%$	-	-	-	-
GCR	$BF_{10} = 4.29 \cdot 10^{64} \pm 0\%$	$BF_{10} = 0.07 \pm 0.06\%$	-	-	-
SSR	$BF_{10} = 2.77 \cdot 10^{24} \pm 0\%$	$BF_{10} = 7.85 \cdot 10^{46} \pm 0\%$	$BF_{10} = 5.81 \cdot 10^{43} \pm 0\%$	-	-
GLM ALL	$BF_{10} = 9.94 \cdot 10^{36} \pm 0\%$	$BF_{10} = 4.51 \cdot 10^{38} \pm 0\%$	$BF_{10} = 3.62 \cdot 10^{35} \pm 0\%$	$BF_{10} = 4.70 \cdot 10^{22} \pm 0\%$	-
GLM BH	$BF_{10} = 5.11 \cdot 10^{15} \pm 0\%$	$BF_{10} = 2.27 \cdot 10^{54} \pm 0\%$	$BF_{10} = 2.74 \cdot 10^{50} \pm 0\%$	$BF_{10} = 5.09 \cdot 10^{8} \pm 0\%$	$BF_{10} = 2.21 \cdot 10^{63} \pm 0\%$

ME RIGHT — $\Delta[HbR]$

	NO SAC	CAR	GCR	SSR	GLM ALL
NO SAC	-	-	-	-	-
CAR	$BF_{10} = 8.41 \cdot 10^{85} \pm 0\%$	-	-	-	-
GCR	$BF_{10} = 1.75 \cdot 10^{79} \pm 0\%$	$BF_{10} = 0.07 \pm 0.06\%$	-	-	-
SSR	$BF_{10} = 2.64 \cdot 10^{74} \pm 0\%$	$BF_{10} = 2.94 \cdot 10^{57} \pm 0\%$	$BF_{10} = 1.06 \cdot 10^{53} \pm 0\%$	-	-
GLM ALL	$BF_{10} = 4.36 \cdot 10^{92} \pm 0\%$	$BF_{10} = 9.68 \cdot 10^{43} \pm 0\%$	$BF_{10} = 3.42 \cdot 10^{39} \pm 0\%$	$BF_{10} = 1.44 \cdot 10^{43} \pm 0\%$	-
GLM BH	$BF_{10} = 9.67 \cdot 10^{75} \pm 0\%$	$BF_{10} = 3.06 \cdot 10^{61} \pm 0\%$	$BF_{10} = 4.61 \cdot 10^{55} \pm 0\%$	$BF_{10} = 0.39 \pm 0.01\%$	$BF_{10} = 1.02 \cdot 10^{70} \pm 0\%$

MI — $\Delta[HbR]$

	NO SAC	CAR	GCR	SSR	GLM ALL
NO SAC	-	-	-	-	-
CAR	$BF_{10} = 3.21 \cdot 10^{74} \pm 0\%$	-	-	-	-
GCR	$BF_{10} = 2.44 \cdot 10^{69} \pm 0\%$	$BF_{10} = 0.07 \pm 0.06\%$	-	-	-
SSR	$BF_{10} = 3.05 \cdot 10^{72} \pm 0\%$	$BF_{10} = 6.34 \cdot 10^{41} \pm 0\%$	$BF_{10} = 8.69 \cdot 10^{38} \pm 0\%$	-	-
GLM ALL	$BF_{10} = 4.34 \cdot 10^{91} \pm 0\%$	$BF_{10} = 2.28 \cdot 10^{33} \pm 0\%$	$BF_{10} = 2.21 \cdot 10^{30} \pm 0\%$	$BF_{10} = 1.39 \cdot 10^{33} \pm 0\%$	-
GLM BH	$BF_{10} = 9.59 \cdot 10^{74} \pm 0\%$	$BF_{10} = 3.45 \cdot 10^{51} \pm 0\%$	$BF_{10} = 1.03 \cdot 10^{47} \pm 0\%$	$BF_{10} = 2.85 \cdot 10^{5} \pm 0\%$	$BF_{10} = 1.56 \cdot 10^{71} \pm 0\%$

Table 21 Results of the Bayesian t-tests of the BETA MAPS analysis of the SIM $\Delta[HbO]$ data. Mean $\pm$ SEM represent mean beta values of the respective channel and its standard error of the mean across participants, BF_{10} represent Bayes factors. Bayesian t-tests were performed with the *BayesFactor* package in R for which the default prior odds is set at $P(M) = \frac{\sqrt{2}}{2}$. Corresponding BETA MAPS are visualized in Figure 6 B of the main document.

			**SIM**			
Channel (Channel Frequency after Pruning)	**NO SAC**	**CAR**	**GCR**	**SSR**	**GLM ALL**	**GLM BH**
1 (20)	$\overline{\beta} = 0.28 \pm 0.81$<br>$BF_{10} = 0.25$	$\overline{\beta} = -0.75 \pm 0.38$<br>$BF_{10} = 1.18$	$\overline{\beta} = -0.10 \pm 0.22$<br>$BF_{10} = 0.25$	$\overline{\beta} = 0.07 \pm 0.34$<br>$BF_{10} = 0.24$	$\overline{\beta} = -0.05 \pm 0.15$<br>$BF_{10} = 0.25$	$\overline{\beta} = 0.00 \pm 0.32$<br>$BF_{10} = 0.23$
2 (19)	$\overline{\beta} = -0.35 \pm 0.92$<br>$BF_{10} = 0.25$	$\overline{\beta} = -1.37 \pm 0.38$<br>$\mathbf{BF_{10} = 21.77}$	$\overline{\beta} = -1.25 \pm 0.25$<br>$\mathbf{BF_{10} = 124.96}$	$\overline{\beta} = -0.43 \pm 0.41$<br>$BF_{10} = 0.38$	$\overline{\beta} = -0.04 \pm 0.23$<br>$BF_{10} = 0.24$	$\overline{\beta} = -0.31 \pm 0.47$<br>$BF_{10} = 0.29$
4 (19)	$\overline{\beta} = -0.47 \pm 0.82$<br>$BF_{10} = 0.28$	$\overline{\beta} = -1.54 \pm 0.39$<br>$\mathbf{BF_{10} = 36.18}$	$\overline{\beta} = -0.95 \pm 0.25$<br>$\mathbf{BF_{10} = 28.35}$	$\overline{\beta} = -0.15 \pm 0.44$<br>$BF_{10} = 0.25$	$\overline{\beta} = -0.09 \pm 0.16$<br>$BF_{10} = 0.27$	$\overline{\beta} = -0.53 \pm 0.31$<br>$BF_{10} = 0.84$
6 (23)	$\overline{\beta} = 8.16 \pm 0.87$<br>$\mathbf{BF_{10} = 3.42 \cdot 10^6}$	$\overline{\beta} = 7.22 \pm 0.43$<br>$\mathbf{BF_{10} = 1.38 \cdot 10^{11}}$	$\overline{\beta} = 5.70 \pm 0.32$<br>$\mathbf{BF_{10} = 4.08 \cdot 10^{11}}$	$\overline{\beta} = 7.96 \pm 0.52$<br>$\mathbf{BF_{10} = 1.93 \cdot 10^{10}}$	$\overline{\beta} = 7.30 \pm 0.38$<br>$\mathbf{BF_{10} = 1.66 \cdot 10^{12}}$	$\overline{\beta} = 8.61 \pm 0.49$<br>$\mathbf{BF_{10} = 2.54 \cdot 10^{11}}$
7 (18)	$\overline{\beta} = 0.00 \pm 0.94$<br>$BF_{10} = 0.24$	$\overline{\beta} = -0.72 \pm 0.32$<br>$BF_{10} = 1.83$	$\overline{\beta} = -1.94 \pm 0.29$<br>$\mathbf{BF_{10} = 5120.13}$	$\overline{\beta} = 0.34 \pm 0.43$<br>$BF_{10} = 0.32$	$\overline{\beta} = 0.11 \pm 0.13$<br>$BF_{10} = 0.33$	$\overline{\beta} = 0.09 \pm 0.49$<br>$BF_{10} = 0.25$
8 (23)	$\overline{\beta} = 4.17 \pm 0.87$<br>$\mathbf{BF_{10} = 303.90}$	$\overline{\beta} = 3.12 \pm 0.32$<br>$\mathbf{BF_{10} = 4.58 \cdot 10^6}$	$\overline{\beta} = 1.78 \pm 0.26$<br>$\mathbf{BF_{10} = 2.88 \cdot 10^4}$	$\overline{\beta} = 3.95 \pm 0.36$<br>$\mathbf{BF_{10} = 4.47 \cdot 10^7}$	$\overline{\beta} = 3.00 \pm 0.30$<br>$\mathbf{BF_{10} = 1.06 \cdot 10^7}$	$\overline{\beta} = 4.03 \pm 0.41$<br>$\mathbf{BF_{10} = 6.72 \cdot 10^6}$
10 (13)	$\overline{\beta} = -0.59 \pm 1.08$<br>$BF_{10} = 0.32$	$\overline{\beta} = -1.34 \pm 0.57$<br>$BF_{10} = 2.12$	$\overline{\beta} = -0.63 \pm 0.39$<br>$BF_{10} = 0.476$	$\overline{\beta} = 0.44 \pm 0.56$<br>$BF_{10} = 0.36$	$\overline{\beta} = 0.10 \pm 0.24$<br>$BF_{10} = 0.30$	$\overline{\beta} = -0.08 \pm 0.40$<br>$BF_{10} = 0.28$
11 (20)	$\overline{\beta} = 0.05 \pm 0.74$<br>$BF_{10} = 0.23$	$\overline{\beta} = -1.00 \pm 0.26$<br>$\mathbf{BF_{10} = 38.05}$	$\overline{\beta} = -0.53 \pm 0.24$<br>$BF_{10} = 1.67$	$\overline{\beta} = 0.58 \pm 0.31$<br>$BF_{10} = 0.99$	$\overline{\beta} = 0.09 \pm 0.14$<br>$BF_{10} = 0.28$	$\overline{\beta} = 0.25 \pm 0.24$<br>$BF_{10} = 0.37$
12 (18)	$\overline{\beta} = 0.59 \pm 0.95$<br>$BF_{10} = 0.29$	$\overline{\beta} = -0.56 \pm 0.22$<br>$BF_{10} = 2.97$	$\overline{\beta} = 0.05 \pm 0.17$<br>$BF_{10} = 0.25$	$\overline{\beta} = 0.97 \pm 0.49$<br>$BF_{10} = 1.20$	$\overline{\beta} = 0.49 \pm 0.21$<br>$BF_{10} = 1.91$	$\overline{\beta} = 0.75 \pm 0.36$<br>$BF_{10} = 1.42$
13 (17)	$\overline{\beta} = -0.65 \pm 0.77$<br>$BF_{10} = 0.34$	$\overline{\beta} = -0.87 \pm 0.55$<br>$BF_{10} = 0.72$	$\overline{\beta} = -0.33 \pm 0.42$<br>$BF_{10} = 0.32$	$\overline{\beta} = 0.02 \pm 0.53$<br>$BF_{10} = 0.25$	$\overline{\beta} = 0.10 \pm 0.14$<br>$BF_{10} = 0.31$	$\overline{\beta} = 0.24 \pm 0.50$<br>$BF_{10} = 0.28$
15 (23)	$\overline{\beta} = 1.01 \pm 0.80$<br>$BF_{10} = 0.44$	$\overline{\beta} = -0.16 \pm 0.26$<br>$BF_{10} = 0.26$	$\overline{\beta} = 0.53 \pm 0.20$<br>$\mathbf{BF_{10} = 4.22}$	$\overline{\beta} = 0.62 \pm 0.29$<br>$BF_{10} = 1.49$	$\overline{\beta} = 0.51 \pm 0.16$<br>$\mathbf{BF_{10} = 9.35}$	$\overline{\beta} = 0.79 \pm 0.34$<br>$BF_{10} = 1.92$
17 (17)	$\overline{\beta} = 0.36 \pm 1.02$<br>$BF_{10} = 0.26$	$\overline{\beta} = -0.79 \pm 0.44$<br>$BF_{10} = 0.94$	$\overline{\beta} = -1.60 \pm 0.43$<br>$\mathbf{BF_{10} = 21.56}$	$\overline{\beta} = 0.52 \pm 0.34$<br>$BF_{10} = 0.67$	$\overline{\beta} = 0.15 \pm 0.21$<br>$BF_{10} = 0.31$	$\overline{\beta} = 0.60 \pm 0.37$<br>$BF_{10} = 0.73$
18 (22)	$\overline{\beta} = -1.07 \pm 0.0.97$<br>$BF_{10} = 0.38$	$\overline{\beta} = -1.75 \pm 0.32$<br>$\mathbf{BF_{10} = 1321.84}$	$\overline{\beta} = -2.50 \pm 0.24$<br>$\mathbf{BF_{10} = 9.20 \cdot 10^6}$	$\overline{\beta} = 0.17 \pm 0.38$<br>$BF_{10} = 0.24$	$\overline{\beta} = 0.10 \pm 0.28$<br>$BF_{10} = 0.28$	$\overline{\beta} = -0.39 \pm 0.39$<br>$BF_{10} = 0.35$
19 (20)	$\overline{\beta} = -1.05 \pm 0.0.95$<br>$BF_{10} = 0.40$	$\overline{\beta} = -1.83 \pm 0.51$<br>$\mathbf{BF_{10} = 20.41}$	$\overline{\beta} = -1.21 \pm 0.30$<br>$\mathbf{BF_{10} = 51.04}$	$\overline{\beta} = -0.07 \pm 0.34$<br>$BF_{10} = 0.24$	$\overline{\beta} = 0.30 \pm 0.11$<br>$\mathbf{BF_{10} = 3.72}$	$\overline{\beta} = -0.19 \pm 0.26$<br>$BF_{10} = 0.29$
21 (23)	$\overline{\beta} = 0.35 \pm 0.79$<br>$BF_{10} = 0.24$	$\overline{\beta} = 0.06 \pm 0.37$<br>$BF_{10} = 0.22$	$\overline{\beta} = 0.49 \pm 0.31$<br>$BF_{10} = 0.62$	$\overline{\beta} = 0.38 \pm 0.33$<br>$BF_{10} = 0.40$	$\overline{\beta} = 0.18 \pm 0.21$<br>$BF_{10} = 0.31$	$\overline{\beta} = 0.34 \pm 0.34$<br>$BF_{10} = 0.35$
22 (21)	$\overline{\beta} = -0.29 \pm 0.92$<br>$BF_{10} = 0.24$	$\overline{\beta} = -0.86 \pm 0.40$<br>$BF_{10} = 1.43$	$\overline{\beta} = -0.24 \pm 0.23$<br>$BF_{10} = 0.37$	$\overline{\beta} = 0.53 \pm 0.43$<br>$BF_{10} = 0.44$	$\overline{\beta} = 0.16 \pm 0.21$<br>$BF_{10} = 0.29$	$\overline{\beta} = 0.07 \pm 0.23$<br>$BF_{10} = 0.24$

Table 22 Results of the Bayesian t-tests of the BETA MAPS analysis of the SIM $\Delta[HbR]$ data. Mean $\pm$ SEM represent mean beta values of the respective channel and its standard error of the mean across participants, BF_{10} represent Bayes factors. Bayesian t-tests were performed with the *BayesFactor* package in R for which the default prior odds is set at $P(M) = \frac{\sqrt{2}}{2}$. Corresponding BETA MAPS are visualized in Figure 6 B of the main document.

	SIM					
Channel (Channel Frequency after Pruning)	**NO SAC**	**CAR**	**GCR**	**SSR**	**GLM ALL**	**GLM BH**
1 (20)	$\overline{\beta} = 0.02 \pm 0.10$<br>$BF_{10} = 0.24$	$\overline{\beta} = 0.27 \pm 0.08$<br>$\mathbf{BF_{10} = 19.09}$	$\overline{\beta} = 0.09 \pm 0.06$<br>$BF_{10} = 0.58$	$\overline{\beta} = 0.02 \pm 0.09$<br>$BF_{10} = 0.24$	$\overline{\beta} = -0.07 \pm 0.05$<br>$BF_{10} = 0.57$	$\overline{\beta} = 0.00 \pm 0.05$<br>$BF_{10} = 0.23$
2 (19)	$\overline{\beta} = -0.12 \pm 0.30$<br>$BF_{10} = 0.25$	$\overline{\beta} = 0.12 \pm 0.29$<br>$BF_{10} = 0.26$	$\overline{\beta} = 0.25 \pm 0.18$<br>$BF_{10} = 0.51$	$\overline{\beta} = -0.13 \pm 0.32$<br>$BF_{10} = 0.26$	$\overline{\beta} = -0.12 \pm 0.14$<br>$BF_{10} = 0.33$	$\overline{\beta} = -0.09 \pm 0.31$<br>$BF_{10} = 0.25$
4 (19)	$\overline{\beta} = 0.13 \pm 0.13$<br>$BF_{10} = 0.37$	$\overline{\beta} = 0.40 \pm 0.08$<br>$\mathbf{BF_{10} = 276.64}$	$\overline{\beta} = 0.20 \pm 0.05$<br>$\mathbf{BF_{10} = 35.93}$	$\overline{\beta} = 0.12 \pm 0.09$<br>$BF_{10} = 0.56$	$\overline{\beta} = -0.04 \pm 0.05$<br>$BF_{10} = 0.34$	$\overline{\beta} = 0.03 \pm 0.06$<br>$BF_{10} = 0.28$
6 (23)	$\overline{\beta} = -2.47 \pm 0.15$<br>$\mathbf{BF_{10} = 6.70 \cdot 10^{10}}$	$\overline{\beta} = -2.25 \pm 0.13$<br>$\mathbf{BF_{10} = 3.36 \cdot 10^{11}}$	$\overline{\beta} = -1.73 \pm 0.12$<br>$\mathbf{BF_{10} = 4.63 \cdot 10^{9}}$	$\overline{\beta} = -2.46 \pm 0.15$<br>$\mathbf{BF_{10} = 9.50 \cdot 10^{10}}$	$\overline{\beta} = -2.15 \pm 0.15$<br>$\mathbf{BF_{10} = 7.19 \cdot 10^{9}}$	$\overline{\beta} = -2.57 \pm 0.15$<br>$\mathbf{BF_{10} = 2.02 \cdot 10^{11}}$
7 (18)	$\overline{\beta} = 0.00 \pm 0.18$<br>$BF_{10} = 0.24$	$\overline{\beta} = 0.24 \pm 0.11$<br>$BF_{10} = 1.84$	$\overline{\beta} = 0.61 \pm 0.08$<br>$\mathbf{BF_{10} = 1.47 \cdot 10^{4}}$	$\overline{\beta} = 0.04 \pm 0.18$<br>$BF_{10} = 0.25$	$\overline{\beta} = -0.10 \pm 0.12$<br>$BF_{10} = 0.33$	$\overline{\beta} = -0.04 \pm 0.16$<br>$BF_{10} = 0.25$
8 (23)	$\overline{\beta} = -1.16 \pm 0.14$<br>$\mathbf{BF_{10} = 4.96 \cdot 10^{5}}$	$\overline{\beta} = -10.97 \pm 0.13$<br>$\mathbf{BF_{10} = 5.88 \cdot 10^{4}}$	$\overline{\beta} = -0.51 \pm 0.10$<br>$\mathbf{BF_{10} = 415.47}$	$\overline{\beta} = -1.36 \pm 0.15$<br>$\mathbf{BF_{10} = 1.25 \cdot 10^{6}}$	$\overline{\beta} = -1.00 \pm 0.10$<br>$\mathbf{BF_{10} = 4.74 \cdot 10^{6}}$	$\overline{\beta} = -1.30 \pm 0.12$<br>$\mathbf{BF_{10} = 2.04 \cdot 10^{7}}$
10 (13)	$\overline{\beta} = 0.25 \pm 0.14$<br>$BF_{10} = 1.04$	$\overline{\beta} = 0.48 \pm 0.14$<br>$\mathbf{BF_{10} = 9.25}$	$\overline{\beta} = 0.17 \pm 0.13$<br>$BF_{10} = 0.55$	$\overline{\beta} = 0.27 \pm 0.15$<br>$BF_{10} = 1.04$	$\overline{\beta} = 0.11 \pm 0.09$<br>$BF_{10} = 0.56$	$\overline{\beta} = 0.22 \pm 0.11$<br>$BF_{10} = 1.23$
11 (20)	$\overline{\beta} = 0.18 \pm 0.15$<br>$BF_{10} = 0.43$	$\overline{\beta} = 0.41 \pm 0.07$<br>$\mathbf{BF_{10} = 2428.17}$	$\overline{\beta} = 0.16 \pm 0.06$<br>$\mathbf{BF_{10} = 3.12}$	$\overline{\beta} = 0.14 \pm 0.10$<br>$BF_{10} = 0.52$	$\overline{\beta} = -0.06 \pm 0.06$<br>$BF_{10} = 0.37$	$\overline{\beta} = 0.05 \pm 0.09$<br>$BF_{10} = 0.27$
12 (18)	$\overline{\beta} = -0.18 \pm 0.16$<br>$BF_{10} = 0.42$	$\overline{\beta} = 0.07 \pm 0.08$<br>$BF_{10} = 0.33$	$\overline{\beta} = -0.18 \pm 0.06$<br>$\mathbf{BF_{10} = 10.78}$	$\overline{\beta} = -0.34 \pm 0.12$<br>$\mathbf{BF_{10} = 5.04}$	$\overline{\beta} = -0.15 \pm 0.07$<br>$BF_{10} = 1.35$	$\overline{\beta} = -0.19 \pm 0.09$<br>$BF_{10} = 1.51$
13 (17)	$\overline{\beta} = 0.17 \pm 0.15$<br>$BF_{10} = 0.42$	$\overline{\beta} = 0.43 \pm 0.12$<br>$\mathbf{BF_{10} = 19.55}$	$\overline{\beta} = 0.17 \pm 0.10$<br>$BF_{10} = 0.83$	$\overline{\beta} = 0.24 \pm 0.23$<br>$BF_{10} = 0.39$	$\overline{\beta} = 0.03 \pm 0.05$<br>$BF_{10} = 0.28$	$\overline{\beta} = 0.29 \pm 0.21$<br>$BF_{10} = 0.53$
15 (23)	$\overline{\beta} = 0.10 \pm 0.12$<br>$BF_{10} = 0.29$	$\overline{\beta} = 0.31 \pm 0.09$<br>$\mathbf{BF_{10} = 11.06}$	$\overline{\beta} = 0.05 \pm 0.07$<br>$BF_{10} = 0.28$	$\overline{\beta} = 0.10 \pm 0.10$<br>$BF_{10} = 0.34$	$\overline{\beta} = -0.04 \pm 0.07$<br>$BF_{10} = 0.26$	$\overline{\beta} = -0.05 \pm 0.10$<br>$BF_{10} = 0.25$
17 (17)	$\overline{\beta} = 0.09 \pm 0.16$<br>$BF_{10} = 0.29$	$\overline{\beta} = 0.22 \pm 0.08$<br>$BF_{10} = 3.00$	$\overline{\beta} = 0.53 \pm 0.08$<br>$\mathbf{BF_{10} = 3126.04}$	$\overline{\beta} = 0.11 \pm 0.13$<br>$BF_{10} = 0.35$	$\overline{\beta} = 0.02 \pm 0.07$<br>$BF_{10} = 0.25$	$\overline{\beta} = 0.06 \pm 0.08$<br>$BF_{10} = 0.31$
18 (22)	$\overline{\beta} = -0.05 \pm 0.13$<br>$BF_{10} = 0.24$	$\overline{\beta} = 0.21 \pm 0.09$<br>$BF_{10} = 1.88$	$\overline{\beta} = 0.55 \pm 0.06$<br>$\mathbf{BF_{10} = 1.09 \cdot 10^{6}}$	$\overline{\beta} = -0.06 \pm 0.13$<br>$BF_{10} = 0.24$	$\overline{\beta} = -0.05 \pm 0.06$<br>$BF_{10} = 0.30$	$\overline{\beta} = -0.04 \pm 0.08$<br>$BF_{10} = 0.25$
19 (20)	$\overline{\beta} = 0.20 \pm 0.12$<br>$BF_{10} = 0.72$	$\overline{\beta} = 0.50 \pm 0.11$<br>$\mathbf{BF_{10} = 188.58}$	$\overline{\beta} = 0.33 \pm 0.08$<br>$\mathbf{BF_{10} = 55.39}$	$\overline{\beta} = 0.15 \pm 0.11$<br>$BF_{10} = 0.51$	$\overline{\beta} = -0.01 \pm 0.07$<br>$BF_{10} = 0.23$	$\overline{\beta} = 0.10 \pm 0.11$<br>$BF_{10} = 0.34$
21 (23)	$\overline{\beta} = -0.09 \pm 0.13$<br>$BF_{10} = 0.27$	$\overline{\beta} = 0.21 \pm 0.07$<br>$\mathbf{BF_{10} = 5.74}$	$\overline{\beta} = -0.10 \pm 0.06$<br>$BF_{10} = 0.63$	$\overline{\beta} = -0.11 \pm 0.11$<br>$BF_{10} = 0.32$	$\overline{\beta} = -0.08 \pm 0.07$<br>$BF_{10} = 0.39$	$\overline{\beta} = -0.19 \pm 0.10$<br>$BF_{10} = 1.10$
22 (21)	$\overline{\beta} = 0.05 \pm 0.14$<br>$BF_{10} = 0.24$	$\overline{\beta} = 0.23 \pm 0.07$<br>$\mathbf{BF_{10} = 8.18}$	$\overline{\beta} = -0.01 \pm 0.05$<br>$BF_{10} = 0.23$	$\overline{\beta} = 0.06 \pm 0.11$<br>$BF_{10} = 0.26$	$\overline{\beta} = 0.09 \pm 0.08$<br>$BF_{10} = 0.39$	$\overline{\beta} = 0.07 \pm 0.11$<br>$BF_{10} = 0.27$

Table 23 Results of the Bayesian t-tests of the BETA MAPS analysis of the ME LEFT and ME RIGHT $\Delta[HbO]$ data. Mean ± SEM represent mean beta values of the respective channel and its standard error of the mean across participants, BF_{10} represent Bayes factors. Bayesian t-tests were performed with the *BayesFactor* package in R for which the default prior odds is set at $P(M) = \frac{\sqrt{2}}{2}$. Corresponding BETA MAPS are visualized in Figure 8 B of the main document.

ME LEFT

Channel (Channel Frequency after Pruning)	**NO SAC**	**CAR**	**GCR**	**SSR**	**GLM ALL**	**GLM BH**
1 (23)	$\beta = 1.91 \pm 0.67$<br>$\mathbf{BF_{10} = 5.04}$	$\beta = -2.02 \pm 0.41$<br>$\mathbf{BF_{10} = 376.91}$	$\beta = -1.39 \pm 0.30$<br>$\mathbf{BF_{10} = 249.65}$	$\beta = 0.56 \pm 0.24$<br>$BF_{10} = 2.16$	$\beta = -0.21 \pm 0.17$<br>$BF_{10} = 0.43$	$\beta = -0.29 \pm 0.24$<br>$BF_{10} = 0.42$
2 (16)	$\beta = 3.12 \pm 0.93$<br>$\mathbf{BF_{10} = 10.92}$	$\beta = -0.76 \pm 0.52$<br>$BF_{10} = 0.62$	$\beta = -0.39 \pm 0.43$<br>$BF_{10} = 0.36$	$\beta = 1.19 \pm 0.47$<br>$BF_{10} = 2.64$	$\beta = 0.56 \pm 0.33$<br>$BF_{10} = 0.81$	$\beta = 0.48 \pm 0.46$<br>$BF_{10} = 0.41$
4 (17)	$\beta = 3.40 \pm 0.91$<br>$\mathbf{BF_{10} = 22.83}$	$\beta = -0.80 \pm 0.24$<br>$\mathbf{BF_{10} = 9.82}$	$\beta = -0.29 \pm 0.17$<br>$BF_{10} = 0.89$	$\beta = 0.73 \pm 0.21$<br>$\mathbf{BF_{10} = 15.76}$	$\beta = 0.40 \pm 0.13$<br>$\mathbf{BF_{10} = 7.03}$	$\beta = 0.39 \pm 0.17$<br>$BF_{10} = 1.91$
6 (24)	$\beta = 3.01 \pm 0.71$<br>$\mathbf{BF_{10} = 96.51}$	$\beta = -0.71 \pm 0.42$<br>$BF_{10} = 0.75$	$\beta = 0.01 \pm 0.11$<br>$BF_{10} = 0.22$	$\beta = -0.69 \pm 0.15$<br>$\mathbf{BF_{10} = 226.55}$	$\beta = -0.46 \pm 0.10$<br>$\mathbf{BF_{10} = 221.94}$	$\beta = -0.58 \pm 0.13$<br>$\mathbf{BF_{10} = 159.51}$
7 (19)	$\beta = 5.10 \pm 0.86$<br>$\mathbf{BF_{10} = 1752.49}$	$\beta = 0.62 \pm 0.34$<br>$BF_{10} = 0.95$	$\beta = 0.78 \pm 0.27$<br>$\mathbf{BF_{10} = 5.35}$	$\beta = 2.27 \pm 0.33$<br>$\mathbf{BF_{10} = 10.77 \cdot 10^3}$	$\beta = 1.55 \pm 0.29$<br>$\mathbf{BF_{10} = 561.50}$	$\beta = 2.01 \pm 0.40$<br>$\mathbf{BF_{10} = 318.17}$
8 (23)	$\beta = 4.34 \pm 0.94$<br>$\mathbf{BF_{10} = 200.72}$	$\beta = 0.24 \pm 0.40$<br>$BF_{10} = 0.26$	$\beta = 0.28 \pm 0.36$<br>$BF_{10} = 0.29$	$\beta = 1.36 \pm 0.38$<br>$\mathbf{BF_{10} = 20.57}$	$\beta = 0.97 \pm 0.35$<br>$\mathbf{BF_{10} = 4.46}$	$\beta = 1.45 \pm 0.50$<br>$\mathbf{BF_{10} = 5.63}$
10 (17)	$\beta = 3.88 \pm 0.83$<br>$\mathbf{BF_{10} = 131.36}$	$\beta = -0.07 \pm 0.43$<br>$BF_{10} = 0.25$	$\beta = 0.19 \pm 0.33$<br>$BF_{10} = 0.29$	$\beta = 1.51 \pm 0.40$<br>$\mathbf{BF_{10} = 26.13}$	$\beta = 1.05 \pm 0.35$<br>$\mathbf{BF_{10} = 6.57}$	$\beta = 0.98 \pm 0.42$<br>$BF_{10} = 2.03$
11 (20)	$\beta = 4.20 \pm 0.92$<br>$\mathbf{BF_{10} = 149.48}$	$\beta = 0.36 \pm 0.23$<br>$BF_{10} = 0.64$	$\beta = 0.81 \pm 0.30$<br>$\mathbf{BF_{10} = 4.35}$	$\beta = 1.25 \pm 0.26$<br>$\mathbf{BF_{10} = 207.60}$	$\beta = 0.77 \pm 0.18$<br>$\mathbf{BF_{10} = 93.81}$	$\beta = 1.02 \pm 0.23$<br>$\mathbf{BF_{10} = 111.29}$
12 (19)	$\beta = 4.31 \pm 0.91$<br>$\mathbf{BF_{10} = 175.62}$	$\beta = 0.66 \pm 0.35$<br>$BF_{10} = 1.02$	$\beta = 0.22 \pm 0.29$<br>$BF_{10} = 0.31$	$\beta = 1.75 \pm 0.50$<br>$\mathbf{BF_{10} = 17.17}$	$\beta = 1.45 \pm 0.30$<br>$\mathbf{BF_{10} = 238.65}$	$\beta = 1.46 \pm 0.38$<br>$\mathbf{BF_{10} = 29.79}$
13 (17)	$\beta = 4.13 \pm 1.03$<br>$\mathbf{BF_{10} = 37.12}$	$\beta = 0.42 \pm 0.29$<br>$BF_{10} = 0.60$	$\beta = 0.02 \pm 0.28$<br>$BF_{10} = 0.25$	$\beta = 1.50 \pm 0.44$<br>$\mathbf{BF_{10} = 12.86}$	$\beta = 1.01 \pm 0.23$<br>$\mathbf{BF_{10} = 82.38}$	$\beta = 1.40 \pm 0.24$<br>$\mathbf{BF_{10} = 1150.48}$
15 (23)	$\beta = 4.01 \pm 0.74$<br>$\mathbf{BF_{10} = 1214.67}$	$\beta = 0.14 \pm 0.37$<br>$BF_{10} = 0.23$	$\beta = -0.42 \pm 0.27$<br>$BF_{10} = 0.62$	$\beta = 3.17 \pm 0.54$<br>$\mathbf{BF_{10} = 3.03 \cdot 10^3}$	$\beta = 1.75 \pm 0.37$<br>$\mathbf{BF_{10} = 241.18}$	$\beta = 2.30 \pm 0.52$<br>$\mathbf{BF_{10} = 131.99}$
17 (20)	$\beta = 5.15 \pm 1.05$<br>$\mathbf{BF_{10} = 281.44}$	$\beta = 0.83 \pm 0.59$<br>$BF_{10} = 0.55$	$\beta = 0.77 \pm 0.53$<br>$BF_{10} = 0.58$	$\beta = 2.07 \pm 0.60$<br>$\mathbf{BF_{10} = 15.86}$	$\beta = 1.08 \pm 0.44$<br>$BF_{10} = 2.45$	$\beta = 1.57 \pm 0.47$<br>$\mathbf{BF_{10} = 12.27}$
18 (21)	$\beta = 3.70 \pm 0.94$<br>$\mathbf{BF_{10} = 44.28}$	$\beta = -0.44 \pm 0.44$<br>$BF_{10} = 0.35$	$\beta = -0.44 \pm 0.34$<br>$BF_{10} = 0.47$	$\beta = 0.66 \pm 0.27$<br>$BF_{10} = 2.28$	$\beta = 0.00 \pm 0.17$<br>$BF_{10} = 0.23$	$\beta = 0.33 \pm 0.27$<br>$BF_{10} = 0.44$
19 (20)	$\beta = 3.04 \pm 1.03$<br>$\mathbf{BF_{10} = 6.00}$	$\beta = -1.07 \pm 0.48$<br>$BF_{10} = 1.68$	$\beta = -0.95 \pm 0.31$<br>$\mathbf{BF_{10} = 8.15}$	$\beta = 0.14 \pm 0.24$<br>$BF_{10} = 0.27$	$\beta = -0.04 \pm 0.18$<br>$BF_{10} = 0.24$	$\beta = 0.03 \pm 0.17$<br>$BF_{10} = 0.24$
21 (23)	$\beta = 5.74 \pm 1.14$<br>$\mathbf{BF_{10} = 503.95}$	$\beta = 1.86 \pm 0.59$<br>$\mathbf{BF_{10} = 9.27}$	$\beta = 1.26 \pm 0.40$<br>$\mathbf{BF_{10} = 10.14}$	$\beta = 3.28 \pm 0.72$<br>$\mathbf{BF_{10} = 188.73}$	$\beta = 2.79 \pm 0.58$<br>$\mathbf{BF_{10} = 335.61}$	$\beta = 3.06 \pm 0.69$<br>$\mathbf{BF_{10} = 150.44}$
22 (21)	$\beta = 4.53 \pm 1.00$<br>$\mathbf{BF_{10} = 143.36}$	$\beta = 0.39 \pm 0.51$<br>$BF_{10} = 0.29$	$\beta = -0.01 \pm 0.38$<br>$BF_{10} = 0.23$	$\beta = 1.68 \pm 0.44$<br>$\mathbf{BF_{10} = 32.79}$	$\beta = 1.31 \pm 0.39$<br>$\mathbf{BF_{10} = 12.98}$	$\beta = 1.23 \pm 0.53$<br>$BF_{10} = 2.01$

ME RIGHT

Channel (Channel Frequency after Pruning)	**NO SAC**	CAR	**GCR**	**SSR**	**GLM ALL**	**GLM BH**
1 (23)	$\beta = 1.62 \pm 0.62$<br>$\mathbf{BF_{10} = 3.32}$	$\beta = -1.61 \pm 0.49$<br>$\mathbf{BF_{10} = 12.46}$	$\beta = -1.05 \pm 0.31$<br>$\mathbf{BF_{10} = 14.42}$	$\beta = 0.38 \pm 0.28$<br>$BF_{10} = 0.50$	$\beta = -0.22 \pm 0.17$<br>$BF_{10} = 0.47$	$\beta = -0.18 \pm 0.30$<br>$BF_{10} = 0.26$
2 (16)	$\beta = 2.86 \pm 0.95$<br>$\mathbf{BF_{10} = 6.15}$	$\beta = -0.13 \pm 0.60$<br>$BF_{10} = 0.26$	$\beta = -0.29 \pm 0.48$<br>$BF_{10} = 0.30$	$\beta = 1.01 \pm 0.51$<br>$BF_{10} = 1.20$	$\beta = 0.42 \pm 0.58$<br>$BF_{10} = 0.32$	$\beta = 0.79 \pm 0.62$<br>$BF_{10} = 0.51$
4 (17)	$\beta = 2.76 \pm 0.61$<br>$\mathbf{BF_{10} = 100.62}$	$\beta = -0.55 \pm 0.21$<br>$\mathbf{BF_{10} = 3.55}$	$\beta = -0.31 \pm 0.19$<br>$BF_{10} = 0.80$	$\beta = 0.71 \pm 0.22$<br>$\mathbf{BF_{10} = 8.00}$	$\beta = 0.10 \pm 0.15$<br>$BF_{10} = 0.30$	$\beta = 0.41 \pm 0.18$<br>$BF_{10} = 1.82$
6 (24)	$\beta = 3.04 \pm 0.61$<br>$\mathbf{BF_{10} = 488.28}$	$\beta = 0.04 \pm 0.36$<br>$BF_{10} = 0.22$	$\beta = -0.65 \pm 0.32$<br>$BF_{10} = 1.29$	$\beta = 1.78 \pm 0.45$<br>$\mathbf{BF_{10} = 53.34}$	$\beta = 1.17 \pm 0.38$<br>$\mathbf{BF_{10} = 8.03}$	$\beta = 1.43 \pm 0.46$<br>$BF_{10} = 8.84$
7 (19)	$\beta = 4.54 \pm 0.65$<br>$\mathbf{BF_{10} = 11.68 \cdot 10^3}$	$\beta = 1.23 \pm 0.31$<br>$\mathbf{BF_{10} = 38.87}$	$\beta = 0.58 \pm 0.31$<br>$BF_{10} = 1.01$	$\beta = 2.58 \pm 0.32$<br>$\mathbf{BF_{10} = 77.57 \cdot 10^3}$	$\beta = 2.09 \pm 0.30$<br>$\mathbf{BF_{10} = 11.47 \cdot 10^3}$	$\beta = 2.31 \pm 0.40$<br>$\mathbf{BF_{10} = 1.35 \cdot 10^3}$
8 (23)	$\beta = 4.59 \pm 0.64$<br>$\mathbf{BF_{10} = 43.09 \cdot 10^3}$	$\beta = 1.39 \pm 0.41$<br>$\mathbf{BF_{10} = 16.25}$	$\beta = 0.75 \pm 0.34$<br>$BF_{10} = 1.69$	$\beta = 2.62 \pm 0.43$<br>$\mathbf{BF_{10} = 4.49 \cdot 10^3}$	$\beta = 2.07 \pm 0.48$<br>$\mathbf{BF_{10} = 105.78}$	$\beta = 2.47 \pm 0.52$<br>$\mathbf{BF_{10} = 299.58}$
10 (17)	$\beta = 2.85 \pm 0.46$<br>$\mathbf{BF_{10} = 1.84 \cdot 10^3}$	$\beta = -0.11 \pm 0.37$<br>$BF_{10} = 0.26$	$\beta = 0.23 \pm 0.24$<br>$BF_{10} = 0.37$	$\beta = 1.29 \pm 0.39$<br>$\mathbf{BF_{10} = 10.15}$	$\beta = 0.22 \pm 0.21$<br>$BF_{10} = 0.40$	$\beta = 0.76 \pm 0.30$<br>$BF_{10} = 2.92$
11 (20)	$\beta = 3.30 \pm 0.54$<br>$\mathbf{BF_{10} = 3.19 \cdot 10^3}$	$\beta = 0.36 \pm 0.16$<br>$BF_{10} = 1.85$	$\beta = 0.59 \pm 0.19$<br>$\mathbf{BF_{10} = 7.11}$	$\beta = 1.62 \pm 0.33$<br>$\mathbf{BF_{10} = 279.53}$	$\beta = 0.79 \pm 0.20$<br>$\mathbf{BF_{10} = 40.48}$	$\beta = 1.29 \pm 0.26$<br>$\mathbf{BF_{10} = 323.23}$
12 (19)	$\beta = 2.92 \pm 0.62$<br>$\mathbf{BF_{10} = 171.55}$	$\beta = 0.09 \pm 0.31$<br>$BF_{10} = 0.25$	$\beta = 0.57 \pm 0.25$<br>$BF_{10} = 1.83$	$\beta = 1.17 \pm 0.37$<br>$\mathbf{BF_{10} = 9.55}$	$\beta = 0.69 \pm 0.25$<br>$\mathbf{BF_{10} = 4.02}$	$\beta = 0.78 \pm 0.33$<br>$BF_{10} = 2.00$
13 (17)	$\beta = 2.56 \pm 0.66$<br>$\mathbf{BF_{10} = 29.33}$	$\beta = -0.29 \pm 0.33$<br>$BF_{10} = 0.35$	$\beta = 0.19 \pm 0.29$<br>$BF_{10} = 0.30$	$\beta = 0.66 \pm 0.25$<br>$\mathbf{BF_{10} = 3.14}$	$\beta = 0.10 \pm 0.23$<br>$BF_{10} = 0.27$	$\beta = 0.53 \pm 0.17$<br>$\mathbf{BF_{10} = 7.96}$
15 (23)	$\beta = 2.42 \pm 0.65$<br>$\mathbf{BF_{10} = 31.62}$	$\beta = -0.67 \pm 0.33$<br>$BF_{10} = 1.26$	$\beta = -0.10 \pm 0.28$<br>$BF_{10} = 0.23$	$\beta = 1.82 \pm 0.52$<br>$\mathbf{BF_{10} = 19.24}$	$\beta = 0.84 \pm 0.37$<br>$BF_{10} = 1.96$	$\beta = 0.86 \pm 0.45$<br>$BF_{10} = 1.02$
17 (20)	$\beta = 4.65 \pm 0.76$<br>$\mathbf{BF_{10} = 2.90 \cdot 10^3}$	$\beta = 1.51 \pm 0.81$<br>$BF_{10} = 0.99$	$\beta = 0.66 \pm 0.66$<br>$BF_{10} = 0.36$	$\beta = 2.55 \pm 0.83$<br>$\mathbf{BF_{10} = 7.81}$	$\beta = 2.06 \pm 0.61$<br>$\mathbf{BF_{10} = 14.00}$	$\beta = 2.42 \pm 0.68$<br>$\mathbf{BF_{10} = 19.54}$
18 (21)	$\beta = 3.96 \pm 0.56$<br>$\mathbf{BF_{10} = 25.38 \cdot 10^3}$	$\beta = 0.70 \pm 0.30$<br>$BF_{10} = 2.07$	$\beta = -0.13 \pm 0.37$<br>$BF_{10} = 0.24$	$\beta = 1.94 \pm 0.38$<br>$\mathbf{BF_{10} = 498.90}$	$\beta = 1.39 \pm 0.33$<br>$\mathbf{BF_{10} = 83.07}$	$\beta = 1.76 \pm 0.40$<br>$\mathbf{BF_{10} = 106.95}$
19 (20)	$\beta = 2.30 \pm 0.67$<br>$\mathbf{BF_{10} = 14.96}$	$\beta = -0.52 \pm 0.40$<br>$BF_{10} = 0.48$	$\beta = -0.54 \pm 0.26$<br>$BF_{10} = 1.30$	$\beta = 0.28 \pm 0.22$<br>$BF_{10} = 0.46$	$\beta = 0.03 \pm 0.22$<br>$BF_{10} = 0.23$	$\beta = 0.11 \pm 0.27$<br>$BF_{10} = 0.25$
21 (23)	$\beta = 2.40 \pm 0.63$<br>$\mathbf{BF_{10} = 35.10}$	$\beta = -0.97 \pm 0.46$<br>$BF_{10} = 1.39$	$\beta = -0.24 \pm 0.33$<br>$BF_{10} = 0.28$	$\beta = 1.01 \pm 0.35$<br>$\mathbf{BF_{10} = 5.96}$	$\beta = 0.38 \pm 0.38$<br>$BF_{10} = 0.34$	$\beta = 0.23 \pm 0.54$<br>$BF_{10} = 0.24$
22 (21)	$\beta = 2.35 \pm 0.75$<br>$\mathbf{BF_{10} = 8.61}$	$\beta = -0.94 \pm 0.42$<br>$BF_{10} = 1.73$	$\beta = -0.34 \pm 0.33$<br>$BF_{10} = 0.36$	$\beta = 0.86 \pm 0.43$<br>$BF_{10} = 1.24$	$\beta = -0.05 \pm 0.23$<br>$BF_{10} = 0.23$	$\beta = -0.20 \pm 0.36$<br>$BF_{10} = 0.26$

Table 24 Results of the Bayesian t-tests of the BETA MAPS analysis of the ME LEFT and ME RIGHT $\Delta[HbR]$ data. Mean ± SEM represent mean beta values of the respective channel and its standard error of the mean across participants, BF_{10} represent Bayes factors. Bayesian t-tests were performed with the *BayesFactor* package in R for which the default prior odds is set at $P(M) = \frac{\sqrt{2}}{2}$. Corresponding BETA MAPS are visualized in Figure 8 B of the main document.

Channel (Channel Frequency after Pruning)	NO SAC	CAR	GCR	SSR	GLM ALL	GLM BH
			**ME LEFT**			
1 (23)	$\beta = -0.11 \pm 0.06$<br>$BF_{10} = 0.82$	$\beta = 0.74 \pm 0.15$<br>$\mathbf{BF_{10} = 445.02}$	$\beta = 0.56 \pm 0.11$<br>$\mathbf{BF_{10} = 579.87}$	$\beta = -0.08 \pm 0.06$<br>$BF_{10} = 0.44$	$\beta = -0.11 \pm 0.07$<br>$BF_{10} = 0.71$	$\beta = -0.16 \pm 0.07$<br>$BF_{10} = 2.27$
2 (16)	$\beta = -0.51 \pm 0.17$<br>$\mathbf{BF_{10} = 5.78}$	$\beta = 0.21 \pm 0.14$<br>$BF_{10} = 0.63$	$\beta = 0.09 \pm 0.11$<br>$BF_{10} = 0.33$	$\beta = -0.44 \pm 0.17$<br>$BF_{10} = 3.29$	$\beta = -0.44 \pm 0.17$<br>$BF_{10} = 3.06$	$\beta = -0.49 \pm 0.18$<br>$\mathbf{BF_{10} = 3.70}$
4 (17)	$\beta = -0.16 \pm 0.07$<br>$BF_{10} = 2.08$	$\beta = 0.44 \pm 0.09$<br>$\mathbf{BF_{10} = 117.86}$	$\beta = 0.29 \pm 0.06$<br>$\mathbf{BF_{10} = 144.66}$	$\beta = -0.08 \pm 0.07$<br>$BF_{10} = 0.44$	$\beta = -0.14 \pm 0.06$<br>$BF_{10} = 1.56$	$\beta = -0.20 \pm 0.10$<br>$BF_{10} = 1.47$
6 (24)	$\beta = -0.73 \pm 0.15$<br>$\mathbf{BF_{10} = 279.06}$	$\beta = 0.04 \pm 0.12$<br>$BF_{10} = 0.22$	$\beta = 0.01 \pm 0.11$<br>$BF_{10} = 0.22$	$\beta = -0.69 \pm 0.15$<br>$\mathbf{BF_{10} = 226.55}$	$\beta = -0.46 \pm 0.10$<br>$\mathbf{BF_{10} = 221.94}$	$\beta = -0.58 \pm 0.13$<br>$\mathbf{BF_{10} = 159.51}$
7 (19)	$\beta = -1.08 \pm 0.18$<br>$\mathbf{BF_{10} = 1572.42}$	$\beta = -0.35 \pm 0.14$<br>$BF_{10} = 2.56$	$\beta = -0.39 \pm 0.10$<br>$\mathbf{BF_{10} = 24.85}$	$\beta = -1.09 \pm 0.18$<br>$\mathbf{BF_{10} = 2897.53}$	$\beta = -0.81 \pm 0.15$<br>$\mathbf{BF_{10} = 570.89}$	$\beta = -0.94 \pm 0.16$<br>$\mathbf{BF_{10} = 1709.00}$
8 (23)	$\beta = -0.77 \pm 0.17$<br>$\mathbf{BF_{10} = 183.92}$	$\beta = 0.02 \pm 0.12$<br>$BF_{10} = 0.22$	$\beta = 0.03 \pm 0.12$<br>$BF_{10} = 0.22$	$\beta = -0.69 \pm 0.16$<br>$\mathbf{BF_{10} = 123.15}$	$\beta = -0.58 \pm 0.19$<br>$\mathbf{BF_{10} = 8.54}$	$\beta = -0.67 \pm 0.18$<br>$\mathbf{BF_{10} = 28.84}$
10 (17)	$\beta = -0.90 \pm 0.23$<br>$\mathbf{BF_{10} = 27.97}$	$\beta = -0.02 \pm 0.12$<br>$BF_{10} = 0.25$	$\beta = -0.15 \pm 0.12$<br>$BF_{10} = 0.48$	$\beta = -0.83 \pm 0.24$<br>$\mathbf{BF_{10} = 13.72}$	$\beta = -0.55 \pm 0.18$<br>$\mathbf{BF_{10} = 6.13}$	$\beta = -0.69 \pm 0.24$<br>$\mathbf{BF_{10} = 5.24}$
11 (20)	$\beta = -0.66 \pm 0.12$<br>$\mathbf{BF_{10} = 1358.85}$	$\beta = -0.05 \pm 0.08$<br>$BF_{10} = 0.27$	$\beta = -0.21 \pm 0.08$<br>$\mathbf{BF_{10} = 3.80}$	$\beta = -0.57 \pm 0.13$<br>$\mathbf{BF_{10} = 103.93}$	$\beta = -0.41 \pm 0.11$<br>$\mathbf{BF_{10} = 23.03}$	$\beta = -0.45 \pm 0.12$<br>$\mathbf{BF_{10} = 21.32}$
12 (19)	$\beta = -0.90 \pm 0.18$<br>$\mathbf{BF_{10} = 370.57}$	$\beta = -0.31 \pm 0.11$<br>$\mathbf{BF_{10} = 3.72}$	$\beta = -0.13 \pm 0.09$<br>$BF_{10} = 0.60$	$\beta = -0.83 \pm 0.18$<br>$\mathbf{BF_{10} = 169.47}$	$\beta = -0.61 \pm 0.12$<br>$\mathbf{BF_{10} = 538.96}$	$\beta = -0.77 \pm 0.16$<br>$\mathbf{BF_{10} = 225.63}$
13 (17)	$\beta = -0.63 \pm 0.17$<br>$\mathbf{BF_{10} = 17.50}$	$\beta = 0.08 \pm 0.13$<br>$BF_{10} = 0.30$	$\beta = 0.21 \pm 0.15$<br>$BF_{10} = 0.58$	$\beta = -0.58 \pm 0.17$<br>$\mathbf{BF_{10} = 11.43}$	$\beta = -0.45 \pm 0.18$<br>$BF_{10} = 2.58$	$\beta = -0.52 \pm 0.23$<br>$BF_{10} = 1.91$
15 (23)	$\beta = -0.86 \pm 0.15$<br>$\mathbf{BF_{10} = 2861.99}$	$\beta = -0.17 \pm 0.15$<br>$BF_{10} = 0.39$	$\beta = 0.04 \pm 0.14$<br>$BF_{10} = 0.23$	$\beta = -1.03 \pm 0.18$<br>$\mathbf{BF_{10} = 2030.39}$	$\beta = -0.74 \pm 0.11$<br>$\mathbf{BF_{10} = 39.20 \cdot 103}$	$\beta = -0.88 \pm 0.15$<br>$\mathbf{BF_{10} = 3147.56}$
17 (20)	$\beta = -1.00 \pm 0.19$<br>$\mathbf{BF_{10} = 507.77}$	$\beta = -0.16 \pm 0.13$<br>$BF_{10} = 0.44$	$\beta = -0.17 \pm 0.09$<br>$BF_{10} = 1.09$	$\beta = -0.88 \pm 0.15$<br>$\mathbf{BF_{10} = 1623.04}$	$\beta = -0.52 \pm 0.14$<br>$\mathbf{BF_{10} = 35.22}$	$\beta = -0.67 \pm 0.14$<br>$\mathbf{BF_{10} = 200.45}$
18 (21)	$\beta = -0.64 \pm 0.11$<br>$\mathbf{BF_{10} = 1134.97}$	$\beta = 0.13 \pm 0.12$<br>$BF_{10} = 0.39$	$\beta = 0.10 \pm 0.09$<br>$BF_{10} = 0.37$	$\beta = -0.57 \pm 0.10$<br>$\mathbf{BF_{10} = 1052.78}$	$\beta = -0.29 \pm 0.07$<br>$\mathbf{BF_{10} = 42.75}$	$\beta = -0.47 \pm 0.10$<br>$\mathbf{BF_{10} = 310.54}$
19 (20)	$\beta = -0.34 \pm 0.11$<br>$\mathbf{BF_{10} = 10.68}$	$\beta = 0.35 \pm 0.11$<br>$\mathbf{BF_{10} = 9.42}$	$\beta = 0.31 \pm 0.09$<br>$\mathbf{BF_{10} = 17.93}$	$\beta = -0.27 \pm 0.08$<br>$\mathbf{BF_{10} = 10.77}$	$\beta = -0.13 \pm 0.05$<br>$\mathbf{BF_{10} = 4.05}$	$\beta = -0.23 \pm 0.07$<br>$\mathbf{BF_{10} = 8.36}$
21 (23)	$\beta = -1.46 \pm 0.30$<br>$\mathbf{BF_{10} = 400.93}$	$\beta = -0.57 \pm 0.17$<br>$\mathbf{BF_{10} = 15.29}$	$\beta = -0.43 \pm 0.13$<br>$\mathbf{BF_{10} = 11.27}$	$\beta = -1.32 \pm 0.25$<br>$\mathbf{BF_{10} = 1043.97}$	$\beta = -1.19 \pm 0.23$<br>$\mathbf{BF_{10} = 625.22}$	$\beta = -1.29 \pm 0.27$<br>$\mathbf{BF_{10} = 340.11}$
22 (21)	$\beta = -0.89 \pm 0.22$<br>$\mathbf{BF_{10} = 47.24}$	$\beta = -0.14 \pm 0.16$<br>$BF_{10} = 0.33$	$\beta = 0.00 \pm 0.10$<br>$BF_{10} = 0.23$	$\beta = -0.75 \pm 0.21$<br>$\mathbf{BF_{10} = 18.80}$	$\beta = -0.65 \pm 0.23$<br>$\mathbf{BF_{10} = 5.02}$	$\beta = -0.89 \pm 0.25$<br>$\mathbf{BF_{10} = 18.58}$
			**ME RIGHT**			
Channel (Channel Frequency after Pruning)	**NO SAC**	CAR	**GCR**	**SSR**	**GLM ALL**	**GLM BH**
1 (23)	$\beta = -0.03 \pm 0.08$<br>$BF_{10} = 0.24$	$\beta = 0.82 \pm 0.17$<br>$\mathbf{BF_{10} = 209.78}$	$\beta = 0.57 \pm 0.12$<br>$\mathbf{BF_{10} = 386.50}$	$\beta = 0.02 \pm 0.08$<br>$BF_{10} = 0.23$	$\beta = 0.03 \pm 0.06$<br>$BF_{10} = 0.24$	$\beta = -0.01 \pm 0.07$<br>$BF_{10} = 0.22$
2 (16)	$\beta = -0.46 \pm 0.14$<br>$\mathbf{BF_{10} = 11.51}$	$\beta = 0.22 \pm 0.13$<br>$BF_{10} = 0.81$	$\beta = 0.16 \pm 0.11$<br>$BF_{10} = 0.58$	$\beta = -0.37 \pm 0.13$<br>$\mathbf{BF_{10} = 3.80}$	$\beta = -0.42 \pm 0.17$<br>$BF_{10} = 2.86$	$\beta = -0.46 \pm 0.14$<br>$\mathbf{BF_{10} = 10.08}$
4 (17)	$\beta = -0.13 \pm 0.08$<br>$BF_{10} = 0.77$	$\beta = 0.49 \pm 0.11$<br>$\mathbf{BF_{10} = 66.56}$	$\beta = 0.33 \pm 0.09$<br>$\mathbf{BF_{10} = 19.30}$	$\beta = -0.06 \pm 0.07$<br>$BF_{10} = 0.32$	$\beta = -0.08 \pm 0.06$<br>$BF_{10} = 0.56$	$\beta = -0.15 \pm 0.09$<br>$BF_{10} = 0.77$
6 (24)	$\beta = -0.83 \pm 0.21$<br>$\mathbf{BF_{10} = 48.42}$	$\beta = -0.11 \pm 0.12$<br>$BF_{10} = 0.31$	$\beta = 0.16 \pm 0.12$<br>$BF_{10} = 0.44$	$\beta = -0.83 \pm 0.21$<br>$\mathbf{BF_{10} = 41.68}$	$\beta = -0.62 \pm 0.16$<br>$\mathbf{BF_{10} = 49.24}$	$\beta = -0.70 \pm 0.18$<br>$\mathbf{BF_{10} = 54.63}$
7 (19)	$\beta = -1.15 \pm 0.21$<br>$\mathbf{BF_{10} = 695.14}$	$\beta = -0.40 \pm 0.15$<br>$\mathbf{BF_{10} = 3.93}$	$\beta = -0.14 \pm 0.15$<br>$BF_{10} = 0.35$	$\beta = -1.07 \pm 0.22$<br>$\mathbf{BF_{10} = 286.24}$	$\beta = -0.89 \pm 0.19$<br>$\mathbf{BF_{10} = 196.74}$	$\beta = -0.99 \pm 0.21$<br>$\mathbf{BF_{10} = 157.14}$
8 (23)	$\beta = -1.23 \pm 0.22$<br>$\mathbf{BF_{10} = 1.70 \cdot 10^3}$	$\beta = -0.45 \pm 0.15$<br>$\mathbf{BF_{10} = 8.15}$	$\beta = -0.19 \pm 0.13$<br>$BF_{10} = 0.59$	$\beta = -1.15 \pm 0.20$<br>$\mathbf{BF_{10} = 2.10 \cdot 10^3}$	$\beta = -0.97 \pm 0.22$<br>$\mathbf{BF_{10} = 133.05}$	$\beta = -1.05 \pm 0.21$<br>$\mathbf{BF_{10} = 406.28}$
10 (17)	$\beta = -0.69 \pm 0.16$<br>$\mathbf{BF_{10} = 72.47}$	$\beta = 0.04 \pm 0.10$<br>$BF_{10} = 0.26$	$\beta = -0.13 \pm 0.10$<br>$BF_{10} = 0.51$	$\beta = -0.53 \pm 0.15$<br>$\mathbf{BF_{10} = 18.71}$	$\beta = -0.44 \pm 0.23$<br>$BF_{10} = 1.09$	$\beta = -0.44 \pm 0.14$<br>$\mathbf{BF_{10} = 7.38}$
11 (20)	$\beta = -0.92 \pm 0.21$<br>$\mathbf{BF_{10} = 88.97}$	$\beta = -0.13 \pm 0.11$<br>$BF_{10} = 0.42$	$\beta = -0.33 \pm 0.12$<br>$\mathbf{BF_{10} = 4.35}$	$\beta = -0.76 \pm 0.22$<br>$\mathbf{BF_{10} = 15.01}$	$\beta = -0.56 \pm 0.21$<br>$\mathbf{BF_{10} = 3.89}$	$\beta = -0.64 \pm 0.21$<br>$\mathbf{BF_{10} = 6.50}$
12 (19)	$\beta = -0.88 \pm 0.17$<br>$\mathbf{BF_{10} = 572.69}$	$\beta = -0.23 \pm 0.12$<br>$BF_{10} = 1.07$	$\beta = -0.27 \pm 0.11$<br>$BF_{10} = 2.53$	$\beta = -0.71 \pm 0.18$<br>$\mathbf{BF_{10} = 33.83}$	$\beta = -0.54 \pm 0.14$<br>$\mathbf{BF_{10} = 40.72}$	$\beta = -0.67 \pm 0.18$<br>$\mathbf{BF_{10} = 30.09}$
13 (17)	$\beta = -0.16 \pm 0.11$<br>$BF_{10} = 0.59$	$\beta = 0.40 \pm 0.09$<br>$\mathbf{BF_{10} = 53.05}$	$\beta = 0.25 \pm 0.09$<br>$BF_{10} = 3.64$	$\beta = -0.14 \pm 0.08$<br>$BF_{10} = 0.88$	$\beta = -0.20 \pm 0.21$<br>$BF_{10} = 0.38$	$\beta = -0.11 \pm 0.13$<br>$BF_{10} = 0.35$
15 (23)	$\beta = -0.69 \pm 0.17$<br>$\mathbf{BF_{10} = 71.46}$	$\beta = 0.07 \pm 0.15$<br>$BF_{10} = 0.42$	$\beta = -0.11 \pm 0.11$<br>$BF_{10} = 0.34$	$\beta = -0.71 \pm 0.17$<br>$\mathbf{BF_{10} = 90.63}$	$\beta = -0.51 \pm 0.13$<br>$\mathbf{BF_{10} = 52.21}$	$\beta = -0.64 \pm 0.16$<br>$\mathbf{BF_{10} = 44.04}$
17 (20)	$\beta = -1.90 \pm 0.67$<br>$\mathbf{BF_{10} = 4.85}$	$\beta = -0.95 \pm 0.50$<br>$BF_{10} = 1.05$	$\beta = -0.54 \pm 0.37$<br>$BF_{10} = 0.58$	$\beta = -1.73 \pm 0.69$<br>$BF_{10} = 2.72$	$\beta = -1.22 \pm 0.51$<br>$\mathbf{BF_{10} = 2.27}$	$\beta = -1.49 \pm 0.66$<br>$BF_{10} = 1.74$
18 (21)	$\beta = -1.19 \pm 0.20$<br>$\mathbf{BF_{10} = 30.03 \cdot 10^3}$	$\beta = -0.29 \pm 0.12$<br>$BF_{10} = 2.46$	$\beta = 0.08 \pm 0.19$<br>$BF_{10} = 0.25$	$\beta = -1.03 \pm 0.20$<br>$\mathbf{BF_{10} = 602.32}$	$\beta = -0.71 \pm 0.16$<br>$\mathbf{BF_{10} = 101.70}$	$\beta = -0.95 \pm 0.20$<br>$\mathbf{BF_{10} = 264.48}$
19 (20)	$\beta = -0.24 \pm 0.15$<br>$BF_{10} = 0.67$	$\beta = 0.39 \pm 0.13$<br>$\mathbf{BF_{10} = 7.54}$	$\beta = 0.35 \pm 0.10$<br>$\mathbf{BF_{10} = 18.18}$	$\beta = -0.15 \pm 0.08$<br>$BF_{10} = 0.84$	$\beta = -0.10 \pm 0.05$<br>$BF_{10} = 1.10$	$\beta = -0.13 \pm 0.10$<br>$BF_{10} = 0.53$
21 (23)	$\beta = -0.75 \pm 0.16$<br>$\mathbf{BF_{10} = 194.00}$	$\beta = 0.04 \pm 0.12$<br>$BF_{10} = 0.23$	$\beta = -0.11 \pm 0.10$<br>$BF_{10} = 0.39$	$\beta = -0.59 \pm 0.15$<br>$\mathbf{BF_{10} = 53.12}$	$\beta = -0.46 \pm 0.12$<br>$\mathbf{BF_{10} = 47.02}$	$\beta = -0.57 \pm 0.16$<br>$\mathbf{BF_{10} = 20.48}$
22 (21)	$\beta = -0.41 \pm 0.12$<br>$\mathbf{BF_{10} = 16.41}$	$\beta = 0.27 \pm 0.08$<br>$\mathbf{BF_{10} = 11.84}$	$\beta = 0.14 \pm 0.07$<br>$BF_{10} = 1.20$	$\beta = -0.21 \pm 0.12$<br>$BF_{10} = 0.87$	$\beta = -0.18 \pm 0.08$<br>$BF_{10} = 1.71$	$\beta = -0.33 \pm 0.11$<br>$\mathbf{BF_{10} = 6.66}$

Table 25 Results of the Bayesian t-tests of the BETA MAPS analysis of the MI $\Delta[HbO]$ data. Mean $\pm$ SEM represent mean beta values of the respective channel and its standard error of the mean across participants, BF_{10} represent Bayes factors. Bayesian t-tests were performed with the *BayesFactor* package in R for which the default prior odds is set at $P(M) = \frac{\sqrt{2}}{2}$. Corresponding BETA MAPS are visualized in Figure 10 B of the main document.

	MI					
Channel (Channel Frequency after Pruning)	**NO SAC**	**CAR**	**GCR**	**SSR**	**GLM ALL**	**GLM BH**
1 (23)	$\beta = 1.33 \pm 0.39$ $\mathbf{BF_{10} = 15.67}$	$\beta = -0.72 \pm 0.23$ $\mathbf{BF_{10} = 8.88}$	$\beta = -0.55 \pm 0.19$ $\mathbf{BF_{10} = 5.73}$	$\beta = 0.39 \pm 0.14$ $\mathbf{BF_{10} = 4.42}$	$\beta = 0.06 \pm 0.08$ $BF_{10} = 0.27$	$\beta = 0.12 \pm 0.16$ $BF_{10} = 0.28$
2 (16)	$\beta = 1.69 \pm 0.47$ $\mathbf{BF_{10} = 16.02}$	$\beta = 0.06 \pm 0.27$ $BF_{10} = 0.26$	$\beta = 0.00 \pm 0.21$ $BF_{10} = 0.25$	$\beta = 0.82 \pm 0.33$ $BF_{10} = 2.48$	$\beta = 0.55 \pm 0.30$ $BF_{10} = 1.01$	$\beta = 0.61 \pm 0.29$ $BF_{10} = 1.45$
4 (17)	$\beta = 1.87 \pm 0.42$ $\mathbf{BF_{10} = 79.26}$	$\beta = -0.37 \pm 0.18$ $BF_{10} = 1.37$	$\beta = -0.25 \pm 0.13$ $BF_{10} = 1.01$	$\beta = 0.44 \pm 0.15$ $\mathbf{BF_{10} = 4.62}$	$\beta = 0.27 \pm 0.12$ $BF_{10} = 1.81$	$\beta = 0.35 \pm 0.11$ $BF_{10} = 10.06$
6 (24)	$\beta = 2.24 \pm 0.38$ $\mathbf{BF_{10} = 4086.44}$	$\beta = 0.11 \pm 0.15$ $BF_{10} = 0.28$	$\beta = -0.08 \pm 0.12$ $BF_{10} = 0.27$	$\beta = 1.19 \pm 0.23$ $\mathbf{BF_{10} = 644.22}$	$\beta = 0.77 \pm 0.20$ $\mathbf{BF_{10} = 43.83}$	$\beta = 0.99 \pm 0.24$ $\mathbf{BF_{10} = 88.39}$
7 (19)	$\beta = 2.80 \pm 0.50$ $\mathbf{BF_{10} = 998.17}$	$\beta = 0.68 \pm 0.18$ $\mathbf{BF_{10} = 29.27}$	$\beta = 0.45 \pm 0.16$ $\mathbf{BF_{10} = 5.01}$	$\beta = 1.56 \pm 0.27$ $\mathbf{BF_{10} = 1443.28}$	$\beta = 1.18 \pm 0.27$ $\mathbf{BF_{10} = 86.42}$	$\beta = 1.32 \pm 0.31$ $\mathbf{BF_{10} = 68.68}$
8 (23)	$\beta = 2.35 \pm 0.44$ $\mathbf{BF_{10} = 1084.66}$	$\beta = 0.09 \pm 0.19$ $BF_{10} = 0.24$	$\beta = -0.09 \pm 0.16$ $BF_{10} = 0.25$	$\beta = 0.94 \pm 0.23$ $\mathbf{BF_{10} = 70.38}$	$\beta = 0.58 \pm 0.24$ $BF_{10} = 2.53$	$\beta = 0.72 \pm 0.22$ $\mathbf{BF_{10} = 11.79}$
10 (17)	$\beta = 2.26 \pm 0.46$ $\mathbf{BF_{10} = 189.59}$	$\beta = 0.33 \pm 0.30$ $BF_{10} = 0.41$	$\beta = 0.35 \pm 0.26$ $BF_{10} = 0.54$	$\beta = 0.92 \pm 0.25$ $\mathbf{BF_{10} = 21.59}$	$\beta = 0.66 \pm 0.21$ $\mathbf{BF_{10} = 7.21}$	$\beta = 0.82 \pm 0.28$ $\mathbf{BF_{10} = 5.47}$
11 (20)	$\beta = 2.00 \pm 0.43$ $\mathbf{BF_{10} = 153.19}$	$\beta = 0.00 \pm 0.11$ $BF_{10} = 0.22$	$\beta = 0.08 \pm 0.15$ $BF_{10} = 0.27$	$\beta = 0.60 \pm 0.21$ $\mathbf{BF_{10} = 4.59}$	$\beta = 0.43 \pm 0.15$ $\mathbf{BF_{10} = 4.46}$	$\beta = 0.45 \pm 0.17$ $\mathbf{BF_{10} = 3.56}$
12 (19)	$\beta = 2.17 \pm 0.43$ $\mathbf{BF_{10} = 301.58}$	$\beta = 0.35 \pm 0.17$ $BF_{10} = 1.31$	$\beta = 0.33 \pm 0.14$ $BF_{10} = 2.38$	$\beta = 0.88 \pm 0.26$ $\mathbf{BF_{10} = 12.73}$	$\beta = 0.55 \pm 0.15$ $\mathbf{BF_{10} = 21.22}$	$\beta = 0.73 \pm 0.20$ $\mathbf{BF_{10} = 24.02}$
13 (17)	$\beta = 2.03 \pm 0.42$ $\mathbf{BF_{10} = 156.26}$	$\beta = -0.13 \pm 0.18$ $BF_{10} = 0.32$	$\beta = -0.01 \pm 0.20$ $BF_{10} = 0.25$	$\beta = 0.59 \pm 0.23$ $BF_{10} = 2.78$	$\beta = 0.38 \pm 0.14$ $\mathbf{BF_{10} = 4.04}$	$\beta = 0.52 \pm 0.15$ $\mathbf{BF_{10} = 12.75}$
15 (23)	$\beta = 2.22 \pm 0.41$ $\mathbf{BF_{10} = 1201.92}$	$\beta = 0.07 \pm 0.21$ $BF_{10} = 0.23$	$\beta = 0.19 \pm 0.17$ $BF_{10} = 0.37$	$\beta = 1.34 \pm 0.29$ $\mathbf{BF_{10} = 238.83}$	$\beta = 0.71 \pm 0.23$ $\mathbf{BF_{10} = 8.55}$	$\beta = 1.16 \pm 0.31$ $\mathbf{BF_{10} = 28.62}$
17 (20)	$\beta = 2.87 \pm 0.57$ $\mathbf{BF_{10} = 387.49}$	$\beta = 0.64 \pm 0.41$ $BF_{10} = 0.67$	$\beta = 0.48 \pm 0.33$ $BF_{10} = 0.58$	$\beta = 1.41 \pm 0.48$ $\mathbf{BF_{10} = 5.60}$	$\beta = 1.10 \pm 0.41$ $\mathbf{BF_{10} = 3.69}$	$\beta = 1.14 \pm 0.45$ $BF_{10} = 2.84$
18 (21)	$\beta = 1.81 \pm 0.34$ $\mathbf{BF_{10} = 838.01}$	$\beta = -0.25 \pm 0.18$ $BF_{10} = 0.53$	$\beta = -043 \pm 0.20$ $BF_{10} = 1.45$	$\beta = 0.45 \pm 0.14$ $\mathbf{BF_{10} = 12.37}$	$\beta = 0.16 \pm 0.11$ $BF_{10} = 0.58$	$\beta = 0.30 \pm 0.13$ $BF_{10} = 1.93$
19 (20)	$\beta = 1.35 \pm 0.43$ $\mathbf{BF_{10} = 8.75}$	$\beta = -0.48 \pm 0.21$ $BF_{10} = 1.89$	$\beta = -0.26 \pm 0.11$ $BF_{10} = 1.85$	$\beta = 0.12 \pm 0.17$ $BF_{10} = 0.29$	$\beta = -0.09 \pm 0.11$ $BF_{10} = 0.32$	$\beta = -0.05 \pm 0.18$ $BF_{10} = 0.24$
21 (23)	$\beta = 1.77 \pm 0.44$ $\mathbf{BF_{10} = 58.50}$	$\beta = -0.24 \pm 0.23$ $BF_{10} = 0.35$	$\beta = -0.06 \pm 0.16$ $BF_{10} = 0.24$	$\beta = 0.72 \pm 0.18$ $\mathbf{BF_{10} = 45.69}$	$\beta = 0.41 \pm 0.17$ $BF_{10} = 2.11$	$\beta = 0.49 \pm 0.24$ $BF_{10} = 1.25$
22 (21)	$\beta = 1.19 \pm 0.57$ $BF_{10} = 1.40$	$\beta = -0.70 \pm 0.33$ $BF_{10} = 1.39$	$\beta = -0.46 \pm 0.25$ $BF_{10} = 0.94$	$\beta = 0.19 \pm 0.30$ $BF_{10} = 0.27$	$\beta = -0.20 \pm 0.17$ $BF_{10} = 0.44$	$\beta = -0.23 \pm 0.27$ $BF_{10} = 0.32$

Table 26 Results of the Bayesian t-tests of the BETA MAPS analysis of the $\Delta[HbR]$ of MI data. Mean ± SEM represent mean beta values of the respective channel and its standard error of the mean across participants, BF_{10} represent Bayes factors. Bayesian t-tests were performed with the *BayesFactor* package in R for which the default prior odds is set at $P(M) = \frac{\sqrt{2}}{2}$. Corresponding BETA MAPS are visualized in Figure 10 B of the main document.

	MI					
Channel (Channel Frequency after Pruning)	**NO SAC**	**CAR**	**GCR**	**SSR**	**GLM ALL**	**GLM BH**
1 (23)	$\beta = -0.03 \pm 0.04$<br>$BF_{10} = 0.26$	$\beta = 0.34 \pm 0.06$<br>$\mathbf{BF_{10} = 983.89}$	$\beta = 0.30 \pm 0.06$<br>$\mathbf{BF_{10} = 989.14}$	$\beta = 0.01 \pm 0.04$<br>$BF_{10} = 0.22$	$\beta = 0.00 \pm 0.03$<br>$BF_{10} = 0.22$	$\beta = -0.03 \pm 0.04$<br>$BF_{10} = 0.27$
2 (16)	$\beta = -0.19 \pm 0.08$<br>$BF_{10} = 1.86$	$\beta = 0.11 \pm 0.08$<br>$BF_{10} = 0.62$	$\beta = 0.10 \pm 0.06$<br>$BF_{10} = 0.75$	$\beta = -0.13 \pm 0.09$<br>$BF_{10} = 0.64$	$\beta = -0.16 \pm 0.09$<br>$BF_{10} = 0.86$	$\beta = -0.17 \pm 0.08$<br>$BF_{10} = 1.39$
4 (17)	$\beta = -0.14 \pm 0.05$<br>$\mathbf{BF_{10} = 3.85}$	$\beta = 0.15 \pm 0.05$<br>$\mathbf{BF_{10} = 12.71}$	$\beta = 0.11 \pm 0.04$<br>$\mathbf{BF_{10} = 10.30}$	$\beta = -0.07 \pm 0.05$<br>$BF_{10} = 0.64$	$\beta = -0.10 \pm 0.05$<br>$BF_{10} = 1.35$	$\beta = -0.13 \pm 0.05$<br>$BF_{10} = 2.74$
6 (24)	$\beta = -0.67 \pm 0.10$<br>$\mathbf{BF_{10} = 13.94 \cdot 10^3}$	$\beta = -0.31 \pm 0.07$<br>$\mathbf{BF_{10} = 88.67}$	$\beta = -0.21 \pm 0.08$<br>$\mathbf{BF_{10} = 3.91}$	$\beta = -0.64 \pm 0.10$<br>$\mathbf{BF_{10} = 14.03 \cdot 10^3}$	$\beta = -0.49 \pm 0.06$<br>$\mathbf{BF_{10} = 134 \cdot 10^3}$	$\beta = -0.56 \pm 0.09$<br>$\mathbf{BF_{10} = 17.26 \cdot 10^3}$
7 (19)	$\beta = -0.63 \pm 0.09$<br>$\mathbf{BF_{10} = 12.46 \cdot 10^3}$	$\beta = -0.36 \pm 0.07$<br>$\mathbf{BF_{10} = 206.83}$	$\beta = -0.26 \pm 0.05$<br>$\mathbf{BF_{10} = 379.26}$	$\beta = -0.63 \pm 0.10$<br>$\mathbf{BF_{10} = 1.98 \cdot 10^3}$	$\beta = -0.47 \pm 0.09$<br>$\mathbf{BF_{10} = 404.74}$	$\beta = -0.51 \pm 0.08$<br>$\mathbf{BF_{10} = 2.35 \cdot 10^3}$
8 (23)	$\beta = -0.41 \pm 0.10$<br>$\mathbf{BF_{10} = 78.33}$	$\beta = -0.03 \pm 0.07$<br>$BF_{10} = 0.23$	$\beta = 0.06 \pm 0.06$<br>$BF_{10} = 0.32$	$\beta = -0.36 \pm 0.11$<br>$\mathbf{BF_{10} = 10.39}$	$\beta = -0.29 \pm 0.11$<br>$\mathbf{BF_{10} = 3.41}$	$\beta = -0.33 \pm 0.09$<br>$\mathbf{BF_{10} = 36.12}$
10 (17)	$\beta = -0.55 \pm 0.16$<br>$\mathbf{BF_{10} = 15.51}$	$\beta = -0.18 \pm 0.11$<br>$BF_{10} = 0.74$	$\beta = -0.20 \pm 0.11$<br>$BF_{10} = 1.08$	$\beta = -0.46 \pm 0.15$<br>$\mathbf{BF_{10} = 7.41}$	$\beta = -0.37 \pm 0.15$<br>$\mathbf{BF_{10} = 2.58}$	$\beta = -0.40 \pm 0.13$<br>$\mathbf{BF_{10} = 7.36}$
11 (20)	$\beta = -0.35 \pm 0.05$<br>$\mathbf{BF_{10} = 61.24 \cdot 10^3}$	$\beta = -0.08 \pm 0.03$<br>$\mathbf{BF_{10} = 4.26}$	$\beta = -0.10 \pm 0.03$<br>$\mathbf{BF_{10} = 15.84}$	$\beta = -0.28 \pm 0.06$<br>$\mathbf{BF_{10} = 220.29}$	$\beta = -0.16 \pm 0.04$<br>$\mathbf{BF_{10} = 62.25}$	$\beta = -0.22 \pm 0.03$<br>$\mathbf{BF_{10} = 8.73 \cdot 0^3}$
12 (19)	$\beta = -0.43 \pm 0.08$<br>$\mathbf{BF_{10} = 445.39}$	$\beta = -0.14 \pm 0.06$<br>$BF_{10} = 2.19$	$\beta = -0.13 \pm 0.04$<br>$\mathbf{BF_{10} = 4.93}$	$\beta = -0.36 \pm 0.08$<br>$\mathbf{BF_{10} = 86.82}$	$\beta = -0.24 \pm 0.05$<br>$\mathbf{BF_{10} = 167.63}$	$\beta = -0.31 \pm 0.07$<br>$\mathbf{BF_{10} = 129.49}$
13 (17)	$\beta = -0.15 \pm 0.07$<br>$BF_{10} = 1.88$	$\beta = 0.18 \pm 0.05$<br>$\mathbf{BF_{10} = 28.98}$	$\beta = 0.11 \pm 0.04$<br>$\mathbf{BF_{10} = 5.99}$	$\beta = -0.15 \pm 0.06$<br>$BF_{10} = 2.91$	$\beta = -0.07 \pm 0.07$<br>$BF_{10} = 0.36$	$\beta = -0.09 \pm 0.05$<br>$\mathbf{BF_{10} = 70.98}$
15 (23)	$\beta = -0.48 \pm 0.10$<br>$\mathbf{BF_{10} = 532.27}$	$\beta = -0.15 \pm 0.09$<br>$BF_{10} = 0.78$	$\beta = -0.16 \pm 0.06$<br>$BF_{10} = 2.38$	$\beta = -0.49 \pm 0.09$<br>$\mathbf{BF_{10} = 865.75}$	$\beta = -0.37 \pm 0.08$<br>$\mathbf{BF_{10} = 185.36}$	$\beta = -0.44 \pm 0.09$<br>$\mathbf{BF_{10} = 357.51}$
17 (20)	$\beta = -0.47 \pm 0.12$<br>$\mathbf{BF_{10} = 31.40}$	$\beta = -0.12 \pm 0.10$<br>$BF_{10} = 0.41$	$\beta = -0.04 \pm 0.08$<br>$BF_{10} = 0.26$	$\beta = -0.40 \pm 0.12$<br>$\mathbf{BF_{10} = 12.86}$	$\beta = -0.20 \pm 0.14$<br>$BF_{10} = 0.60$	$\beta = -0.27 \pm 0.13$<br>$BF_{10} = 1.22$
18 (21)	$\beta = -0.22 \pm 0.05$<br>$\mathbf{BF_{10} = 96.37}$	$\beta = 0.11 \pm 0.06$<br>$BF_{10} = 1.00$	$\beta = 0.15 \pm 0.04$<br>$\mathbf{BF_{10} = 18.69}$	$\beta = -0.15 \pm 0.04$<br>$\mathbf{BF_{10} = 36.97}$	$\beta = -0.02 \pm 0.04$<br>$BF_{10} = 0.25$	$\beta = -0.09 \pm 0.04$<br>$BF_{10} = 1.38$
19 (20)	$\beta = -0.07 \pm 0.09$<br>$BF_{10} = 0.30$	$\beta = 0.25 \pm 0.09$<br>$\mathbf{BF_{10} = 4.91}$	$\beta = 0.16 \pm 0.06$<br>$\mathbf{BF_{10} = 4.16}$	$\beta = 0.00 \pm 0.07$<br>$BF_{10} = 0.23$	$\beta = 0.02 \pm 0.05$<br>$BF_{10} = 0.25$	$\beta = -0.01 \pm 0.06$<br>$BF_{10} = 0.24$
21 (23)	$\beta = -0.33 \pm 0.08$<br>$\mathbf{BF_{10} = 116.08}$	$\beta = 0.03 \pm 0.04$<br>$BF_{10} = 0.25$	$\beta = 0.00 \pm 0.04$<br>$BF_{10} = 0.22$	$\beta = -0.28 \pm 0.08$<br>$\mathbf{BF_{10} = 28.90}$	$\beta = -0.18 \pm 0.06$<br>$\mathbf{BF_{10} = 5.67}$	$\beta = -0.21 \pm 0.06$<br>$\mathbf{BF_{10} = 15.06}$
22 (21)	$\beta = -0.12 \pm 0.09$<br>$BF_{10} = 0.47$	$\beta = 0.20 \pm 0.08$<br>$\mathbf{BF_{10} = 3.19}$	$\beta = 0.13 \pm 0.06$<br>$BF_{10} = 1.40$	$\beta = -0.03 \pm 0.08$<br>$BF_{10} = 0.24$	$\beta = 0.01 \pm 0.06$<br>$BF_{10} = 0.23$	$\beta = -0.04 \pm 0.08$<br>$BF_{10} = 0.25$

Single Trial Time Series

SIM data

The following figures visualize the normalized single-subject single-trial semi-simulated $\Delta[HbO]$ and $\Delta[HbR]$ data of each individually selected channel (based on beta values of GLM ALL corrected data) of all correction methods and all SDCs.

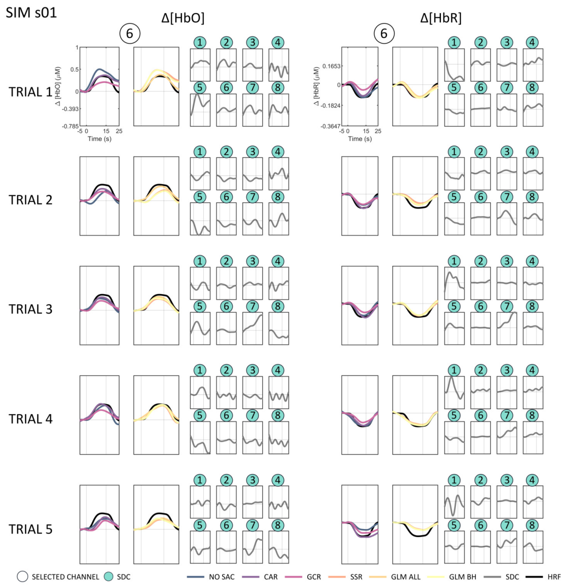

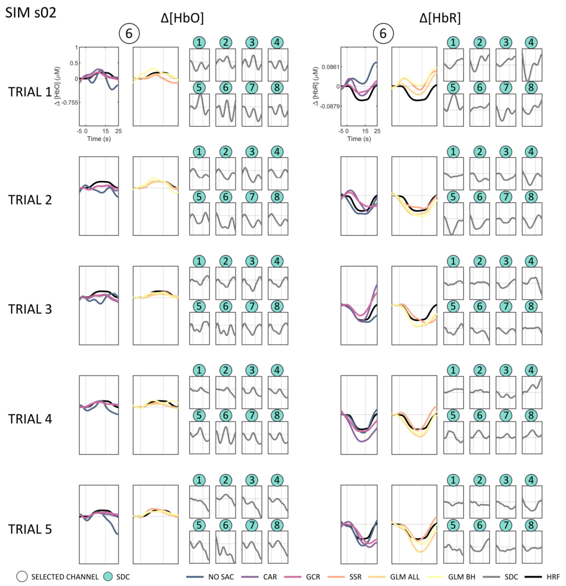

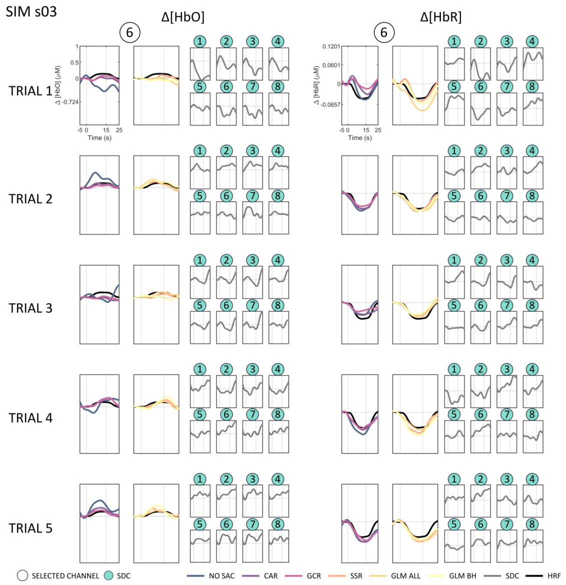

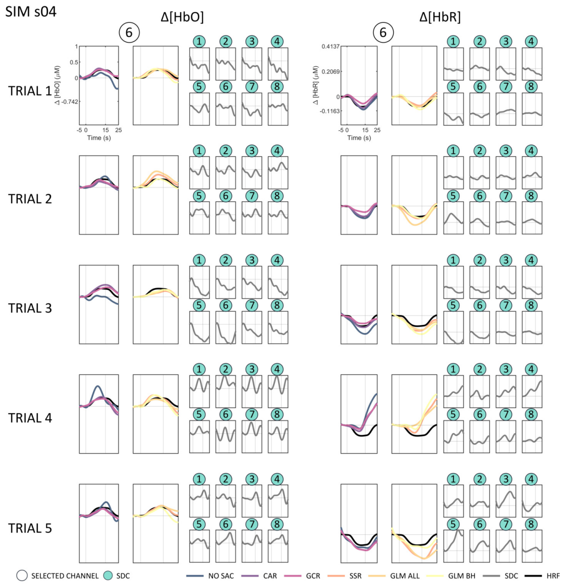

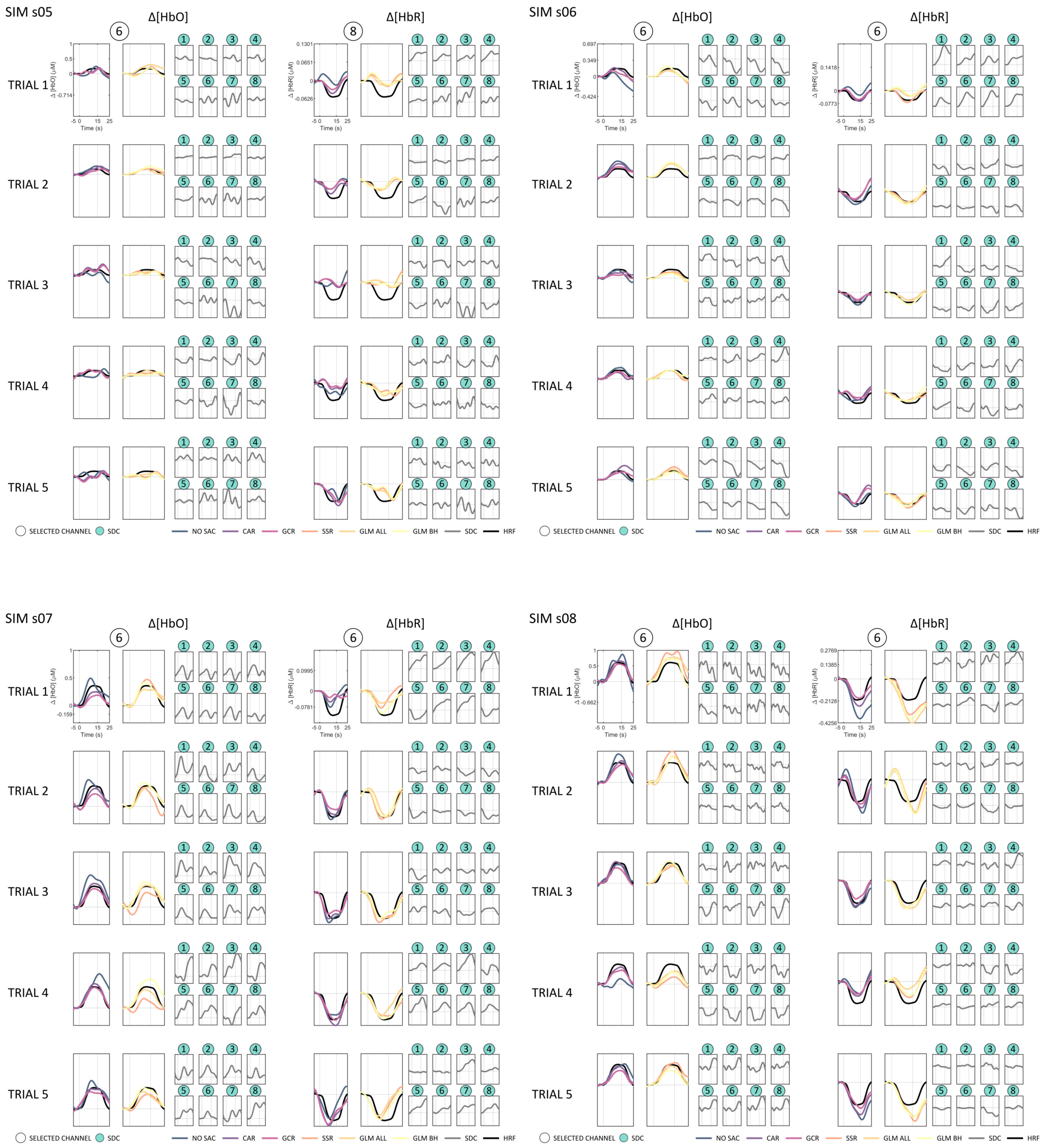
SIM s05
SIM s06
SIM s07
SIM s08
Δ[HbO]
Δ[HbR]
TRIAL 1
TRIAL 2
TRIAL 3
TRIAL 4
TRIAL 5
Time (s)
SELECTED CHANNEL
SDC
NO SAC
CAR
GCR
SSR
GLM ALL
GLM BH
SDC
HRF

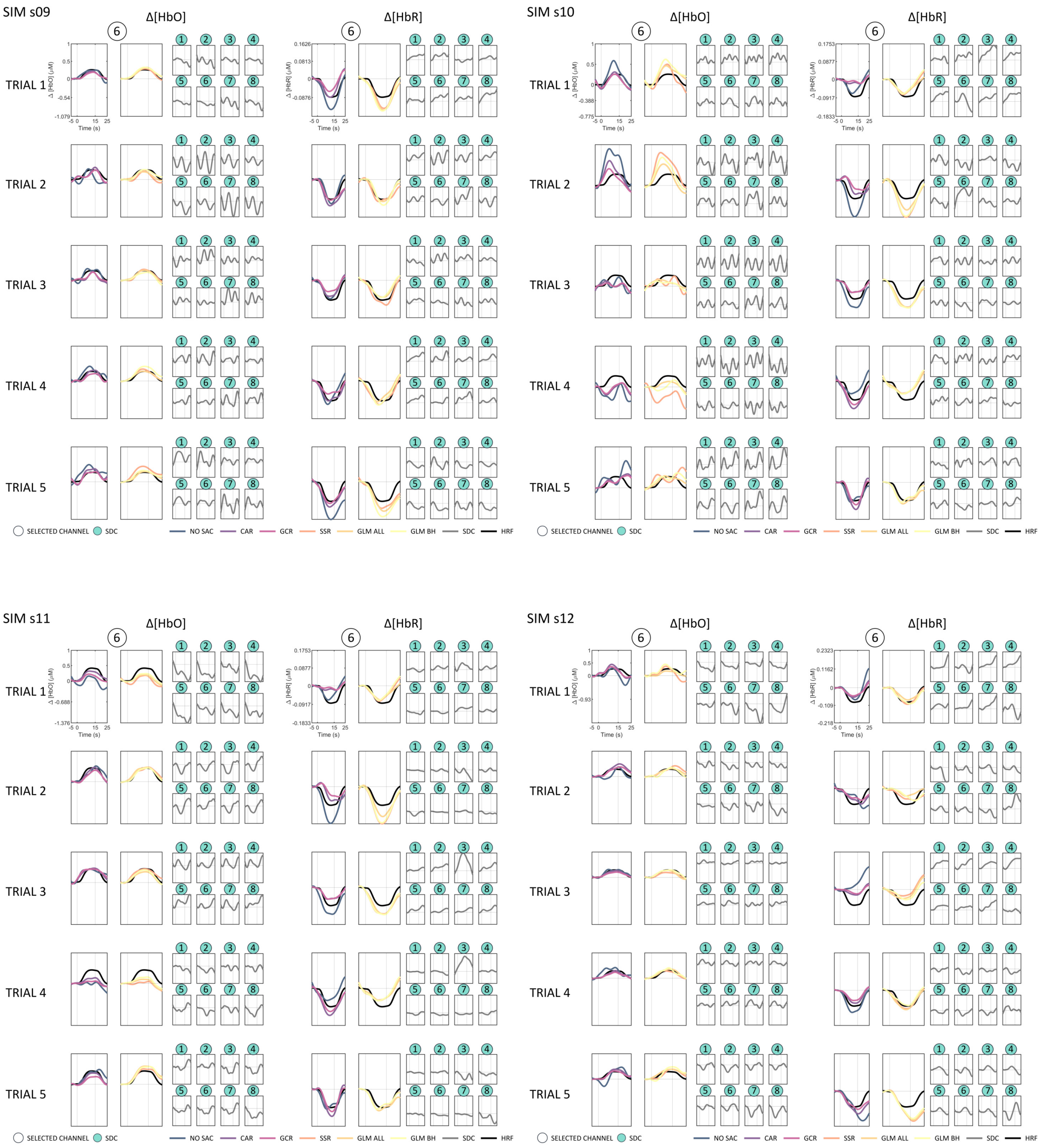
SIM s09
Δ[HbO]
Δ[HbR]
TRIAL 1
TRIAL 2
TRIAL 3
TRIAL 4
TRIAL 5
SELECTED CHANNEL
SDC
NO SAC
CAR
GCR
SSR
GLM ALL
GLM BH
SDC
HRF
SIM s10
SIM s11
SIM s12
Time (s)
Δ [HbO] (μM)
Δ [HbR] (μM)

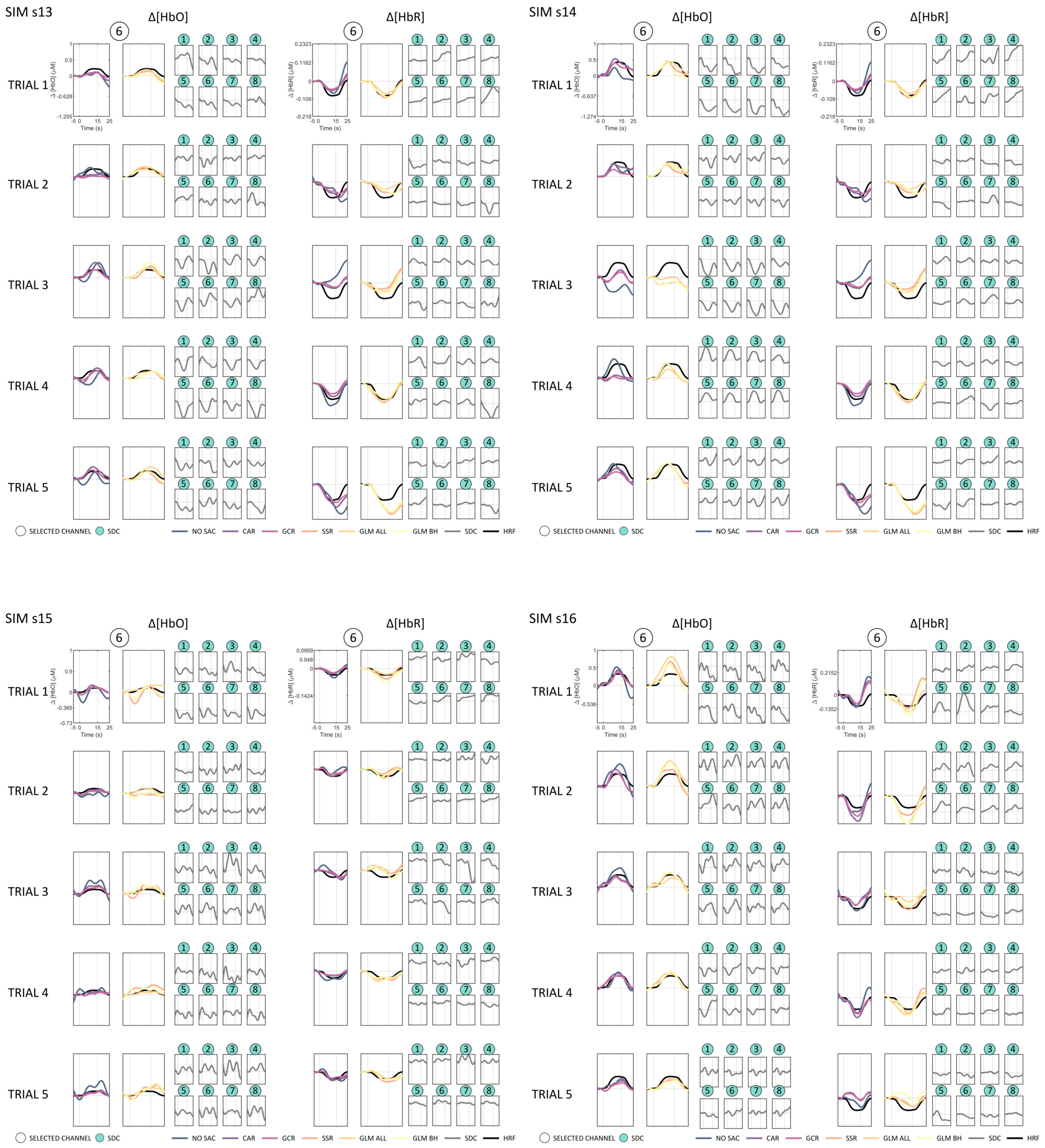
SIM s13
Δ[HbO]
Δ[HbR]
SIM s14
SIM s15
SIM s16
TRIAL 1
TRIAL 2
TRIAL 3
TRIAL 4
TRIAL 5
Time (s)
SELECTED CHANNEL
SDC
NO SAC
CAR
GCR
SSR
GLM ALL
GLM BH
SDC
HRF

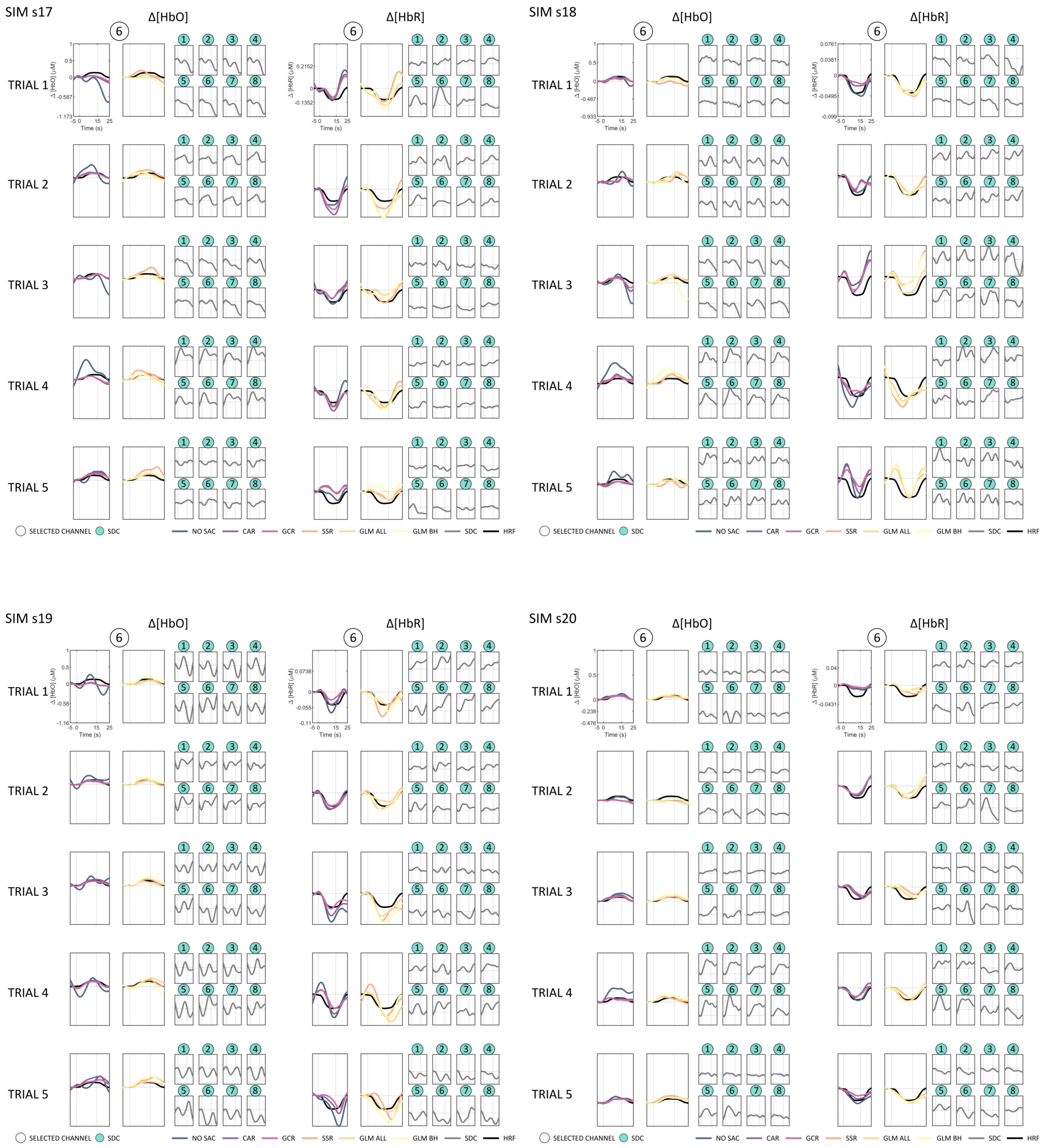
SIM s17
Δ[HbO]
Δ[HbR]
TRIAL 1
TRIAL 2
TRIAL 3
TRIAL 4
TRIAL 5
SELECTED CHANNEL
SDC
NO SAC
CAR
GCR
SSR
GLM ALL
GLM BH
SDC
HRF
SIM s18
SIM s19
SIM s20
Time (s)

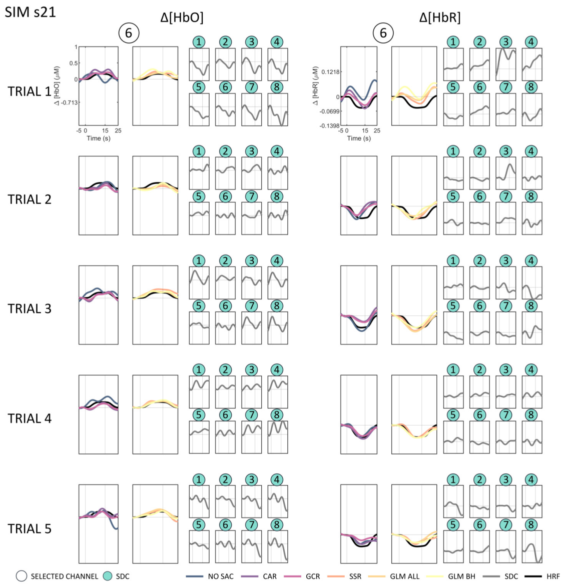
SIM s21
Δ[HbO]
Δ[HbR]
TRIAL 1
TRIAL 2
TRIAL 3
TRIAL 4
TRIAL 5
Time (s)
SELECTED CHANNEL
SDC
NO SAC
CAR
GCR
SSR
GLM ALL
GLM BH
SDC
HRF

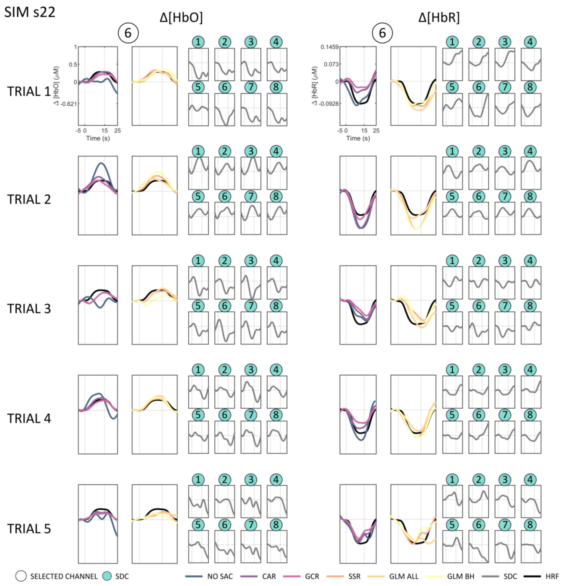
SIM s22
Δ[HbO]
Δ[HbR]
TRIAL 1
TRIAL 2
TRIAL 3
TRIAL 4
TRIAL 5
Time (s)
SELECTED CHANNEL
SDC
NO SAC
CAR
GCR
SSR
GLM ALL
GLM BH
SDC
HRF

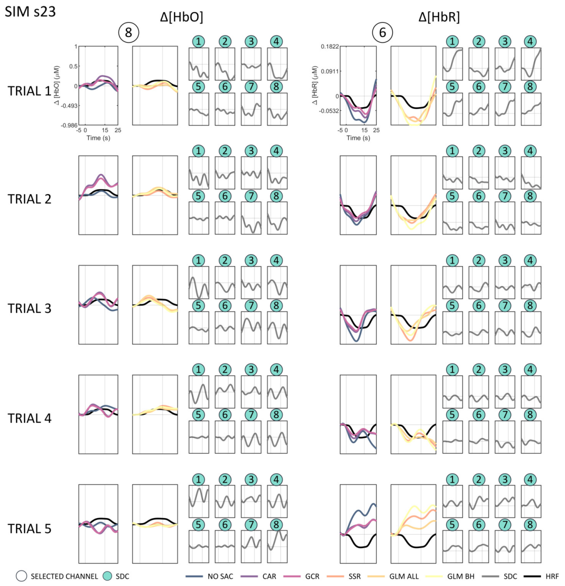
SIM s23
Δ[HbO]
Δ[HbR]
TRIAL 1
TRIAL 2
TRIAL 3
TRIAL 4
TRIAL 5
Time (s)
SELECTED CHANNEL
SDC
NO SAC
CAR
GCR
SSR
GLM ALL
GLM BH
SDC
HRF

ME/MI data

The following figures visualize the normalized single-subject single-trial $\Delta[HbO]$ and $\Delta[HbR]$ data resulting from the real data set of each individually selected channel (based on beta values of GLM ALL corrected data) of all correction methods and all SDCs. Note that only the first five trials are visualized (out of 12 for ME LEFT and ME RIGHT and out of 36 for MI data). As the MI data sets consists of three individual MI tasks (i.e., MI of left hand tapping, MI of right hand tapping and MI of whole body movements) the first five trials always belong to only one of the three tasks and the order of the MI tasks was pseudo-randomized between subjects.

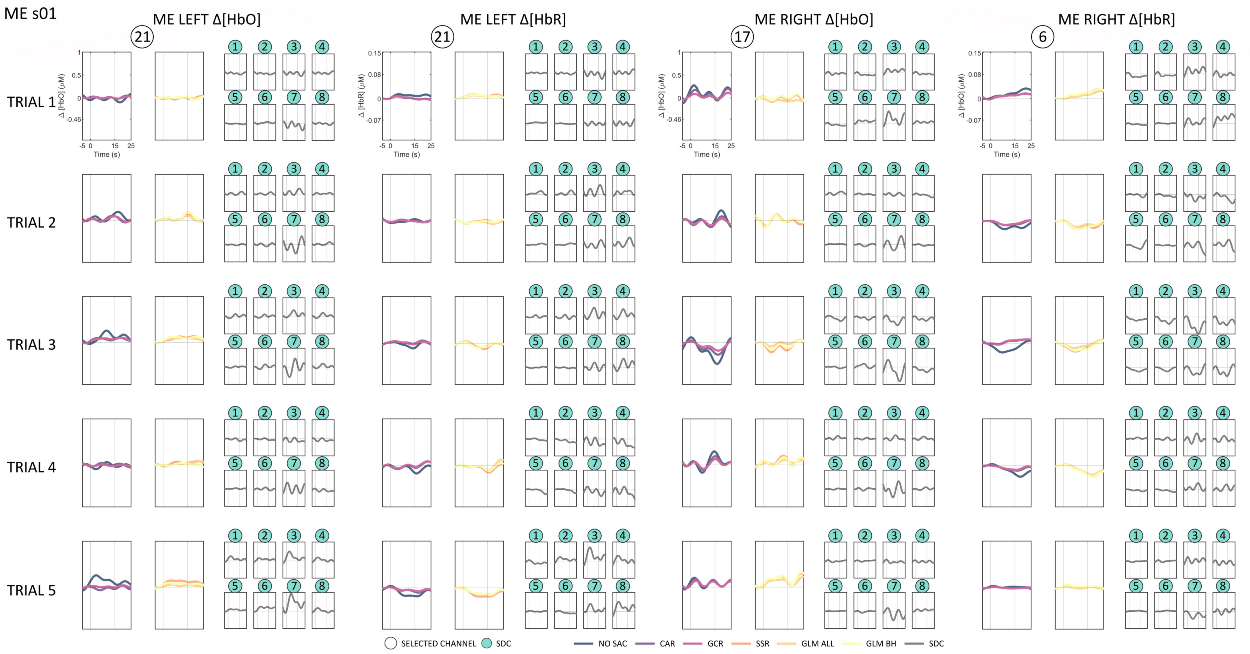
ME s01
ME LEFT Δ[HbO]
ME LEFT Δ[HbR]
ME RIGHT Δ[HbO]
ME RIGHT Δ[HbR]
TRIAL 1
TRIAL 2
TRIAL 3
TRIAL 4
TRIAL 5
Time (s)
SELECTED CHANNEL
SDC
NO SAC
CAR
GCR
SSR
GLM ALL
GLM BH
SDC

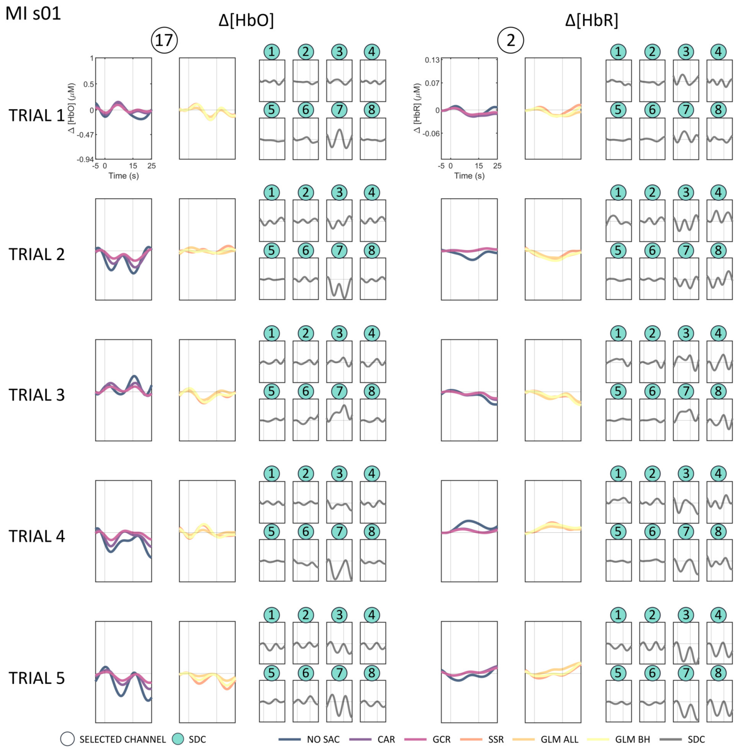
MI s01
Δ[HbO]
Δ[HbR]
TRIAL 1
TRIAL 2
TRIAL 3
TRIAL 4
TRIAL 5
Time (s)
SELECTED CHANNEL
SDC
NO SAC
CAR
GCR
SSR
GLM ALL
GLM BH
SDC

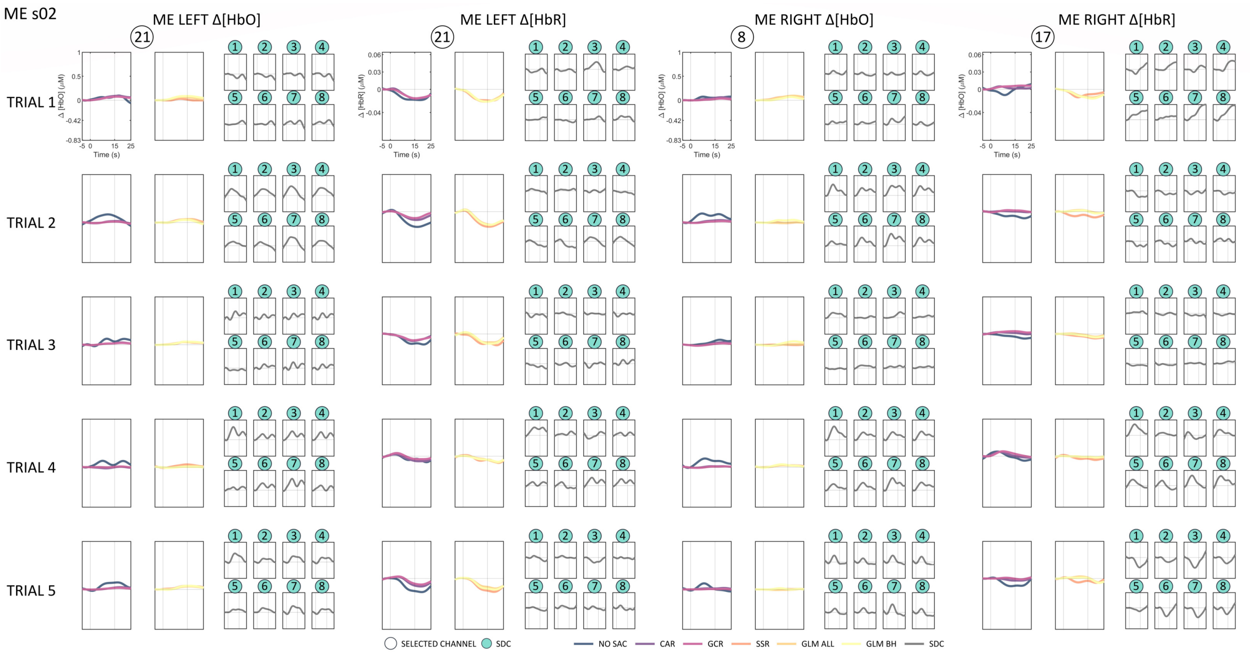
ME s02
ME LEFT Δ[HbO]
ME LEFT Δ[HbR]
ME RIGHT Δ[HbO]
ME RIGHT Δ[HbR]
TRIAL 1
TRIAL 2
TRIAL 3
TRIAL 4
TRIAL 5
Time (s)
SELECTED CHANNEL
SDC
NO SAC
CAR
GCR
SSR
GLM ALL
GLM BH
SDC

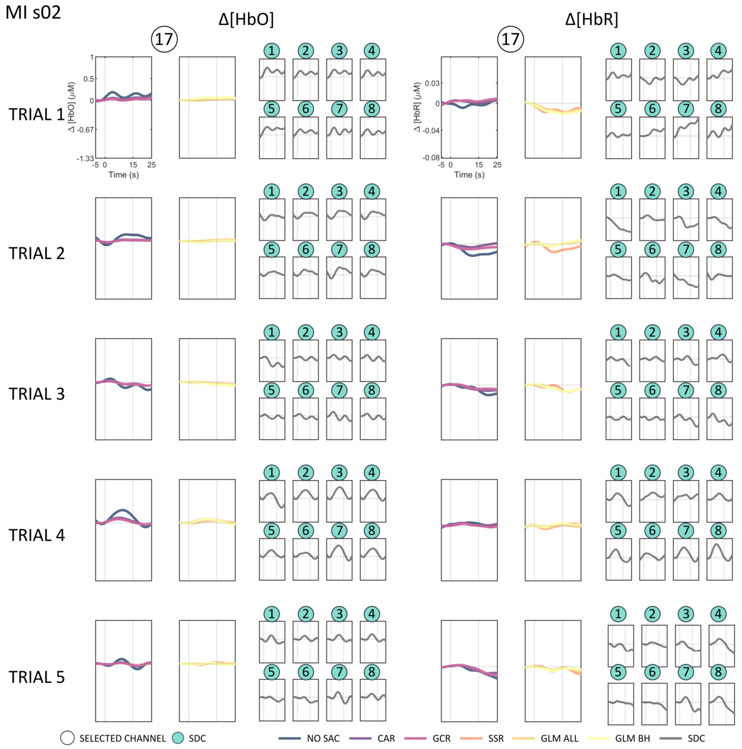
MI s02
Δ[HbO]
Δ[HbR]
TRIAL 1
TRIAL 2
TRIAL 3
TRIAL 4
TRIAL 5
Time (s)
SELECTED CHANNEL
SDC
NO SAC
CAR
GCR
SSR
GLM ALL
GLM BH
SDC

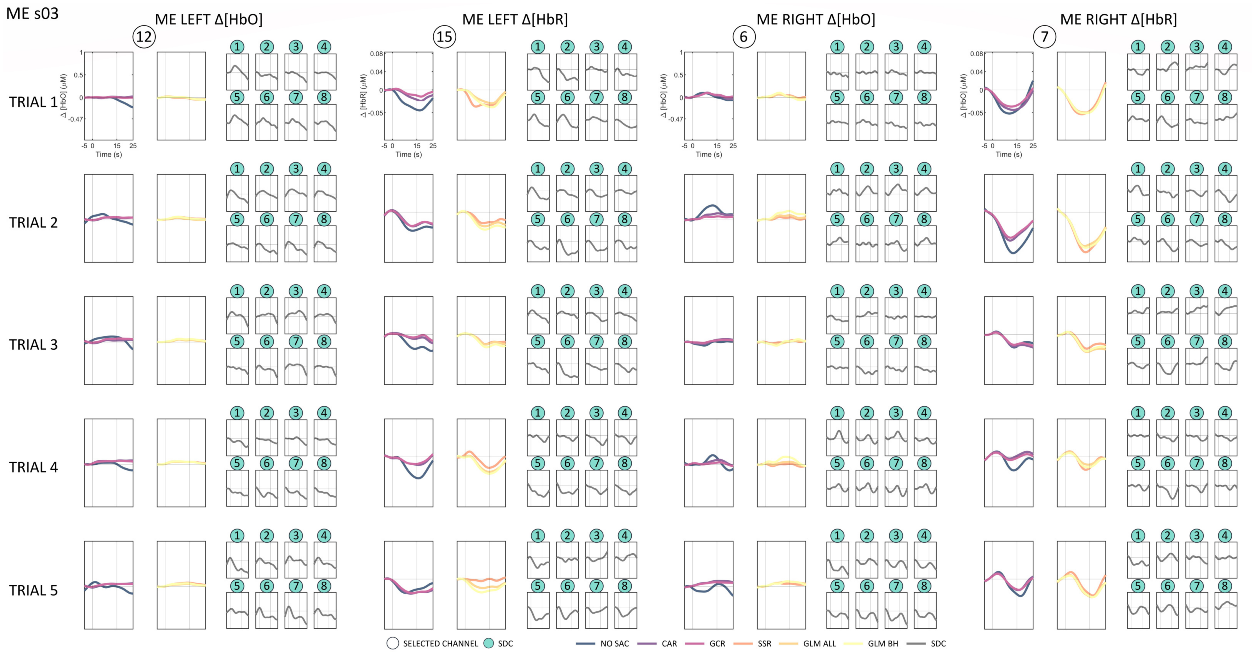
ME s03
ME LEFT Δ[HbO]
ME LEFT Δ[HbR]
ME RIGHT Δ[HbO]
ME RIGHT Δ[HbR]
TRIAL 1
TRIAL 2
TRIAL 3
TRIAL 4
TRIAL 5
Δ [HbO] (μM)
Δ [HbR] (μM)
Time (s)
SELECTED CHANNEL
SDC
NO SAC
CAR
GCR
SSR
GLM ALL
GLM BH
SDC

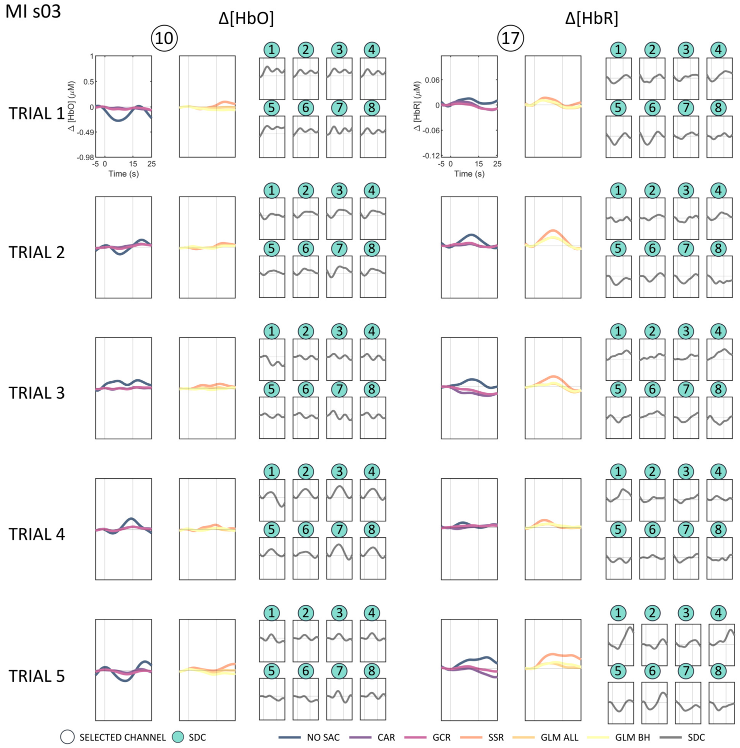
MI s03
Δ[HbO]
Δ[HbR]
TRIAL 1
TRIAL 2
TRIAL 3
TRIAL 4
TRIAL 5
Δ [HbO] (μM)
Δ [HbR] (μM)
Time (s)
SELECTED CHANNEL
SDC
NO SAC
CAR
GCR
SSR
GLM ALL
GLM BH
SDC

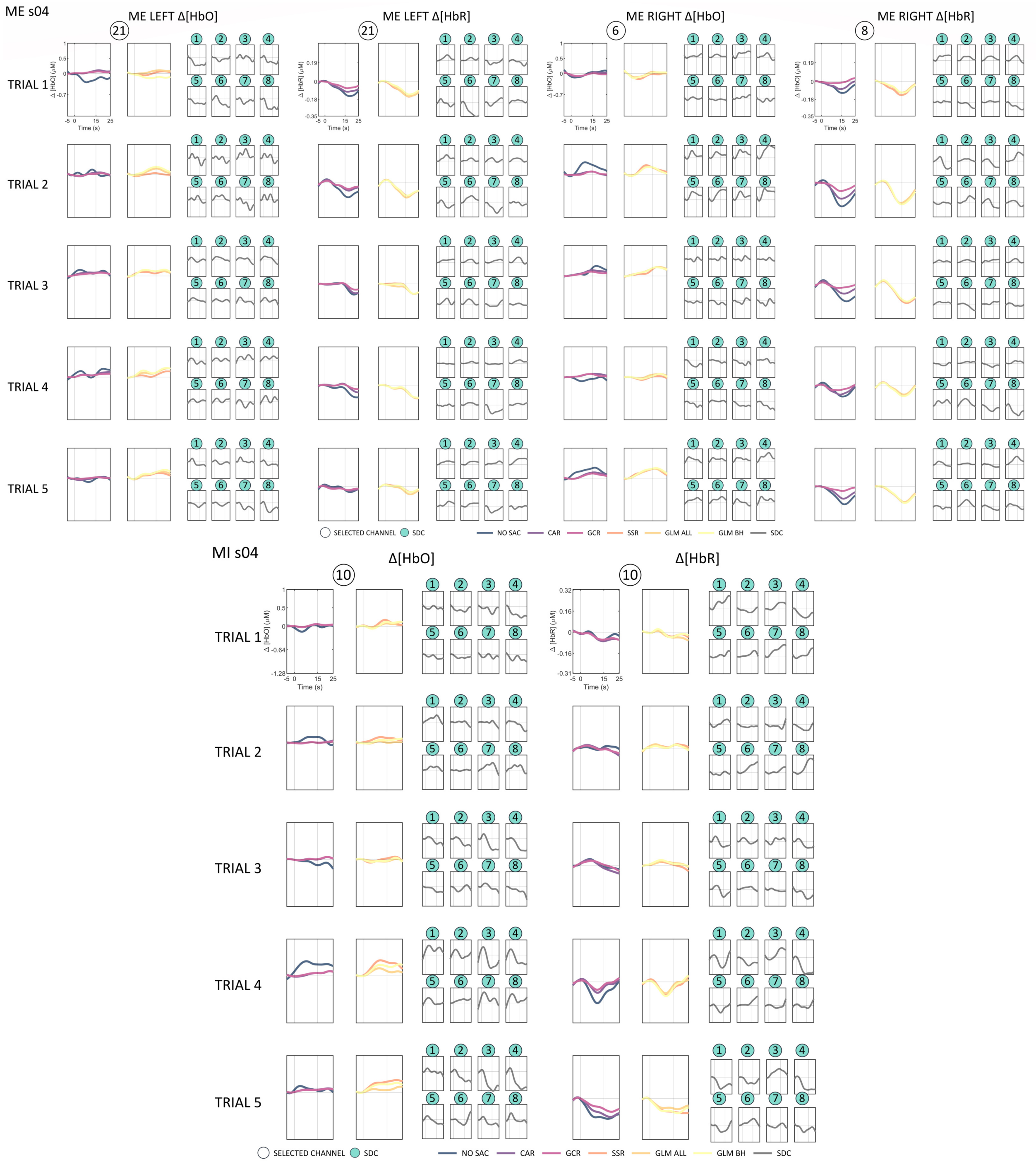
ME s04
ME LEFT Δ[HbO]
ME LEFT Δ[HbR]
ME RIGHT Δ[HbO]
ME RIGHT Δ[HbR]
TRIAL 1
TRIAL 2
TRIAL 3
TRIAL 4
TRIAL 5
Time (s)
SELECTED CHANNEL
SDC
NO SAC
CAR
GCR
SSR
GLM ALL
GLM BH
SDC
MI s04
Δ[HbO]
Δ[HbR]
TRIAL 1
TRIAL 2
TRIAL 3
TRIAL 4
TRIAL 5
Time (s)
SELECTED CHANNEL
SDC
NO SAC
CAR
GCR
SSR
GLM ALL
GLM BH
SDC

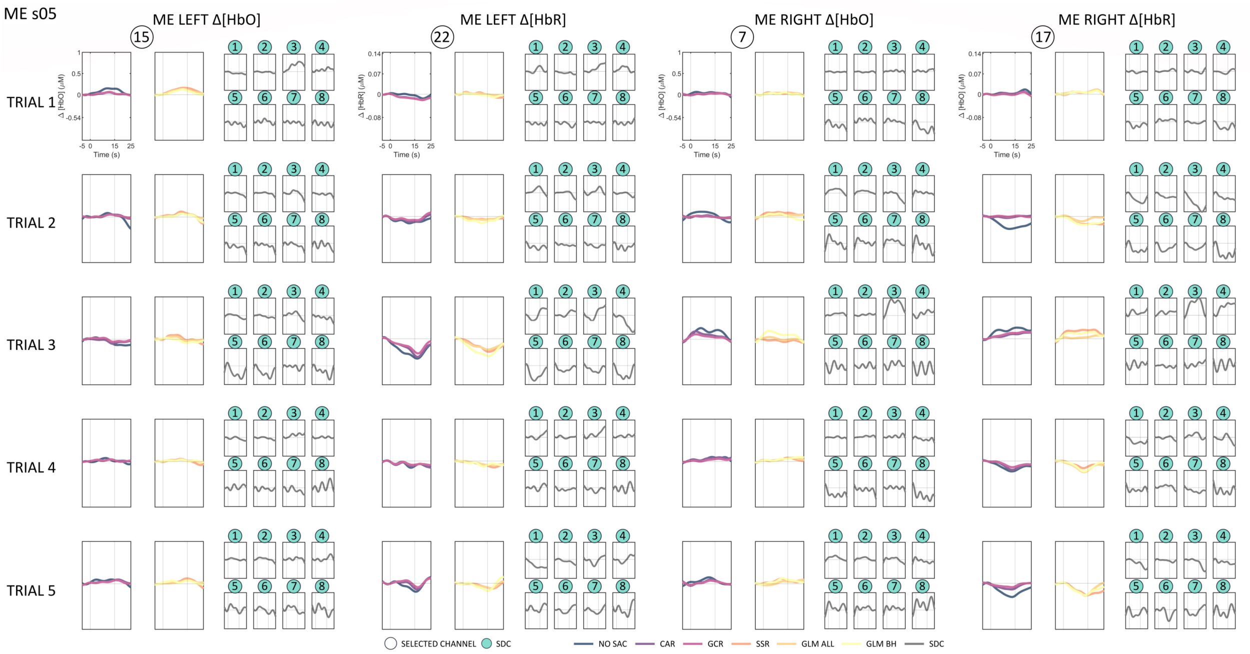
ME s05
ME LEFT Δ[HbO]
ME LEFT Δ[HbR]
ME RIGHT Δ[HbO]
ME RIGHT Δ[HbR]
15
22
7
17
TRIAL 1
TRIAL 2
TRIAL 3
TRIAL 4
TRIAL 5
Δ [HbO] (μM)
Δ [HbR] (μM)
Time (s)
SELECTED CHANNEL
SDC
NO SAC
CAR
GCR
SSR
GLM ALL
GLM BH
SDC

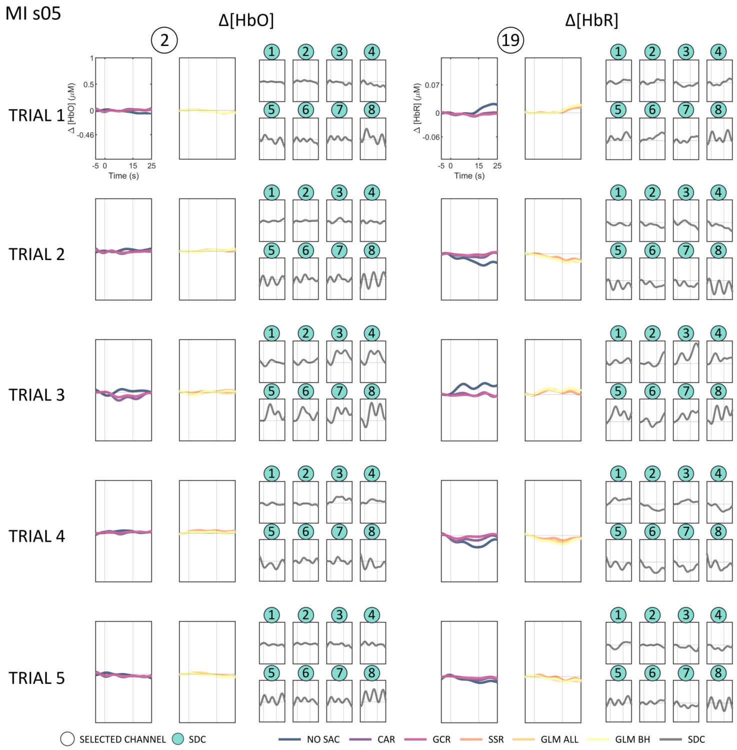
MI s05
Δ[HbO]
Δ[HbR]
2
19
TRIAL 1
TRIAL 2
TRIAL 3
TRIAL 4
TRIAL 5
Δ [HbO] (μM)
Δ [HbR] (μM)
Time (s)
SELECTED CHANNEL
SDC
NO SAC
CAR
GCR
SSR
GLM ALL
GLM BH
SDC

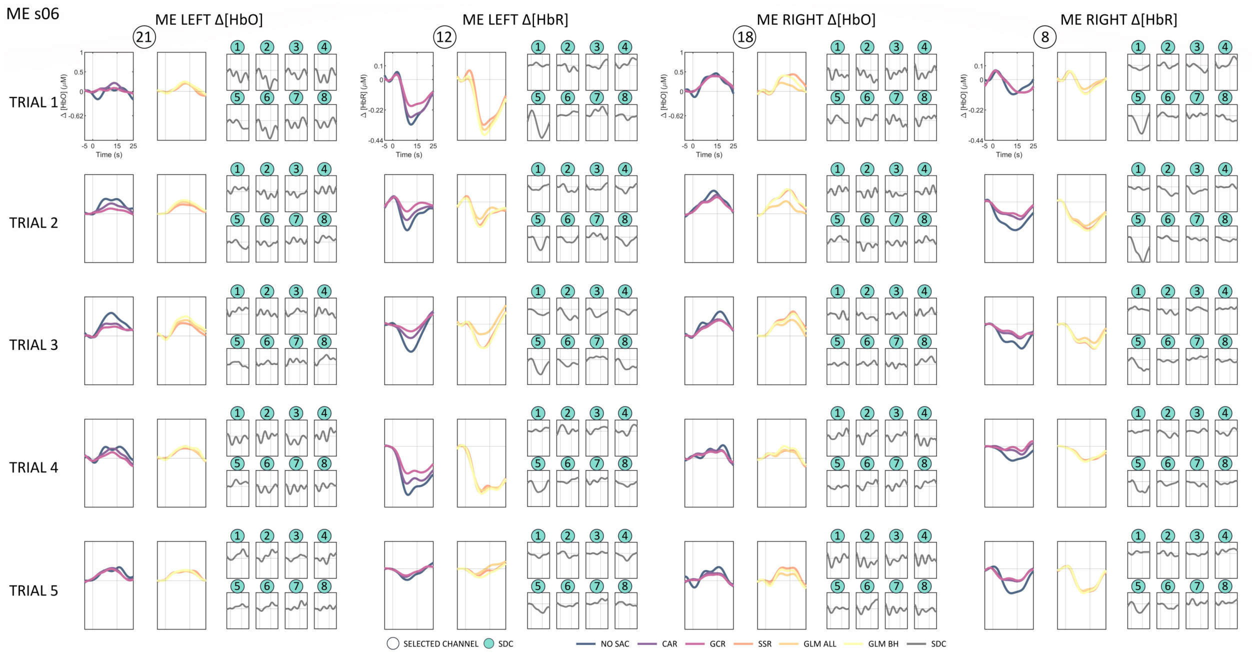
ME s06
ME LEFT Δ[HbO]
ME LEFT Δ[HbR]
ME RIGHT Δ[HbO]
ME RIGHT Δ[HbR]
TRIAL 1
TRIAL 2
TRIAL 3
TRIAL 4
TRIAL 5
Time (s)
SELECTED CHANNEL
SDC
NO SAC
CAR
GCR
SSR
GLM ALL
GLM BH
SDC

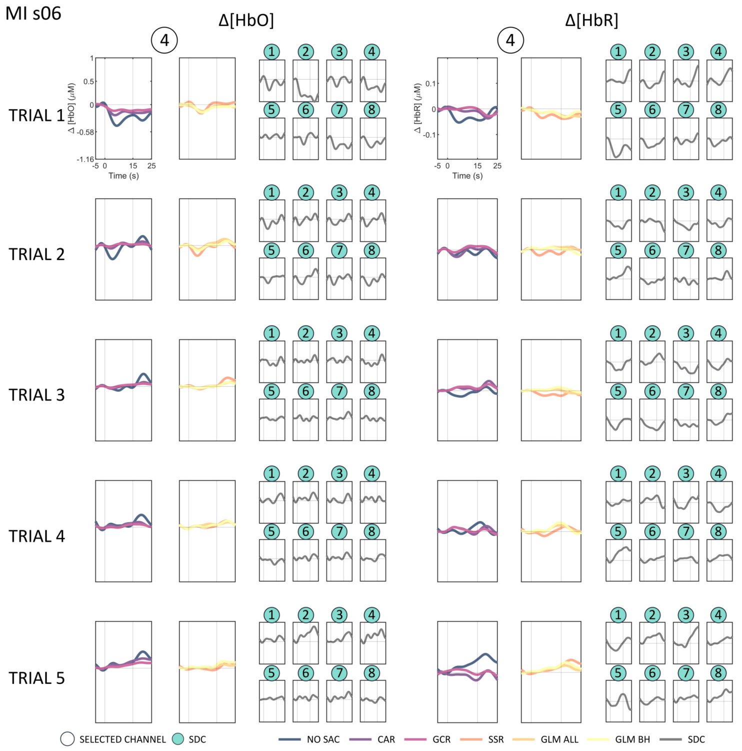
MI s06
Δ[HbO]
Δ[HbR]
TRIAL 1
TRIAL 2
TRIAL 3
TRIAL 4
TRIAL 5
Time (s)
SELECTED CHANNEL
SDC
NO SAC
CAR
GCR
SSR
GLM ALL
GLM BH
SDC

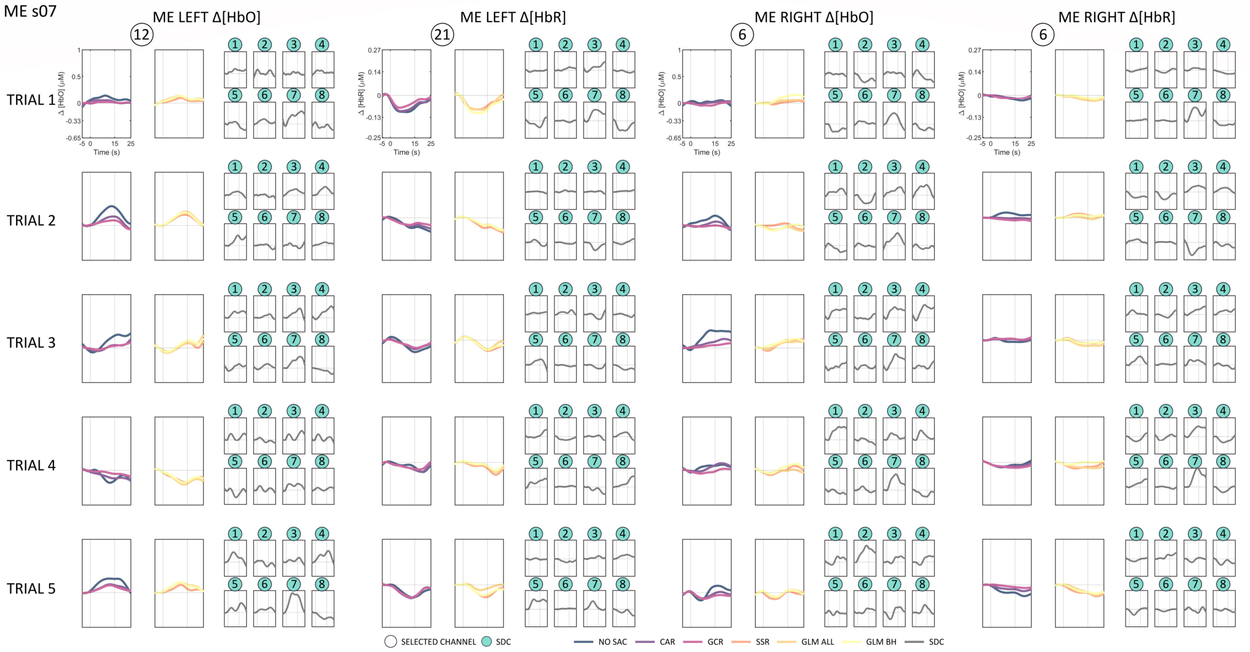
ME s07
ME LEFT Δ[HbO]
ME LEFT Δ[HbR]
ME RIGHT Δ[HbO]
ME RIGHT Δ[HbR]
TRIAL 1
TRIAL 2
TRIAL 3
TRIAL 4
TRIAL 5
Time (s)
SELECTED CHANNEL
SDC
NO SAC
CAR
GCR
SSR
GLM ALL
GLM BH
SDC

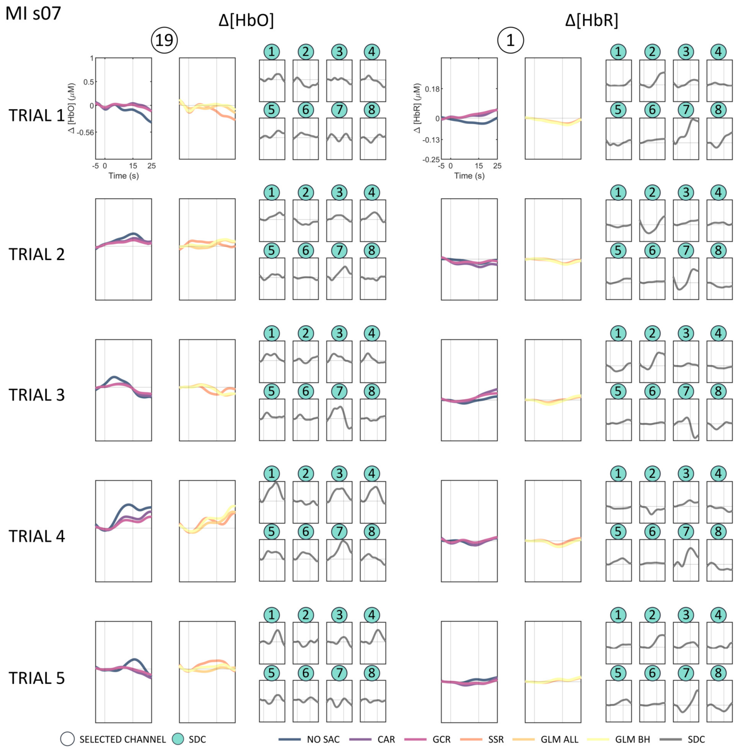
MI s07
Δ[HbO]
Δ[HbR]
TRIAL 1
TRIAL 2
TRIAL 3
TRIAL 4
TRIAL 5
Time (s)
SELECTED CHANNEL
SDC
NO SAC
CAR
GCR
SSR
GLM ALL
GLM BH
SDC

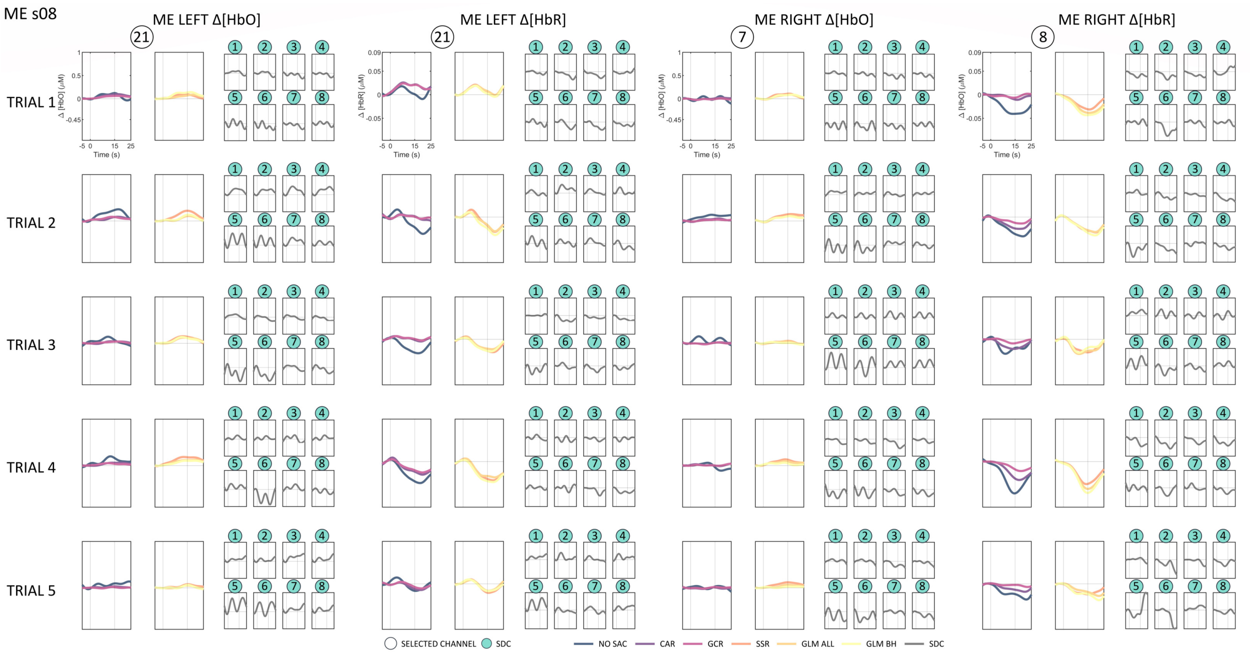
ME s08
ME LEFT Δ[HbO]
ME LEFT Δ[HbR]
ME RIGHT Δ[HbO]
ME RIGHT Δ[HbR]
21
21
7
8
TRIAL 1
TRIAL 2
TRIAL 3
TRIAL 4
TRIAL 5
Δ [HbO] (μM)
Δ [HbR] (μM)
Time (s)
SELECTED CHANNEL
SDC
NO SAC
CAR
GCR
SSR
GLM ALL
GLM BH
SDC

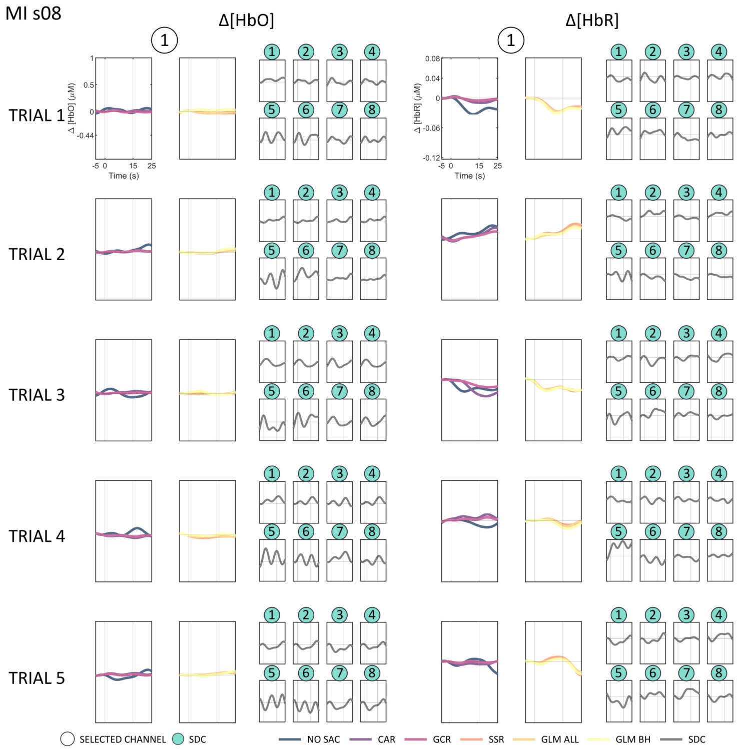
MI s08
Δ[HbO]
Δ[HbR]
1
1
TRIAL 1
TRIAL 2
TRIAL 3
TRIAL 4
TRIAL 5
Δ [HbO] (μM)
Δ [HbR] (μM)
Time (s)
SELECTED CHANNEL
SDC
NO SAC
CAR
GCR
SSR
GLM ALL
GLM BH
SDC

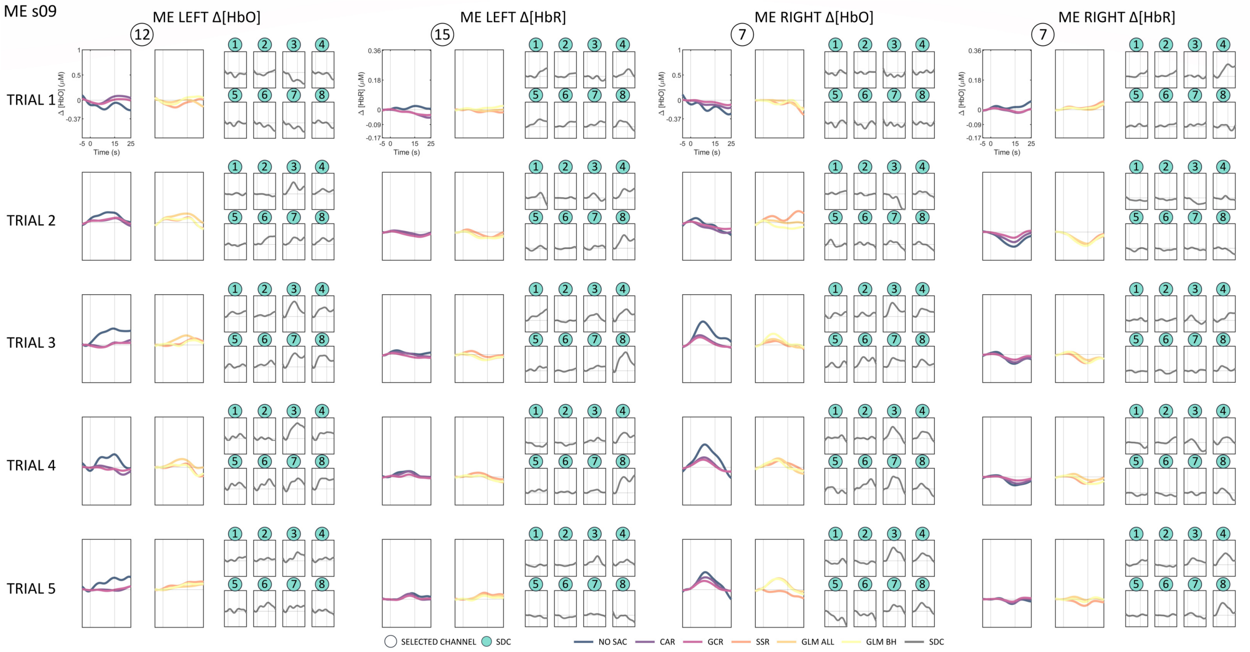
ME s09
ME LEFT Δ[HbO]
ME LEFT Δ[HbR]
ME RIGHT Δ[HbO]
ME RIGHT Δ[HbR]
TRIAL 1
TRIAL 2
TRIAL 3
TRIAL 4
TRIAL 5
Time (s)
SELECTED CHANNEL
SDC
NO SAC
CAR
GCR
SSR
GLM ALL
GLM BH
SDC

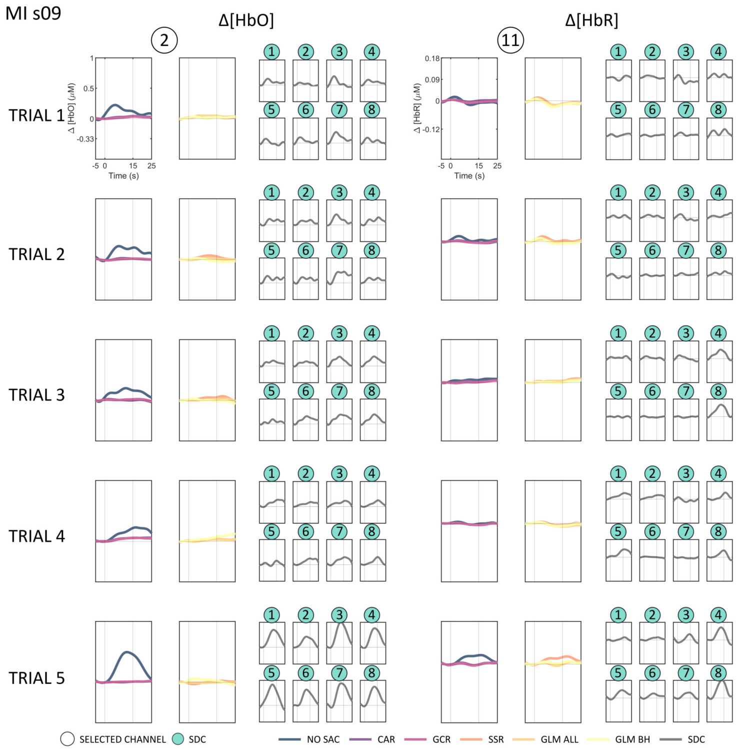
MI s09
Δ[HbO]
Δ[HbR]
TRIAL 1
TRIAL 2
TRIAL 3
TRIAL 4
TRIAL 5
Time (s)
SELECTED CHANNEL
SDC
NO SAC
CAR
GCR
SSR
GLM ALL
GLM BH
SDC

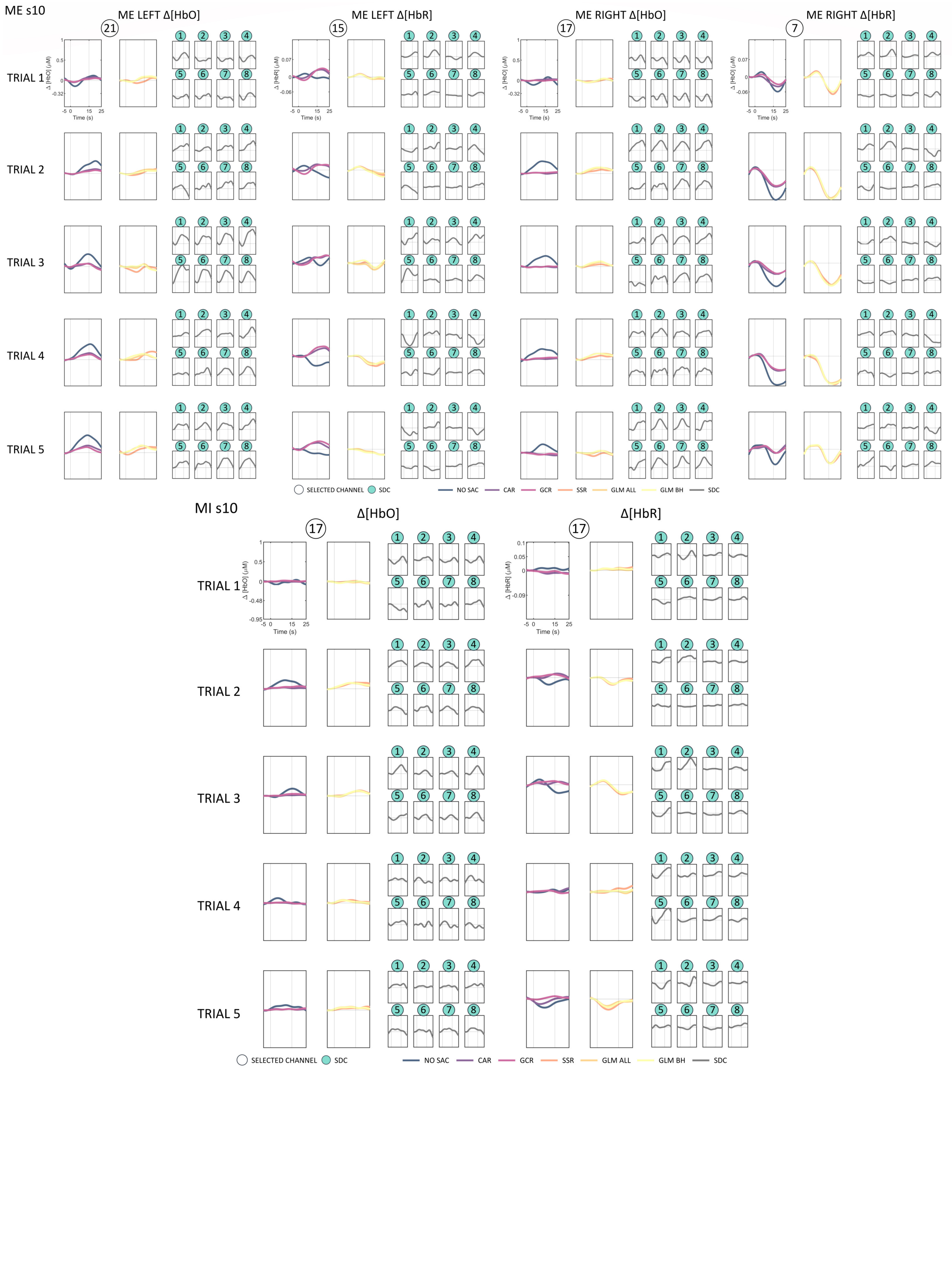
ME s10
ME LEFT Δ[HbO]
ME LEFT Δ[HbR]
ME RIGHT Δ[HbO]
ME RIGHT Δ[HbR]
TRIAL 1
TRIAL 2
TRIAL 3
TRIAL 4
TRIAL 5
SELECTED CHANNEL
SDC
NO SAC
CAR
GCR
SSR
GLM ALL
GLM BH
SDC
MI s10
Δ[HbO]
Δ[HbR]
Time (s)

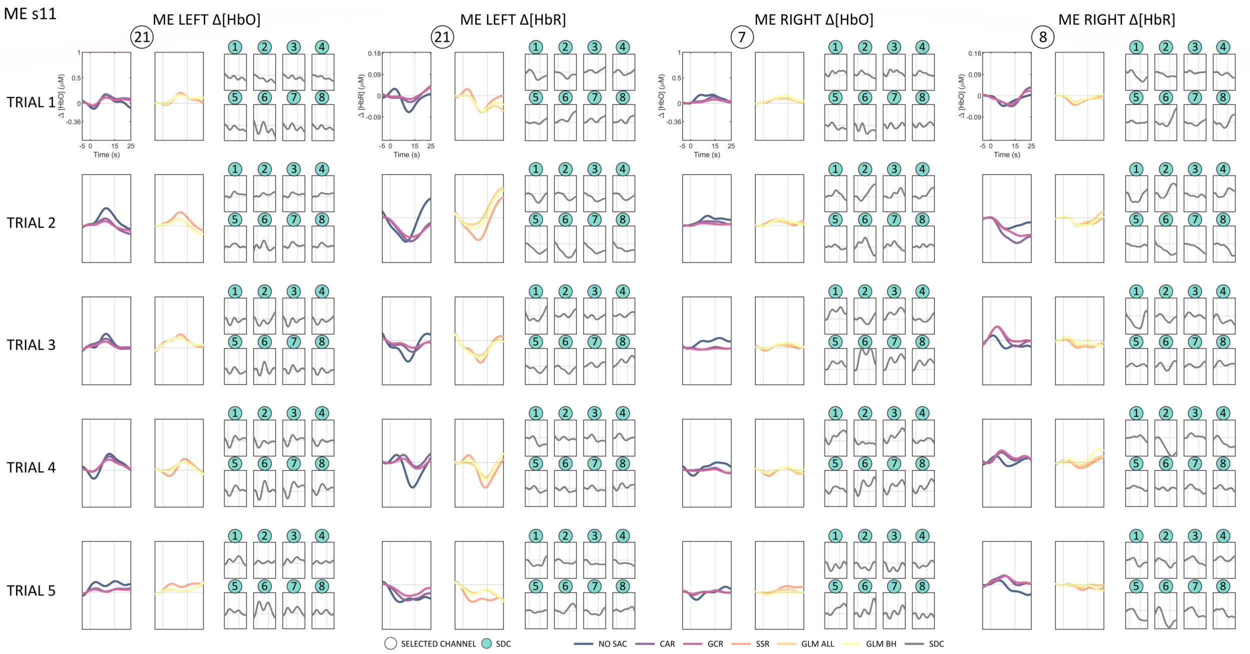
ME s11
ME LEFT Δ[HbO]
ME LEFT Δ[HbR]
ME RIGHT Δ[HbO]
ME RIGHT Δ[HbR]
TRIAL 1
TRIAL 2
TRIAL 3
TRIAL 4
TRIAL 5
Time (s)
SELECTED CHANNEL
SDC
NO SAC
CAR
GCR
SSR
GLM ALL
GLM BH
SDC

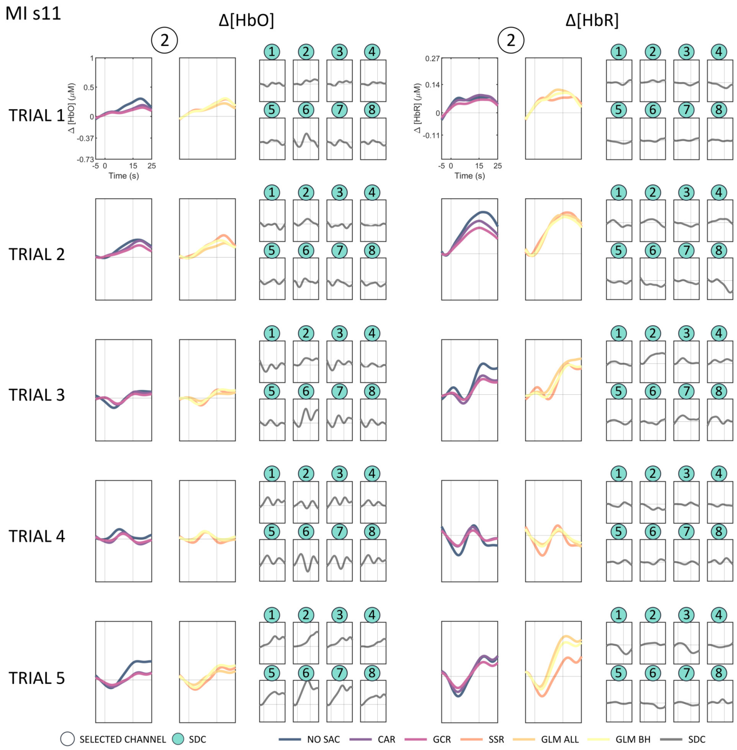
MI s11
Δ[HbO]
Δ[HbR]
TRIAL 1
TRIAL 2
TRIAL 3
TRIAL 4
TRIAL 5
Time (s)
SELECTED CHANNEL
SDC
NO SAC
CAR
GCR
SSR
GLM ALL
GLM BH
SDC

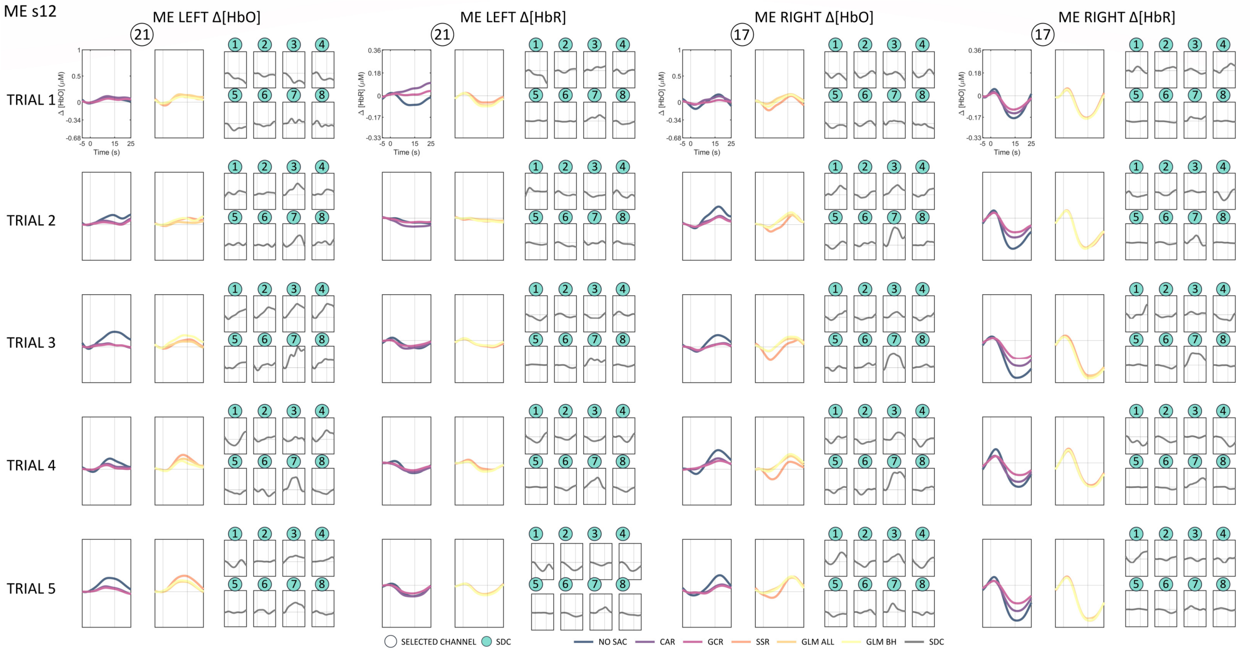
ME s12
ME LEFT Δ[HbO]
ME LEFT Δ[HbR]
ME RIGHT Δ[HbO]
ME RIGHT Δ[HbR]
TRIAL 1
TRIAL 2
TRIAL 3
TRIAL 4
TRIAL 5
SELECTED CHANNEL
SDC
NO SAC
CAR
GCR
SSR
GLM ALL
GLM BH
SDC

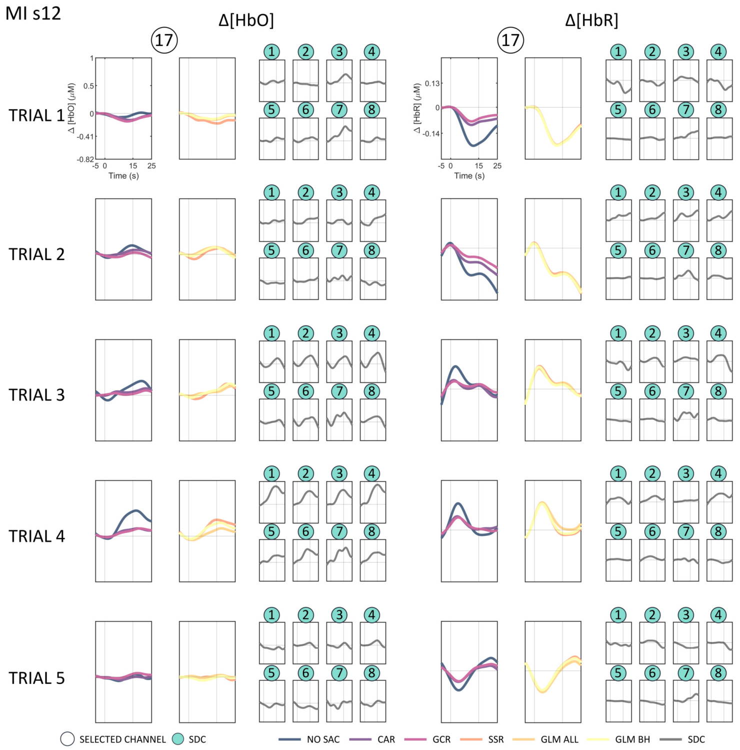
MI s12
Δ[HbO]
Δ[HbR]
TRIAL 1
TRIAL 2
TRIAL 3
TRIAL 4
TRIAL 5
SELECTED CHANNEL
SDC
NO SAC
CAR
GCR
SSR
GLM ALL
GLM BH
SDC

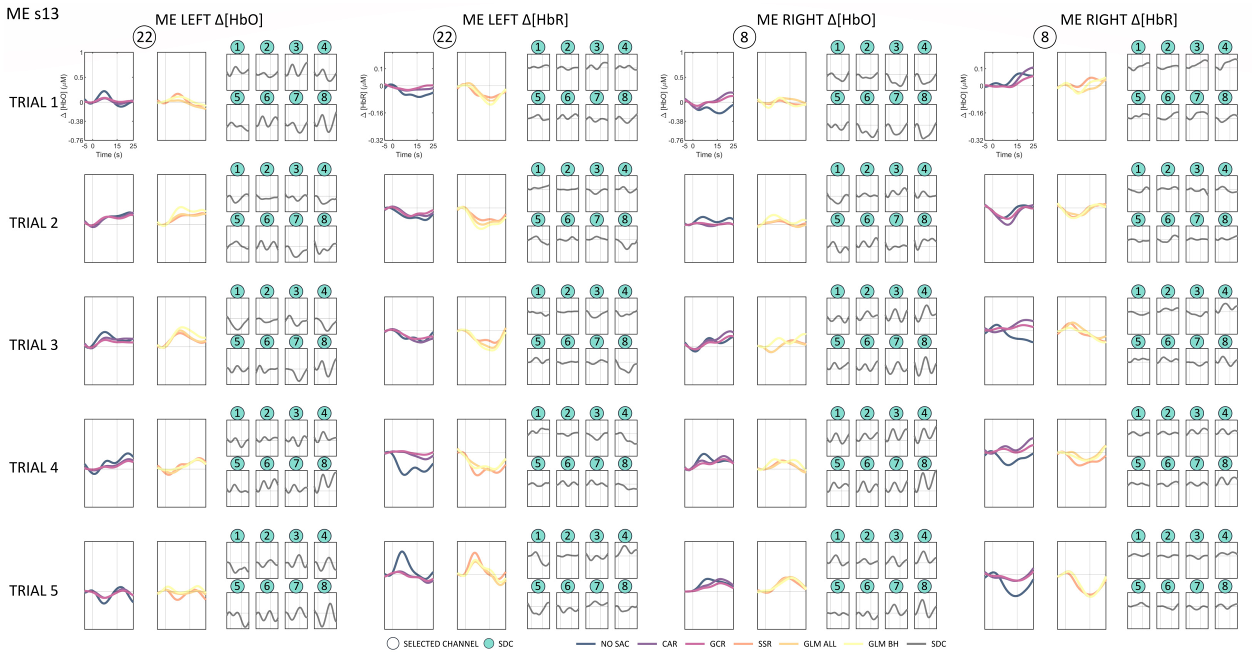
ME s13
ME LEFT Δ[HbO]
ME LEFT Δ[HbR]
ME RIGHT Δ[HbO]
ME RIGHT Δ[HbR]
TRIAL 1
TRIAL 2
TRIAL 3
TRIAL 4
TRIAL 5
SELECTED CHANNEL
SDC
NO SAC
CAR
GCR
SSR
GLM ALL
GLM BH
SDC

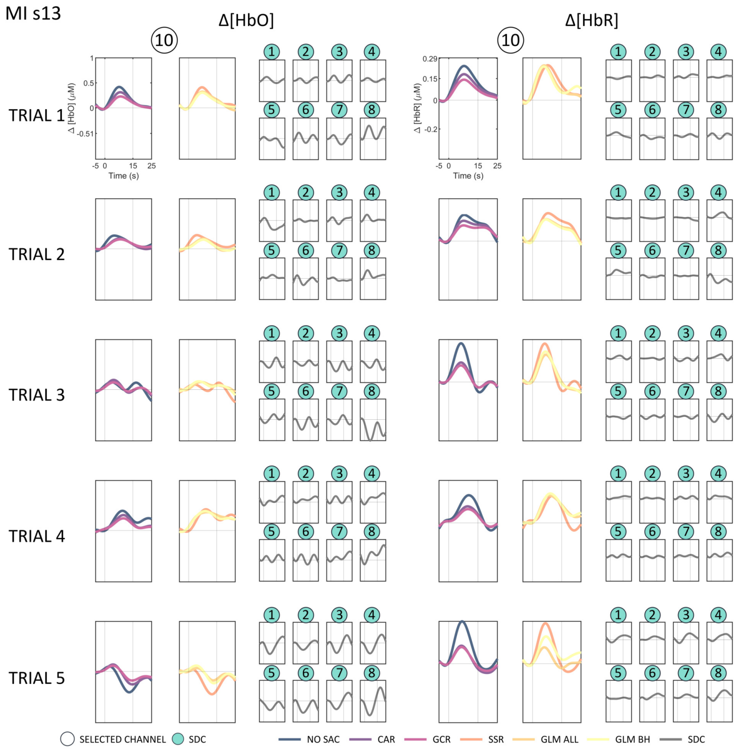
MI s13
Δ[HbO]
Δ[HbR]
TRIAL 1
TRIAL 2
TRIAL 3
TRIAL 4
TRIAL 5
SELECTED CHANNEL
SDC
NO SAC
CAR
GCR
SSR
GLM ALL
GLM BH
SDC

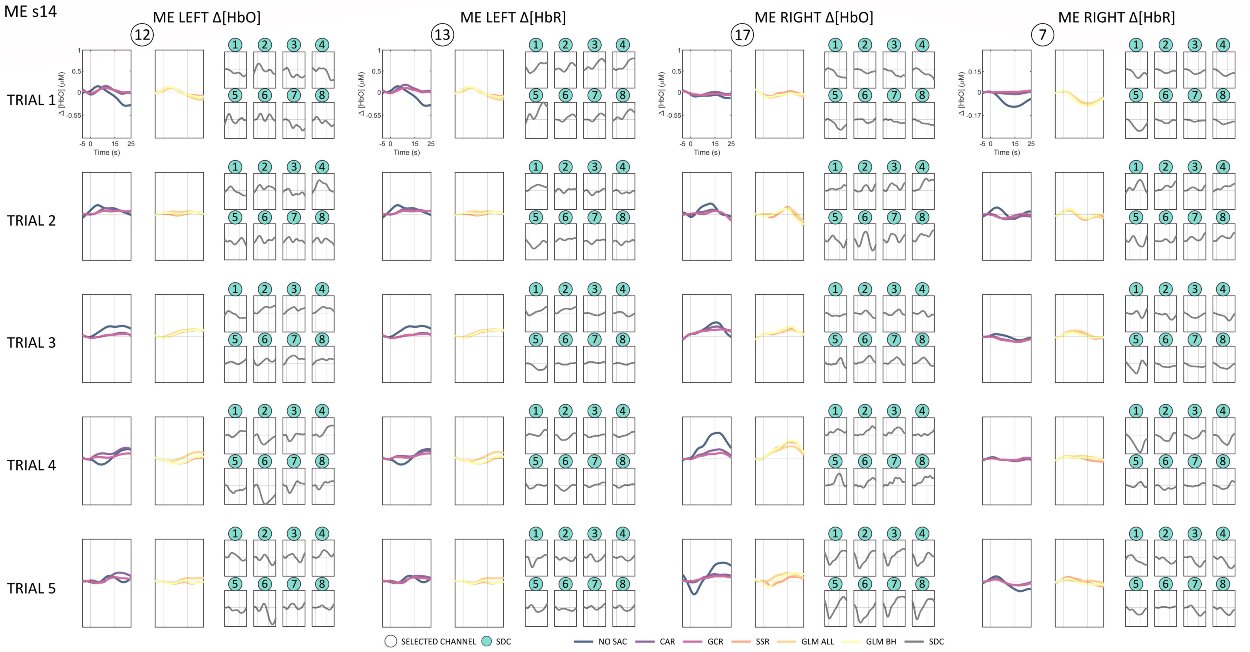
ME s14
ME LEFT Δ[HbO]
ME LEFT Δ[HbR]
ME RIGHT Δ[HbO]
ME RIGHT Δ[HbR]
TRIAL 1
TRIAL 2
TRIAL 3
TRIAL 4
TRIAL 5
Δ [HbO] (μM)
Time (s)
SELECTED CHANNEL
SDC
NO SAC
CAR
GCR
SSR
GLM ALL
GLM BH
SDC

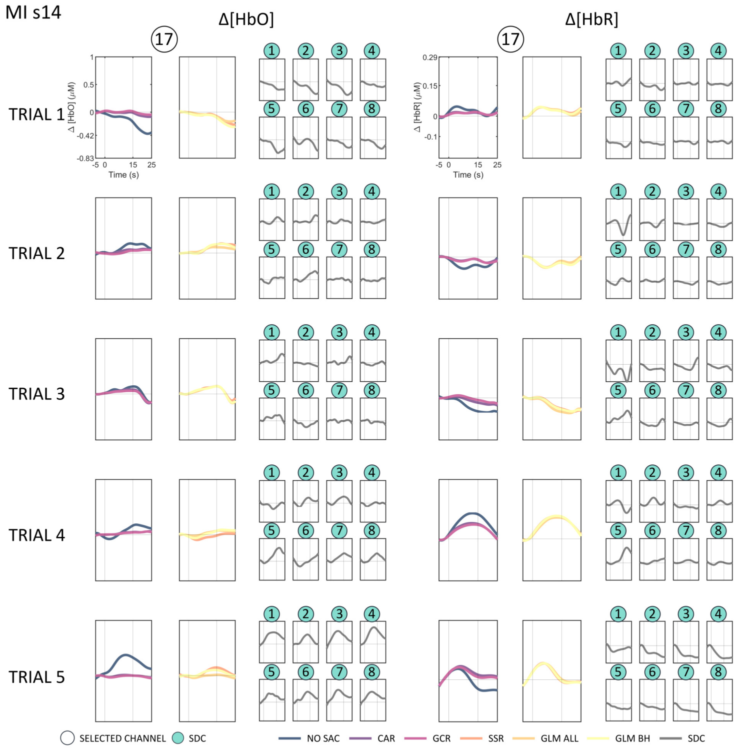
MI s14
Δ[HbO]
Δ[HbR]
TRIAL 1
TRIAL 2
TRIAL 3
TRIAL 4
TRIAL 5
Δ [HbO] (μM)
Δ [HbR] (μM)
Time (s)
SELECTED CHANNEL
SDC
NO SAC
CAR
GCR
SSR
GLM ALL
GLM BH
SDC

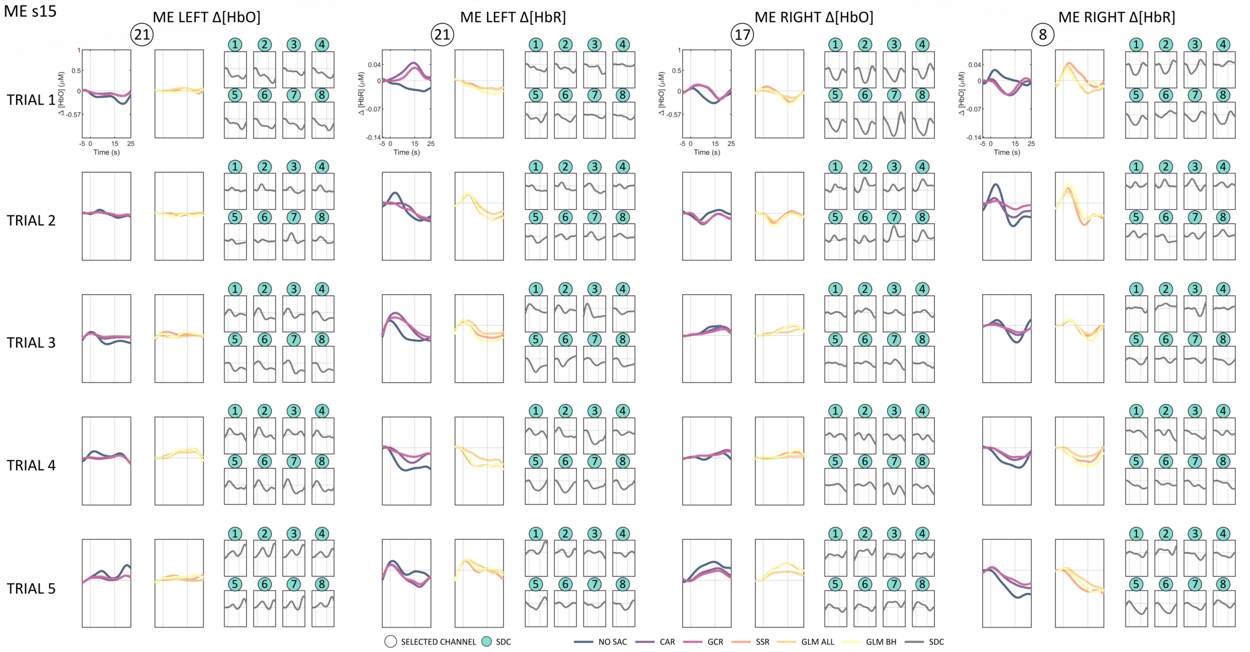
ME s15
ME LEFT Δ[HbO]
ME LEFT Δ[HbR]
ME RIGHT Δ[HbO]
ME RIGHT Δ[HbR]
TRIAL 1
TRIAL 2
TRIAL 3
TRIAL 4
TRIAL 5
Time (s)
SELECTED CHANNEL
SDC
NO SAC
CAR
GCR
SSR
GLM ALL
GLM BH
SDC

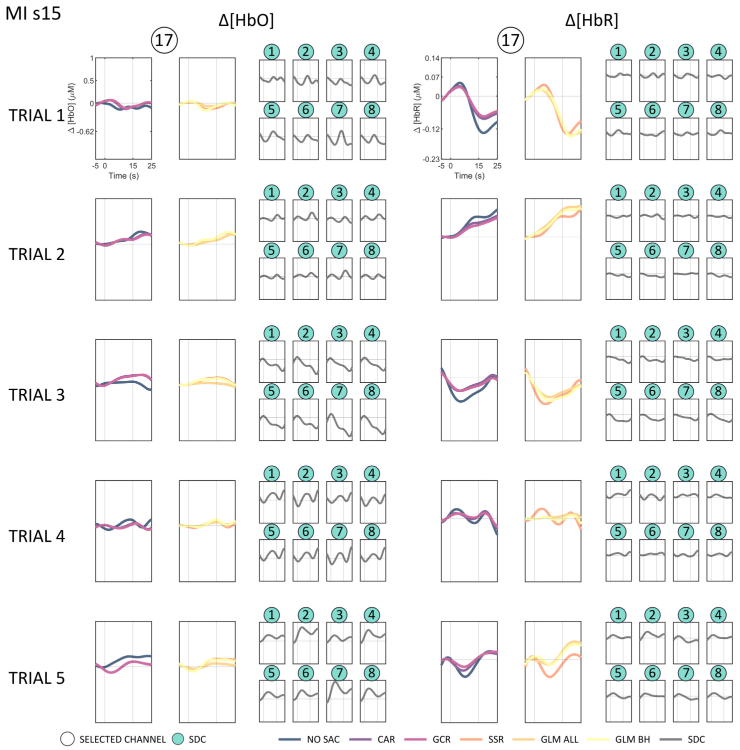
MI s15
Δ[HbO]
Δ[HbR]
TRIAL 1
TRIAL 2
TRIAL 3
TRIAL 4
TRIAL 5
Time (s)
SELECTED CHANNEL
SDC
NO SAC
CAR
GCR
SSR
GLM ALL
GLM BH
SDC

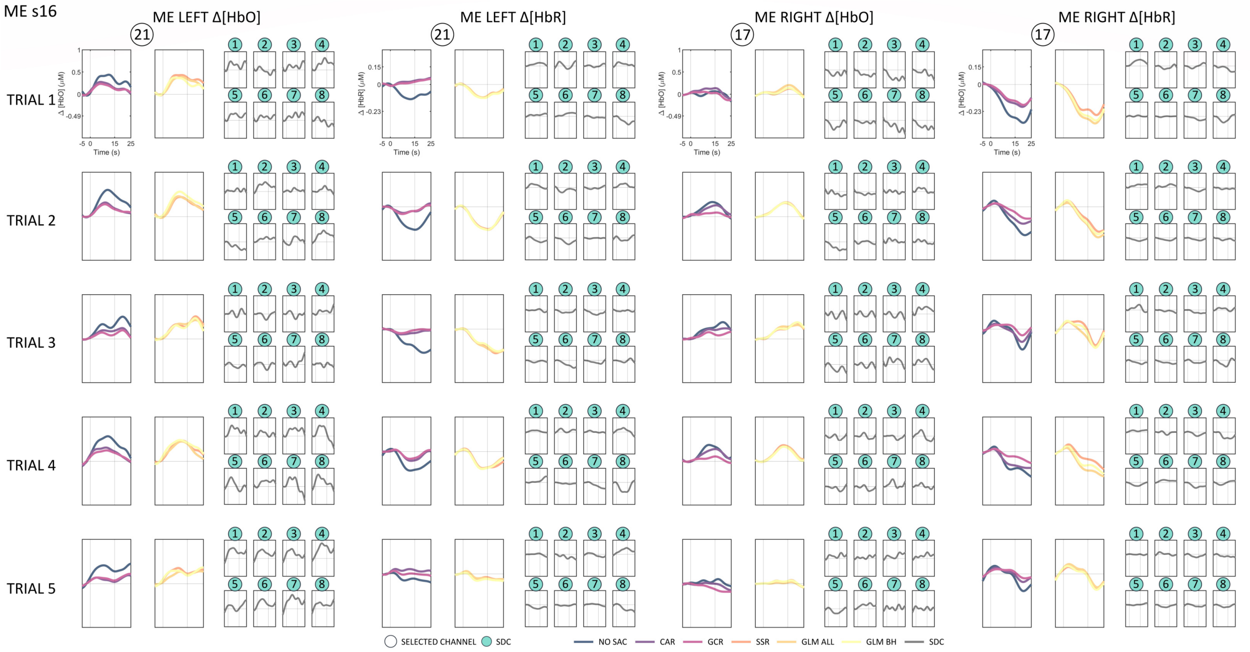

ME s16
ME LEFT Δ[HbO]
ME LEFT Δ[HbR]
ME RIGHT Δ[HbO]
ME RIGHT Δ[HbR]
TRIAL 1
TRIAL 2
TRIAL 3
TRIAL 4
TRIAL 5
Time (s)
SELECTED CHANNEL
SDC
NO SAC
CAR
GCR
SSR
GLM ALL
GLM BH
SDC

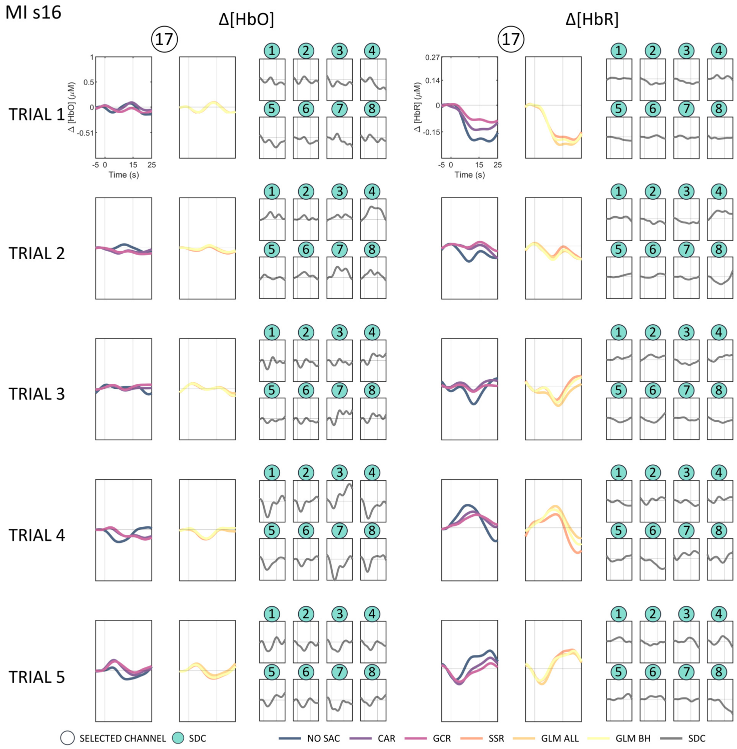

MI s16
Δ[HbO]
Δ[HbR]
TRIAL 1
TRIAL 2
TRIAL 3
TRIAL 4
TRIAL 5
Time (s)
SELECTED CHANNEL
SDC
NO SAC
CAR
GCR
SSR
GLM ALL
GLM BH
SDC

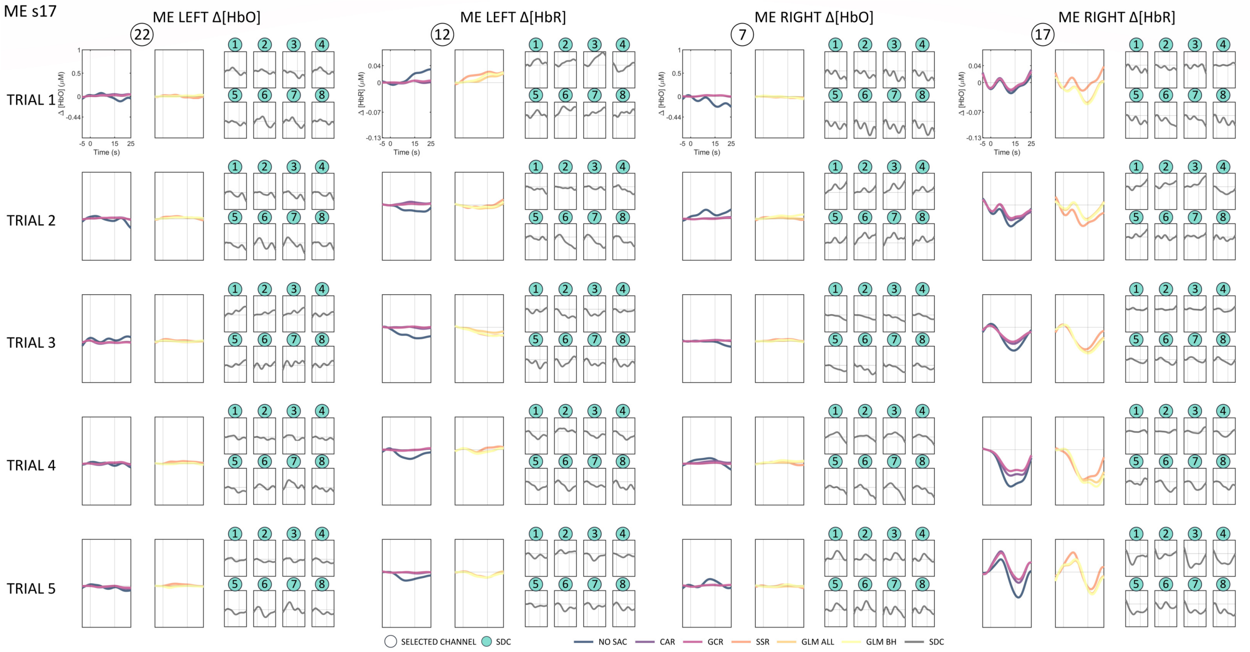
ME s17
ME LEFT Δ[HbO]
ME LEFT Δ[HbR]
ME RIGHT Δ[HbO]
ME RIGHT Δ[HbR]
22
12
7
17
TRIAL 1
TRIAL 2
TRIAL 3
TRIAL 4
TRIAL 5
Time (s)
SELECTED CHANNEL
SDC
NO SAC
CAR
GCR
SSR
GLM ALL
GLM BH
SDC

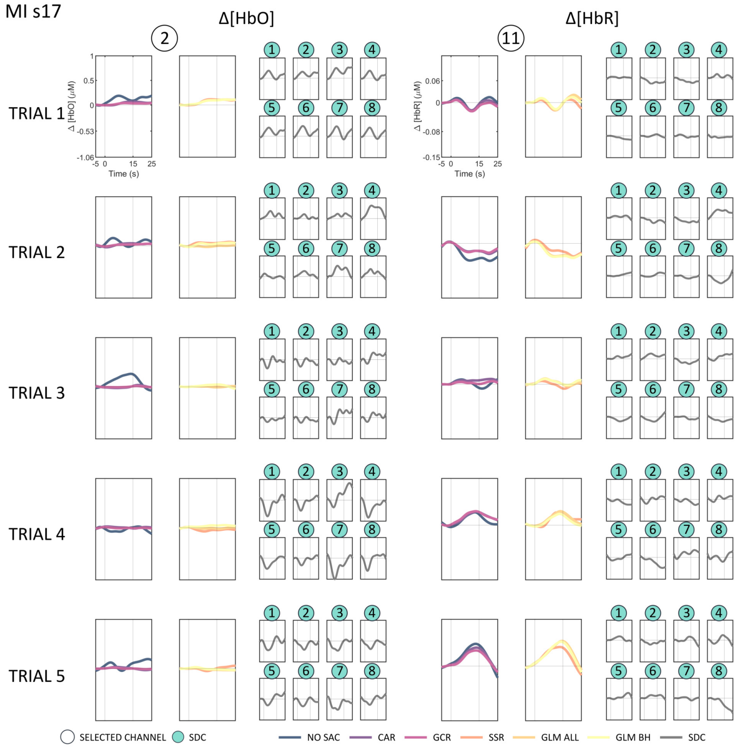
MI s17
Δ[HbO]
Δ[HbR]
2
11
TRIAL 1
TRIAL 2
TRIAL 3
TRIAL 4
TRIAL 5
Time (s)
SELECTED CHANNEL
SDC
NO SAC
CAR
GCR
SSR
GLM ALL
GLM BH
SDC

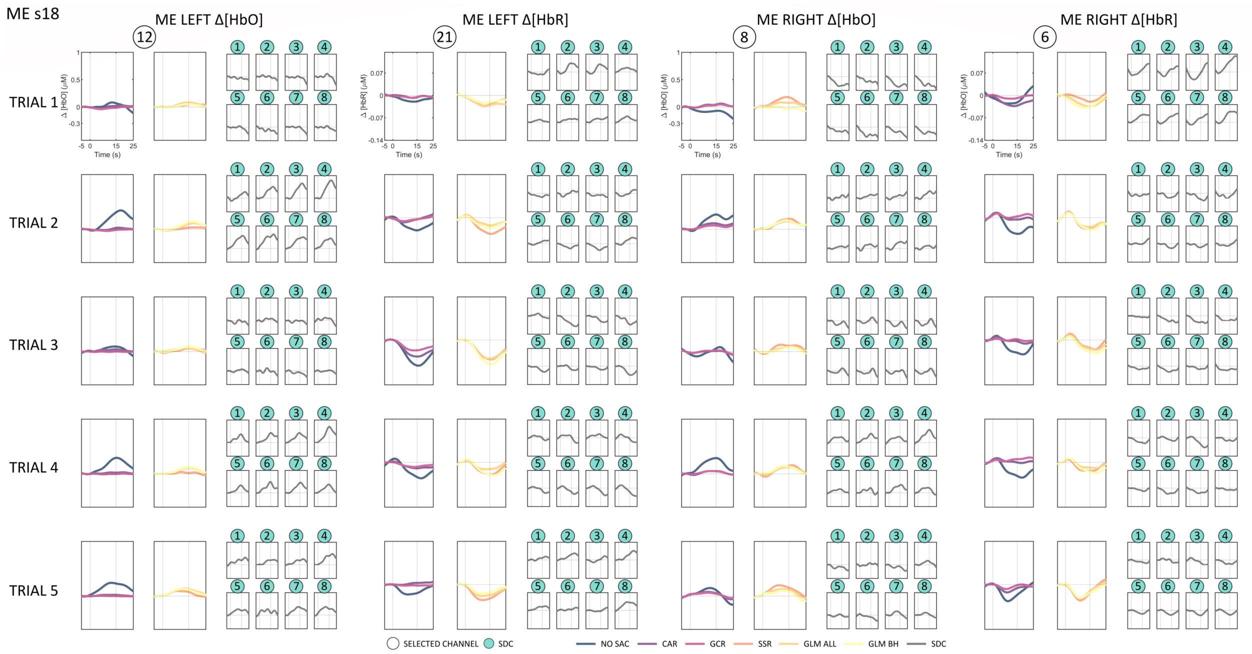
ME s18
ME LEFT Δ[HbO]
ME LEFT Δ[HbR]
ME RIGHT Δ[HbO]
ME RIGHT Δ[HbR]
TRIAL 1
TRIAL 2
TRIAL 3
TRIAL 4
TRIAL 5
Time (s)
SELECTED CHANNEL
SDC
NO SAC
CAR
GCR
SSR
GLM ALL
GLM BH
SDC

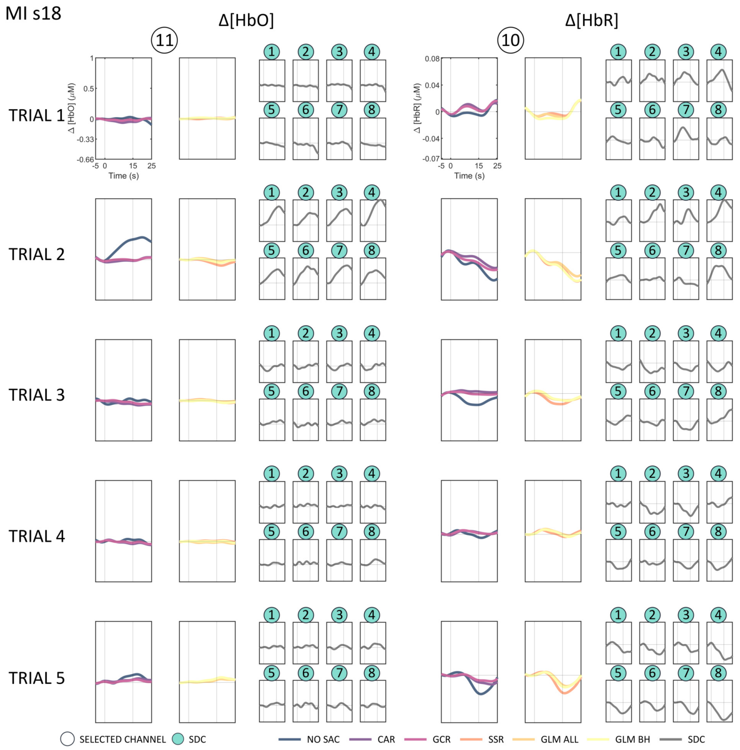
MI s18
Δ[HbO]
Δ[HbR]
TRIAL 1
TRIAL 2
TRIAL 3
TRIAL 4
TRIAL 5
Time (s)
SELECTED CHANNEL
SDC
NO SAC
CAR
GCR
SSR
GLM ALL
GLM BH
SDC

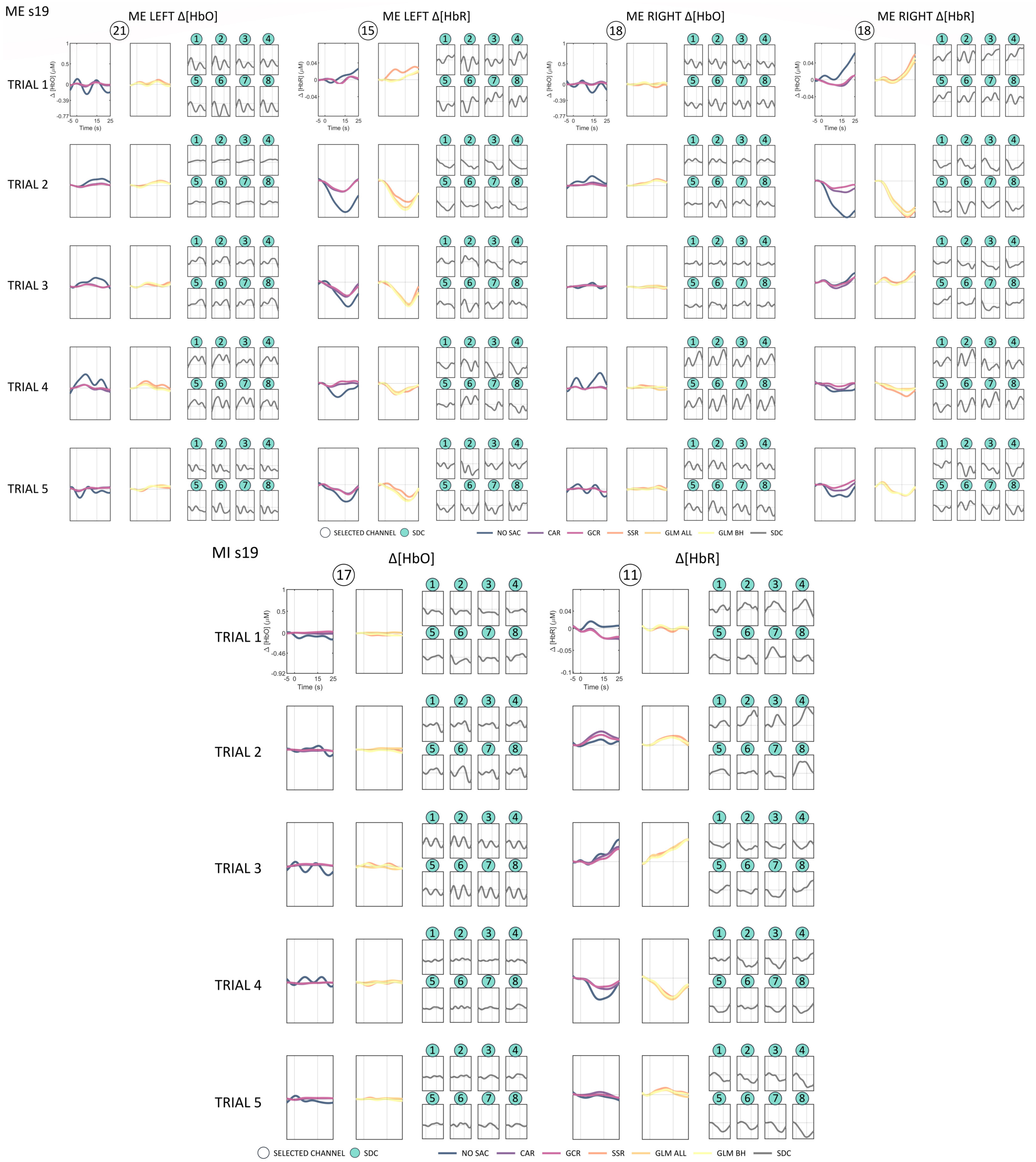
ME s19
ME LEFT Δ[HbO]
ME LEFT Δ[HbR]
ME RIGHT Δ[HbO]
ME RIGHT Δ[HbR]
TRIAL 1
TRIAL 2
TRIAL 3
TRIAL 4
TRIAL 5
Time (s)
SELECTED CHANNEL
SDC
NO SAC
CAR
GCR
SSR
GLM ALL
GLM BH
SDC
MI s19
Δ[HbO]
Δ[HbR]
TRIAL 1
TRIAL 2
TRIAL 3
TRIAL 4
TRIAL 5
Time (s)
SELECTED CHANNEL
SDC
NO SAC
CAR
GCR
SSR
GLM ALL
GLM BH
SDC

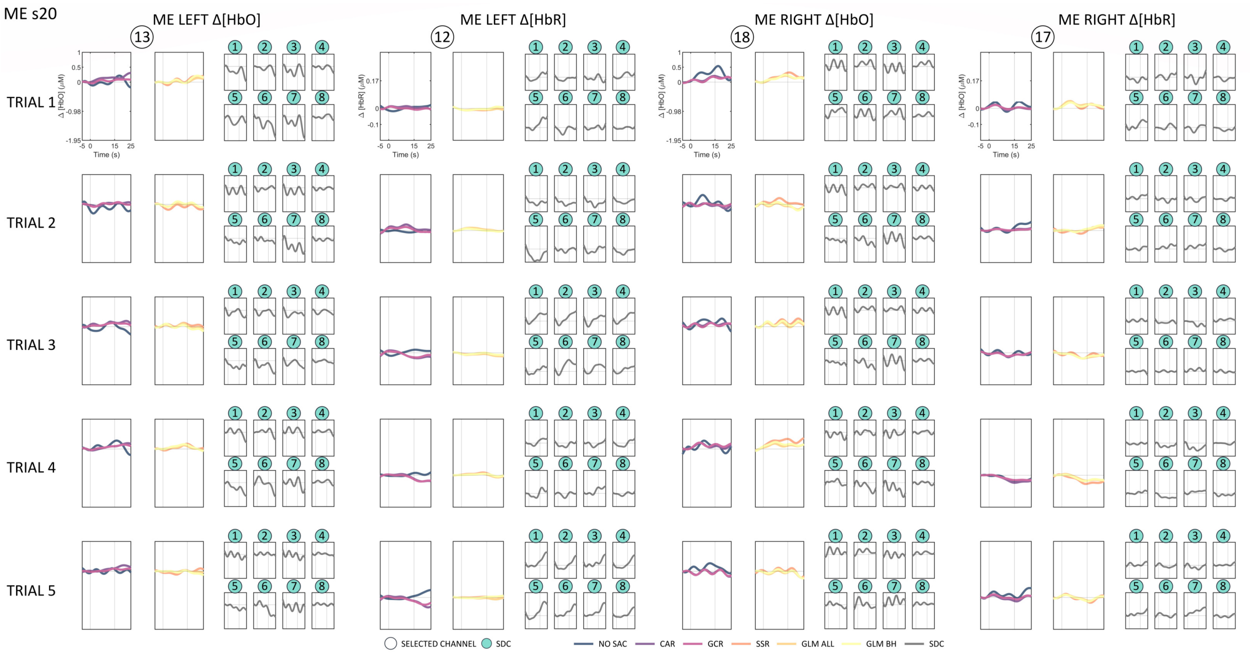
ME s20
ME LEFT Δ[HbO]
ME LEFT Δ[HbR]
ME RIGHT Δ[HbO]
ME RIGHT Δ[HbR]
13
12
18
17
TRIAL 1
TRIAL 2
TRIAL 3
TRIAL 4
TRIAL 5
Δ [HbO] (μM)
Δ [HbR] (μM)
Time (s)
SELECTED CHANNEL
SDC
NO SAC
CAR
GCR
SSR
GLM ALL
GLM BH
SDC

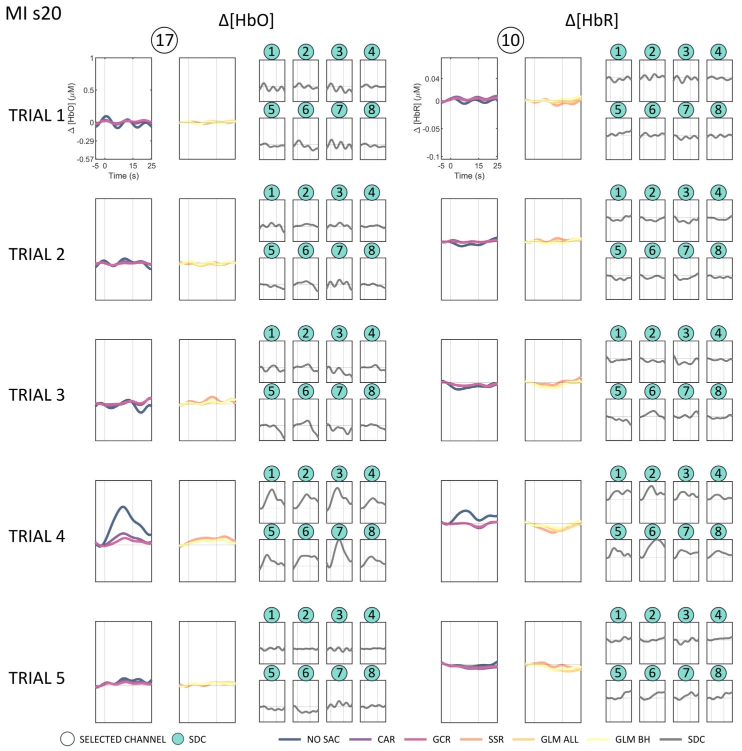
MI s20
Δ[HbO]
Δ[HbR]
17
10
TRIAL 1
TRIAL 2
TRIAL 3
TRIAL 4
TRIAL 5
Δ [HbO] (μM)
Δ [HbR] (μM)
Time (s)
SELECTED CHANNEL
SDC
NO SAC
CAR
GCR
SSR
GLM ALL
GLM BH
SDC

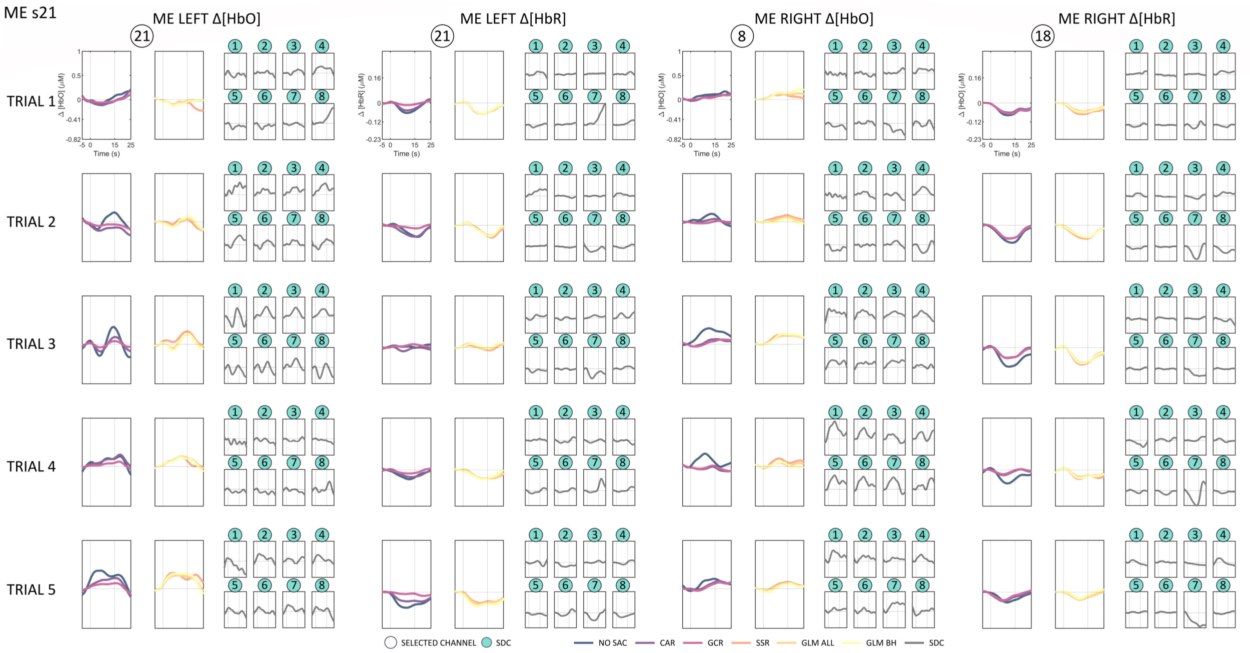
ME s21
ME LEFT Δ[HbO]
ME LEFT Δ[HbR]
ME RIGHT Δ[HbO]
ME RIGHT Δ[HbR]
21
21
8
18
TRIAL 1
TRIAL 2
TRIAL 3
TRIAL 4
TRIAL 5
Δ [HbO] (μM)
Δ [HbR] (μM)
Time (s)
SELECTED CHANNEL
SDC
NO SAC
CAR
GCR
SSR
GLM ALL
GLM BH
SDC

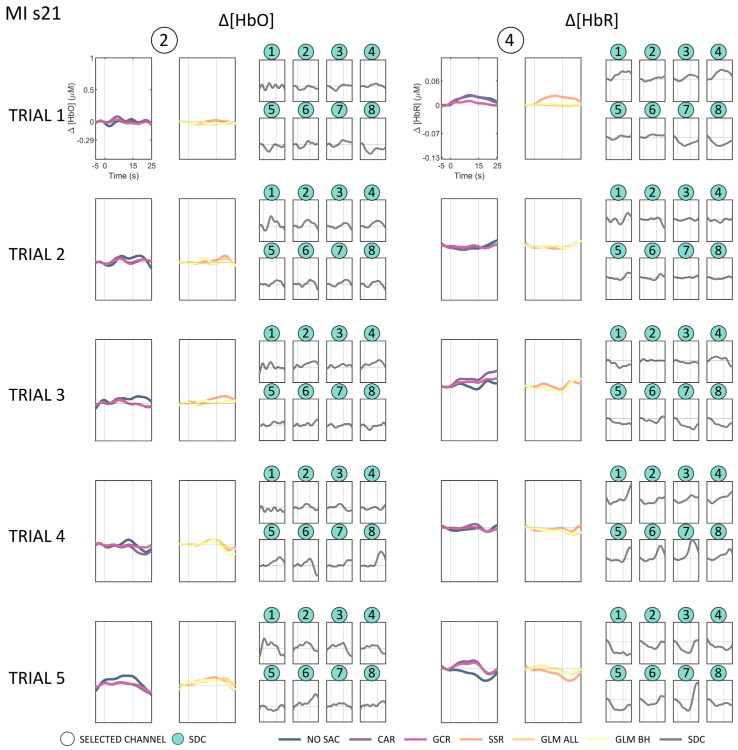
MI s21
Δ[HbO]
Δ[HbR]
2
4
TRIAL 1
TRIAL 2
TRIAL 3
TRIAL 4
TRIAL 5
Δ [HbO] (μM)
Δ [HbR] (μM)
Time (s)
SELECTED CHANNEL
SDC
NO SAC
CAR
GCR
SSR
GLM ALL
GLM BH
SDC

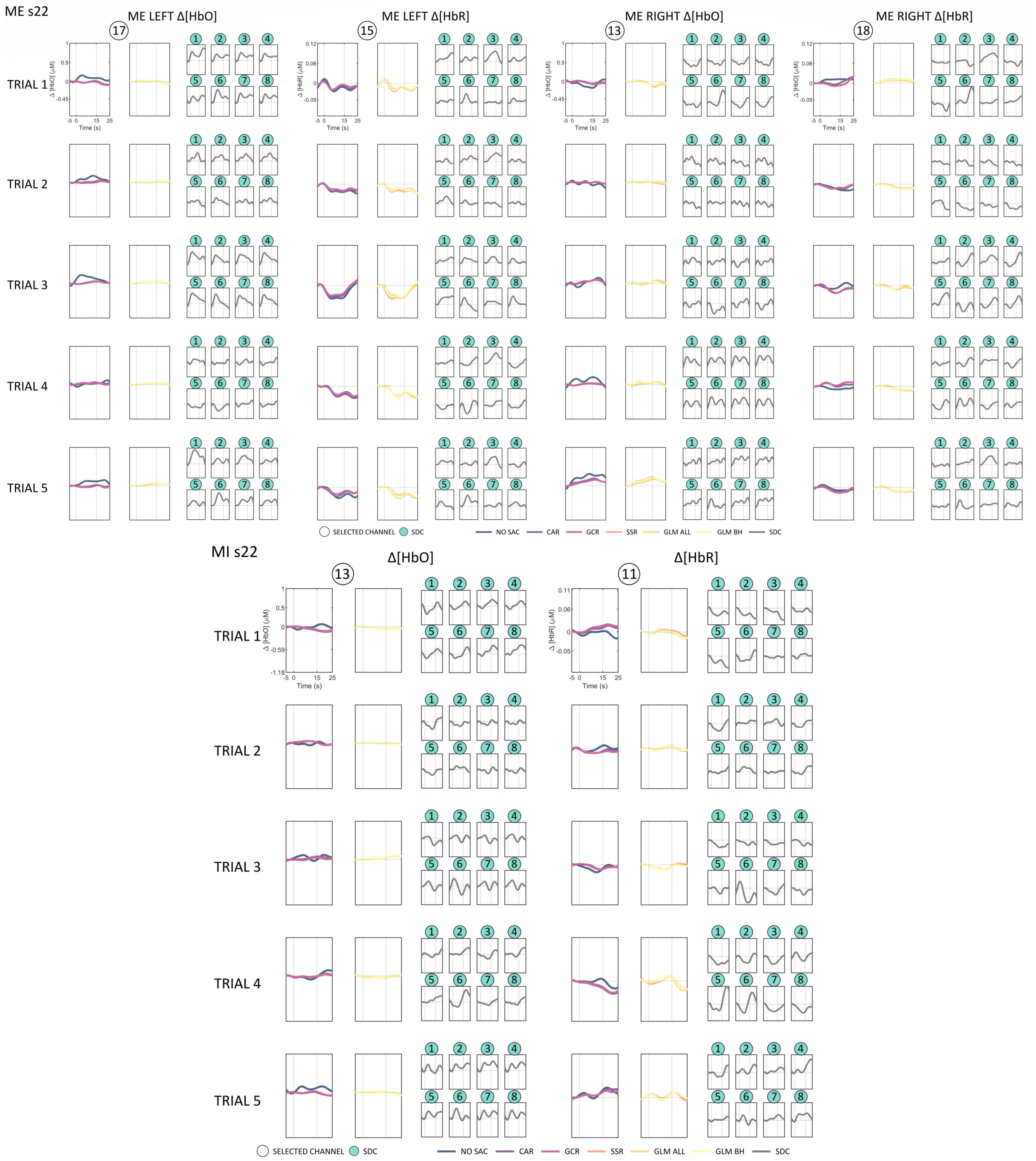
ME s22
ME LEFT Δ[HbO]
ME LEFT Δ[HbR]
ME RIGHT Δ[HbO]
ME RIGHT Δ[HbR]
TRIAL 1
TRIAL 2
TRIAL 3
TRIAL 4
TRIAL 5
Time (s)
SELECTED CHANNEL
SDC
NO SAC
CAR
GCR
SSR
GLM ALL
GLM BH
SDC
MI s22
Δ[HbO]
Δ[HbR]
TRIAL 1
TRIAL 2
TRIAL 3
TRIAL 4
TRIAL 5
Time (s)
SELECTED CHANNEL
SDC
NO SAC
CAR
GCR
SSR
GLM ALL
GLM BH
SDC

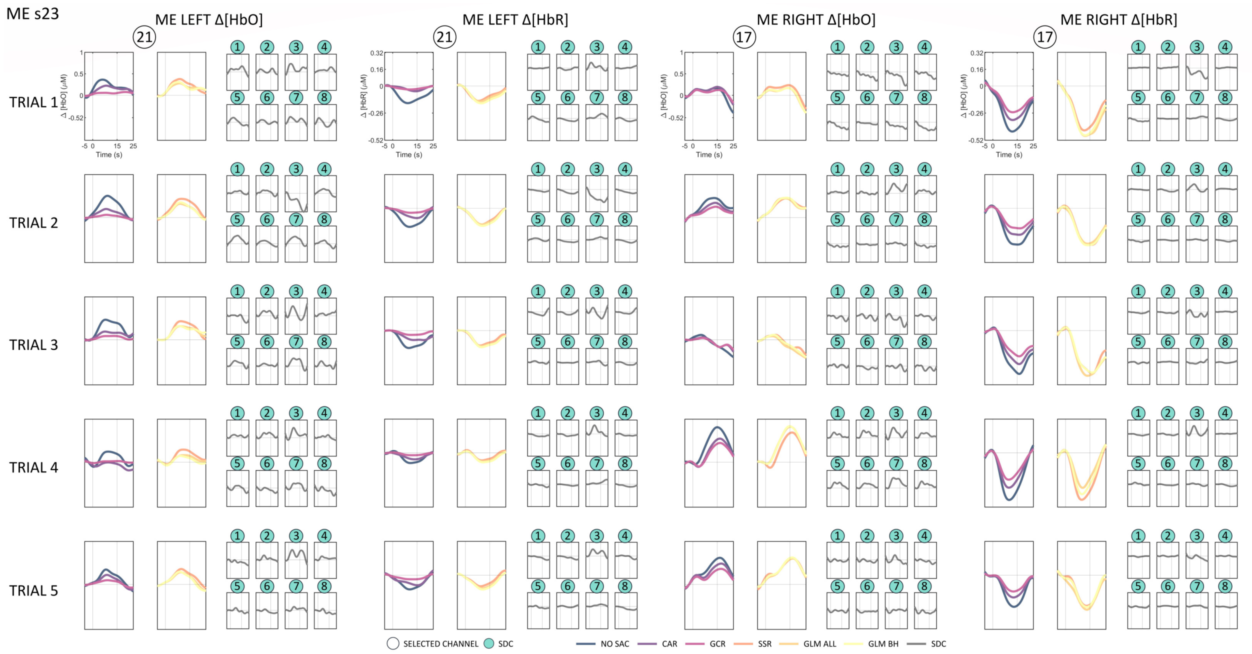
ME s23
ME LEFT Δ[HbO]
ME LEFT Δ[HbR]
ME RIGHT Δ[HbO]
ME RIGHT Δ[HbR]
21
21
17
17
TRIAL 1
TRIAL 2
TRIAL 3
TRIAL 4
TRIAL 5
Δ [HbO] (μM)
Δ [HbR] (μM)
Time (s)
SELECTED CHANNEL
SDC
NO SAC
CAR
GCR
SSR
GLM ALL
GLM BH
SDC

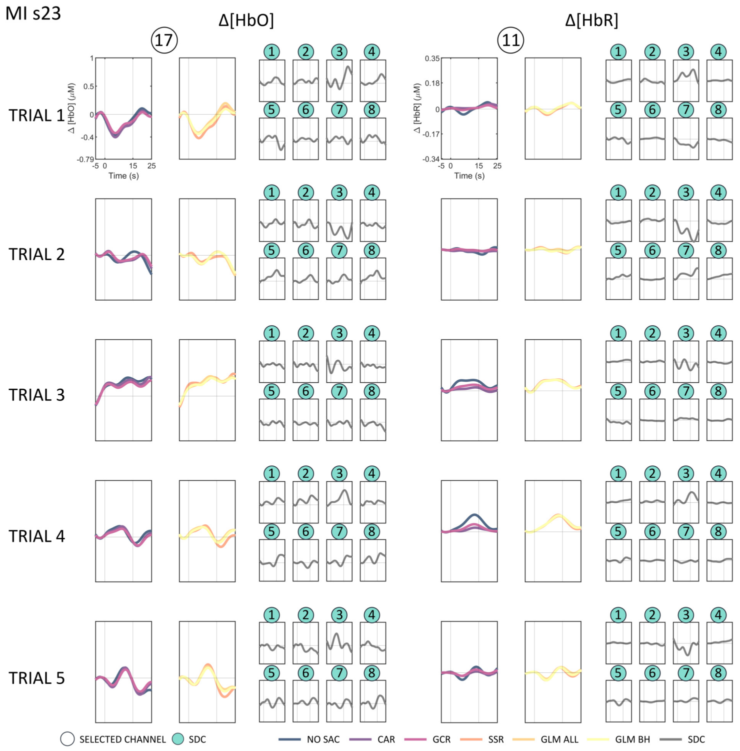
MI s23
Δ[HbO]
Δ[HbR]
17
11
TRIAL 1
TRIAL 2
TRIAL 3
TRIAL 4
TRIAL 5
Δ [HbO] (μM)
Δ [HbR] (μM)
Time (s)
SELECTED CHANNEL
SDC
NO SAC
CAR
GCR
SSR
GLM ALL
GLM BH
SDC

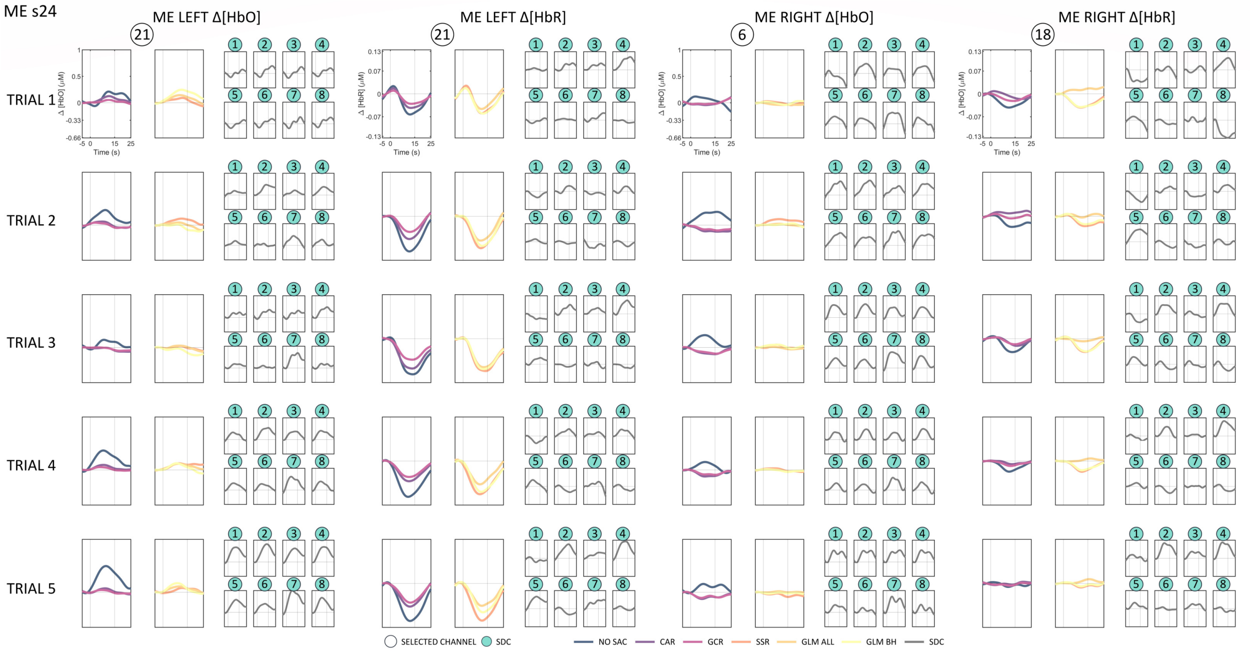
ME s24
ME LEFT Δ[HbO]
ME LEFT Δ[HbR]
ME RIGHT Δ[HbO]
ME RIGHT Δ[HbR]
TRIAL 1
TRIAL 2
TRIAL 3
TRIAL 4
TRIAL 5
Time (s)
SELECTED CHANNEL
SDC
NO SAC
CAR
GCR
SSR
GLM ALL
GLM BH
SDC

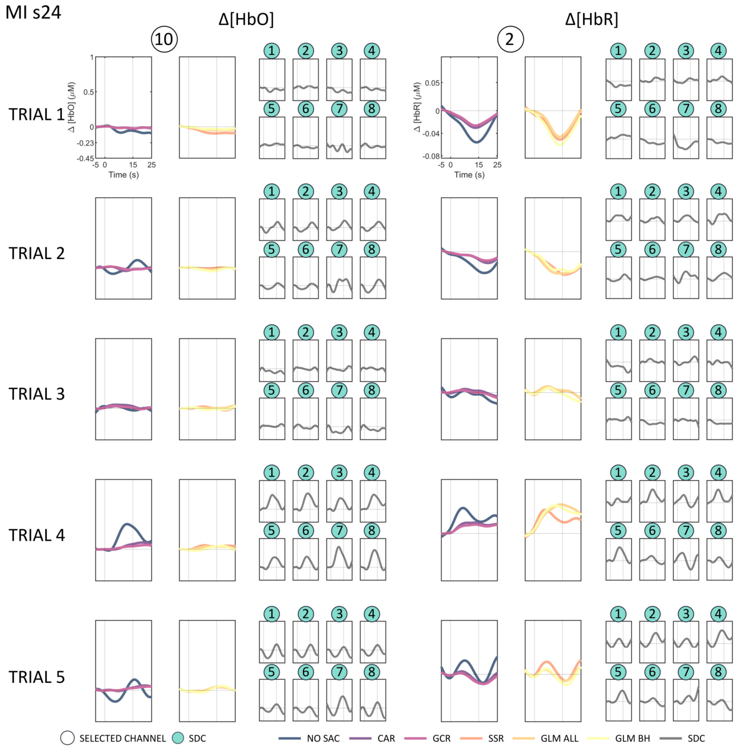
MI s24
Δ[HbO]
Δ[HbR]
TRIAL 1
TRIAL 2
TRIAL 3
TRIAL 4
TRIAL 5
Time (s)
SELECTED CHANNEL
SDC
NO SAC
CAR
GCR
SSR
GLM ALL
GLM BH
SDC
